# Evaluation of the SKILLZ intervention to promote HIV testing and contraception uptake in adolescent girls in Lusaka, Zambia: A cluster-randomized trial

**DOI:** 10.1371/journal.pgph.0005375

**Published:** 2025-10-29

**Authors:** Calvin Chiu, Chama Mulubwa, Boyd Mkandawire, Maggie Musonda, Ntenje Katota, Jenala Chipungu, David Boettiger, Nancy Padian, Carolyn Bolton Moore, Jenny X. Liu

**Affiliations:** 1 Institute for Health & Aging, University of California, San Francisco, California, United States of America; 2 Centre for Infectious Disease Research in Zambia, Lusaka, Zambia; 3 Grassroot Soccer, Zambia; 4 Kirby Institute, University of New South Wales, Sydney, Australia; Worcester Polytechnic Institute, UNITED STATES OF AMERICA

## Abstract

Adolescent girls are at disproportionately high-risk for HIV and unintended pregnancy. Effective interventions to increase uptake of HIV testing and contraceptives are urgently needed. Our cluster-randomized controlled trial in 46 schools in Lusaka, Zambia evaluated the SKILLZ intervention: (a) 12 after-school sessions of sexuality and sexual and reproductive health (SRH) education culminating in a community “graduation” soccer event where a pop-up clinic offered HIV testing and contraception; (b) for those HIV positive, coach-assisted linkage to HIV care, interpersonal group psychotherapy, or youth-friendly SRH services; and (c) community-based distribution of HIV self-testing and contraceptives. We surveyed randomly sampled Grade 11 girls at baseline, 6, and 12 months to measure self-reported uptake of HIV/SRH products and services. We estimated intention-to-treat modified poisson regressions on uptake of (a) HIV testing and any contraceptive method within the previous 6 months, (b) HIV testing by modality and contraceptive uptake by method, and (c) new adoption and discontinuation. Between March 2021 and June 2022, 1,019 girls were enrolled from 23 control schools and 1,134 enrolled from 23 intervention schools; 79% of surveyed girls in intervention schools participated in SKILLZ, 71% attended at least 8/12 sessions to “graduate”. At 6 months, 600 (59%) participants in intervention schools and 307 (37%) participants in control schools self-reported HIV testing within the previous 6 months (risk difference: 22%, 95% CI: 14, 29); 281 (37%) participants in intervention schools and 200 (30%) participants in control schools had reported using contraception within the previous 6 months (6%, 95% CI: -2, 14). Effects on HIV testing and contraception are sustained at 12 months. SKILLZ increased reported uptake of HIV testing and contraception among in-school adolescent girls. Further research is needed to understand treatment mechanisms and heterogeneity to tailor the intervention before implementation at scale. The trial is registered with ClinicalTrials.gov, NCT04429061.

## Introduction

In sub-Saharan Africa, adolescent girls and young women (15–24) face the combined threat of HIV and unintended pregnancy, leading to school drop-outs and worse long-term wellbeing [1]. Compared to their male counterparts, adolescent girls are twice as likely to acquire HIV infection [2] (4x in Zambia [3]) and have poor adherence to treatment should they test positive [4]. Among sexually active 15–24 year old women, HIV prevalence is three times higher (5.6%) than among similarly aged men (1.8%) [5]. Limited uptake of contraception increases the risk of unintended pregnancies, which affects 29% of women in sub-Saharan Africa (45% of women in Zambia) [6]. Despite many public health services being freely available [7], adolescent girls struggle to access critical prevention tools such as HIV testing and contraception [8]. Effective demand remains low due to poor awareness of sexual and reproductive health (SRH) and service options [9], stigmatizing attitudes in the community and among health providers [10], and power imbalances with partners for negotiating safe sex [11]. Thus, there is an urgent need for social and behavioural interventions that increase adolescent girls’ access to HIV testing and contraception [12].

Amid this context, we designed our study to fill three gaps in the literature. First, past social and behavioral interventions have had varying degrees of success, partially due to the lack of integrated interventions that address both HIV prevention and contraception [13]. Non-integrated services create barriers to HIV care by limiting access, increasing patient burden, and causing fragmented care due to poor coordination among providers, higher operational costs, and lack of standardized data systems. Additionally, they contribute to stigma, delays in treatment initiation, unmet contraceptive needs, and overburdened healthcare workers, ultimately reducing service efficiency and patient outcomes [13–15]. Studies show that when HIV services have been integrated with maternal, neonatal and child health, nutrition and family planning services [14], and non-communicable diseases [15], health and behavioural outcomes improve. Second, recent reviews have found limited evidence for the effectiveness of behavioural interventions in reducing HIV incidence for young women in sub-Saharan Africa [16] and social and behaviour change communication interventions for improving service utilisation, including HIV testing and contraception [17]. Given the challenges of objectively measuring behavioral outcomes including the difficulty of collecting administrative data on service delivery across multiple access points, particularly for those related to stigmatized sexual activity, much of the existing evidence has focused on outcomes along the awareness-intent spectrum, such as awareness, knowledge and risk perception [17]. While reviews of contraception interventions have found combined approaches that addressed both user and service provision issues to be effective, few rigorous studies measure contraceptive use behaviour – a recent systematic review found only 9 studies with “Very Low” GRADE evidence [18,19]. Third, although curriculum-based sex and HIV education programs have delayed or decreased sexual behaviour [20] and school-based sex and SRH education have increased knowledge and self-efficacy for refusing sex or using condoms [21,22], evidence on uptake of HIV testing or contraception (beyond self-reported condom-use) is limited. Despite potential concerns around stigma and inability to target at risk girls who have dropped out of school, school-based interventions remain the most convenient and scalable method of reaching adolescent girls, especially in sub-Saharan Africa [23].

To fill these gaps, we conducted an evaluation of a school-based intervention for adolescent girls to improve uptake of HIV testing and contraception, adding to the literatures on integrated interventions that address HIV prevention and contraception, social and behavior change interventions, and curriculum-based sex, SRH and HIV education programs by providing evidence on self-reported uptake of HIV testing and contraception. SKILLZ is a peer-led, sports-based program for empowering adolescent girls implemented by Grassroot Soccer (GRS) throughout Southern Africa. Sports-based HIV prevention interventions have shown short-term effects on HIV-related knowledge, stigma reduction, self-efficacy, and self-reported condom use [24,25], and previous pilots of SKILLZ have shown effects on increased knowledge and attitudes regarding HIV risk among participants [26], but none have been evaluated using a randomized controlled trial. Our study builds on this evidence base. Designed to simultaneously increase demand (through improved knowledge, awareness and empowerment) and supply (through more convenient access to services), we hypothesised that adolescent girls receiving SKILLZ will increase uptake of HIV testing and contraception. We report the trial’s main effects; implementation and process results will be reported in complementary manuscripts.

## Materials methods

### Ethics statement

The study was reviewed and approved by the University of Zambia Biomedical Research Ethics Committee (REF. NO. 004-01-19) and the Institutional Review Board of the University of Alabama, Birmingham (IRB-300002251). All study participants were informed of the study procedures and gave written informed consent or assent along with parental written consent for those under age 18.

### Study design and participants

We conducted a Type 1 hybrid implementation-effectiveness cluster randomized controlled trial [27] to evaluate SKILLZ on the self-reported uptake of HIV testing and contraception across 46 secondary schools in densely populated areas of Chilanga, Chongwe, Kafue, and Lusaka districts of Zambia. HIV prevalence in Zambia is 11.1% nationally [5] (16.5% in Lusaka district [28]) and three times higher among sexually active 15–24 year old women compared to men (5.6% vs 1.8%) [5]. Unmet need for family planning among women aged 15–19 is 12.9% (58.8% among all sexually active unmarried women), and 61% of young women report having had sex before age 18 [5].

Based on previous engagement with the Ministry of Health through their district offices and their explicit approval for approaching potential schools for study participation, 68 schools were identified as possible study sites by GRS. Schools with fewer than 40 female Grade 11 students based on official school class rosters were excluded (N = 22, 32%). GRS contacted each school and obtained permission to be included; all 46 schools approached agreed to participate. Notably, the COVID-19 pandemic affected study operations and intervention implementation. Zambia experienced three waves of COVID-19 and accompanying school closures (Figure J in S1 Text). When schools were open, extra-curricular activities involving over 30 individuals were restricted. During school closures, all in-person study activities were suspended. This study is reported per CONSORT guidelines.

All female students in Grade 11 enrolled in a participating school aged 16 or above at the time of recruitment were eligible for the evaluation. Study participation did not require any prior expression of interest in soccer. Study researchers provided study information to teachers and all Grade 11 female students and asked to meet with 30 students randomly pre-selected from class rosters. Those who were i) not present or reachable after three attempts, ii) did not receive parental consent (<18 years old), iii) declined to participate, or iv) ineligible due to age were replaced with students from the pre-specified randomly ordered back-up list. We continued enrolling eligible consenting students with replacement until we reached enrolment targets for each school or ran out of eligible students to enrol. All study participants were informed of the study procedures and gave written informed consent or assent along with parental written consent for those under age 18. Three study researchers described the purpose and nature of the study, emphasized the voluntary nature of participation and that refusing to participate in the study did not affect eligibility to participate in SKILLZ among students at schools assigned to the intervention arm, and answered all questions from study participants. Participants were informed that all responses would be kept strictly confidential and accessed only by the research team, that all personal identifiers would be anonymized, and that study findings would be shared in both aggregated and disaggregated forms while ensuring that no individual could be identified based on their responses. All communications were conducted in English, Bemba and Nyanja, depending on which language the participant stated they were most comfortable with. The consenting process and Baseline survey combined took approximately 45–60 minutes. No incentives were provided for completing any of the study activities. For participants under age 18, parental consent was obtained either physically or over the phone using a consent form in the language of their choice before participants provided assent. Study researchers explained the study and the involvement of their children and were available to answer any questions. Parents were encouraged to contact the Principal Investigator using the contact details provided (phone number and email address) if they had any further questions after the initial discussion. The assent process involved the following: participants were approached and given clear information about the study background, purpose, and procedures, including both surveys and in-depth interviews. The explanation also covered potential risks, benefits, and the lack of any financial compensation, clarifying that participation was entirely voluntary and that refusing would not affect them in any way. To ensure understanding, a set of standardized questions was used to assess the girls’ comprehension. Only those who answered all questions correctly were asked to provide assent, after which they were enrolled in the study.

Midway through conducting the baseline survey, we increased recruitment targets to 50 participants per school from 30 due to concerns regarding higher than anticipated attrition due to COVID-19. Among 22 schools where baseline surveys were completed, additional participants were recruited and surveyed at the 6-month follow-up. In 24 schools where recruitment occurred after we increased recruitment targets, 50 participants were recruited at baseline. Among schools that did not have 50 eligible consenting participants (N = 18), we enrolled all eligible consenting participants.

### Randomization and masking

The 46 schools available for study were randomly assigned (1:1) to the intervention and control arms, stratified by district, location (urban vs rural), remoteness (quartiles of distance from the local District Education Board), number of students (quartiles), and school type (co-education vs single sex). In addition, schools were randomly assigned (15:8) to two phases of implementation and data collection across both study arms to accommodate logistical constraints in the number of schools that the intervention could simultaneously be implemented at. GRS program managers contacted school administrators to inform them of their randomized assignment, explain implementation procedures, schedule implementation and evaluation activities, and obtain current Grade 11 enrolment rosters. Randomization was conducted using Stata Version 13.0 by the research team at the University of California. There was no masking for this study. Schools were randomly assigned before individual study participants were recruited within each school. A geographical map of how schools are distributed by treatment assignment is provided in the Appendix (Figure K in S1 Text).

### Procedures

In the schools assigned to the control arm, participants received the standard curriculum delivered at school by teachers, which involves Comprehensive Sexuality Education [29] which has been discussed extensively elsewhere [30]. Participants did not receive any interventions from GRS and only received surveys at baseline, 6- and 12-month follow-ups. Additionally, schools assigned to the intervention arm received SKILLZ [26]. All female Grade 11 students were invited to participate in SKILLZ regardless of whether they were randomly selected for the evaluation. SKILLZ consisted of i) 12 after-school sessions of sexuality and SRH education delivered by coaches, culminating in a community “graduation” soccer event where a pop-up clinic offered HIV testing (clinician-administered blood-based test or oral fluid self-test) and contraception (oral contraception, injectables, and male and female condoms); ii) (for those HIV positive), coach-assisted linkage to HIV care, interpersonal group psychotherapy, or youth-friendly SRH services; and iii) community-based distribution of HIV self-testing and contraceptives (condoms, oral pills, 3-month injectables, emergency contraception). SKILLZ promoted HIV testing per Zambia Ministry of Health guidelines – re-testing 3 months after a first negative test, and then testing every 12-months subsequently if at low risk for HIV (every 3 months if at high risk). SKILLZ was designed to be scalable through integration into both school education and community-based clinical services. Coaches were lay persons recruited from the community who were young adult “peers” and offered training on the SKILLZ curriculum and certification in community-based distribution of HIV self-testing and contraceptives by GRS (approximately 1 coach was hired for every 25 girls). Described extensively elsewhere [31], the 12-week SKILLZ curriculum covered topics such as healthy relationships and goal setting, over successive one-hour session, embedding content into soccer-based activities (run by the same coaches) that were designed to be enjoyable, interactive and engaging. The “graduation” event consisted of a soccer tournament held across multiple schools where participants could receive HIV testing and contraception on the side, either during breaks or when they were not actively playing in the soccer game.

Study participants were surveyed at baseline prior to implementing SKILLZ, and at 6- and 12-months after baseline on demographics, participants’ experience with SKILLZ, SRH knowledge and service use, sexual and reproductive empowerment [11], and sexual behaviours. For privacy and to reduce social desirability bias, the survey was self-administered on electronic tablets with audio narrations available in English, Bemba, and Nyanja through earphones. Participants had high literacy levels and were skilled at using a tablet; the survey team was present to answer questions and resolve technical issues. Baseline surveys were conducted in person on school premises. For follow-up surveys, the study team visited each school at least three times to maximise response rates. Participants not found at school were tracked using contact information shared at baseline and given either an in-person self-administered survey or interviewer-administered phone survey for those not physically reachable. We extracted data on SKILLZ attendance from GRS administrative records using study participants’ unique identifiers and linked this to the survey data.

All study procedures occurred on a rolling basis at each school between March 9, 2021, and January 18, 2023. Due to the COVID-19 pandemic, study activities were paused between June 16 and August 25, 2021, and again from January 10 to February 4, 2022. The follow-up surveys were designed to be conducted 6- and 12-months after Baseline respectively, and all SKILLZ activities completed before the 6-month survey. In practice, follow-up surveys were delayed due to COVID-19 induced disruptions to different degrees by school – 90% of 6-month (12-month) follow-up surveys occurred within 9-months (15-months) of Baseline (Figure J in S1 Text) – which also resulted in differences in time since SKILLZ completion among respondents in the intervention arm.

### Outcomes

The primary outcomes were self-reported i) HIV testing and ii) use of any contraception within the previous 6 months, measured at 6- and 12-month follow-up. Secondary outcomes included adoption (i.e., testing for HIV or using contraception at 6 or 12 months, but not at baseline) and discontinuation (i.e., not testing for HIV or using contraception at 6 or 12 months, among those testing for HIV or using contraception at baseline), HIV testing modality (i.e., oral fluid HIV self-testing vs blood-based assisted testing), and use of specific contraceptive methods (i.e., male and female condoms, oral daily pills, emergency contraception, injectables (depot medroxyprogesterone acetate intra-muscular or subcutaneous), intrauterine devices, and implants). Our secondary outcomes were not pre-specified; we added them post-hoc to understand whether intervention effects varied by baseline behavior and by testing modality and specific contraceptive method.

### Statistical analysis

We designed the study to detect a minimum 20 percentage point difference in HIV testing and contraceptive use between participants in the intervention vs control arms with 80% power assuming HIV testing prevalence of 60% in the control arm with α = 0.05, 0.25 intra-cluster correlation (ICC), and 20% attrition informed by GRS administrative data from previous SKILLZ implementation in Zambia. With 46 schools available for randomization, we planned to recruit 30 participants per school (N = 1,380). However, the COVID-19 pandemic raised concerns of higher than anticipated attrition due to school closures and increased absences from school-based activities. We re-estimated sample size calculations assuming 30% attrition and 75% rate of participation in SKILLZ and increased the enrolment target to 50 participants per school (N = 2,300).

To test whether those recruited at baseline were different from those additionally recruited at the 6-month follow-up, we tested for differences in means for observable characteristics using linear regressions for an indicator variable for additional enrolment at 6 months, stratified by intervention arm, clustering standard errors at the school level.

For both primary and secondary outcomes, we used an intention-to-treat (ITT) approach and estimated modified poisson regression models with school level clustered standard errors. We included all variables that were found to be unbalanced at baseline by study arm as controls. Relative risks and 95% confidence intervals are reported. To examine the effect of the intervention among those who were adherent, we also estimated inverse probability of treatment weighted (IPTW) models [32], defining adherence as attending eight or more SKILLZ sessions out of 12 per GRS program criteria for “graduating”. We constructed stabilized inverse probability weights by predicting the probability of adherence using baseline participant characteristics, imputing means for variables with missing values. All analyses were done using Stata V17.0. The trial is registered with ClinicalTrials.gov, NCT04429061.

We conducted a variety of robustness checks. We repeated the primary analysis using a difference-in-differences specification to account for potential chance imbalances in the outcome variable at baseline, restricting the sample to those recruited at baseline only, imputing (with zeros and ones in separate models to estimate upper and lower bound effects) missing responses on outcomes to bound ITT sensitivity, controlling for enrolment at 6 months (versus baseline), and controlling for month of enrolment fixed effects. To examine whether the same respondents reported testing/using contraception at both 6- and 12-month follow-ups, we constructed descriptive tables examining the extent of overlap for each outcome. To explore potential treatment heterogeneity, we plotted the difference in levels of our primary outcomes between 6- and 12-month follow-up and at baseline by school.

We completed a cost analysis of the two study arms from the health-care provider perspective. Resource use data were collected by use of an ingredients-based approach, in which each resource required for the intervention was identified and valued. Costs were mainly collated from the intervention implementers’ financial and utilisation documents and invoices provided to the trial administration. Contraception and HIV testing commodities were procured centrally and distributed by the trial management group. All costs were inflated to 2023 $US. The average total service cost per individual participant was calculated for each of the two study groups, as well as the difference in material costs. The difference in costs between study groups was compared against the differences in self-reported HIV testing uptake and contraception use. Cost data were compiled using Excel version 16.1 (Microsoft Software, Redmond, Washington, USA) and cost analyses were conducted in TreeAge Pro 2021 Version R2.1 (TreeAge Software, Williamstown, Massachusetts, USA). Further details on our economic evaluation methodology are described in Box M in S1 Text.

## Results

**Fig 1:**
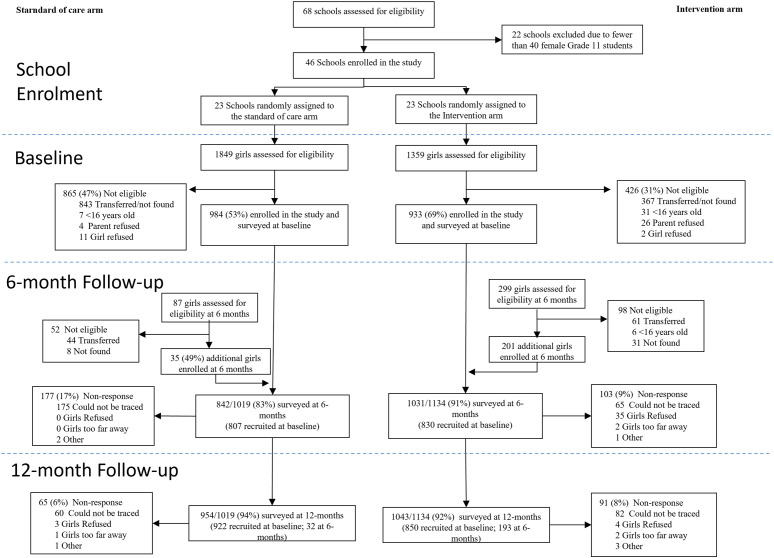
Trial profile.

A total of 3,594 students (N_control_ = 1936; N_intervention_ = 1658 across all phases) were selected for study recruitment across 46 participating schools (N_control_ = 23; N_intervention_ = 23): 1,354 students (N_control_ = 895, 46%; N_intervention_ = 459, 28%) were not found at school at the time of recruitment or had transferred out; 44 (N_control_ = 7, 0%; N_intervention_ = 37; 2%) were under age 16 and not eligible, 30 (N_control_ = 4, 0%; N_intervention_ = 26; 2%) did not receive parental consent, and 13 (N_control_ = 11, 1%; N_intervention_ = 2, 0%) refused to participate. Enrolment rates were higher in the intervention (1,134; 68%) vs the control arm (1,019; 53%) (Fig 1). Enrolment occurred in two phases: 1,917 participants (N_control_ = 984; N_intervention_ = 933) at baseline, and 236 participants (N_control_ = 35; N_intervention_ = 201) at 6-month follow-up. Response rates at 6-month follow-up were higher in the intervention arm (1,031; 91% vs 842; 83%) but similar at 12-month follow-up (1,043; 92% vs 1,031; 91%).

Note that there are substantially fewer additionally enrolled participants at the 6-month follow-up in control schools because implementation of the study was staggered across schools. The decision to increase enrolment from 30 to 50 students per school occurred prior to the baseline survey round of 15 out of 23 control schools, but only 9 out of 23 intervention schools, resulting in higher participant recruitment at baseline in more control schools and less need for additional enrolment at the 6-month follow-up.

Participant characteristics at baseline are shown in Table 1. Participants were 17 years old on average (SD: 1.36); 579 (31%) experienced food insecurity in the previous month; 461 (25%) had ever had sex; and 524 (42%) had received money or financial support from their sexual partner. 697 (37%) had tested for HIV and 345 (20%) had used contraception within the previous 6 months, both of which were higher among participants in control schools although differences were not statistically significant. Participants in the control arm also had higher rates of reported food insecurity, sexual activity, and pregnancy testing, although difference were not statistically significant. Participants in the control arm had statistically significantly higher empowerment scores and were more likely to be employed/earn income, suggesting potential differences in socio-economic status. Compared to those enrolled at 6-months, baseline enrolees in the intervention arm had slightly higher HIV knowledge and SRH empowerment scores, and lower levels of food insecurity and HIV positivity rates (though the latter is difficult to interpret given the small sample size) (Table A in S1 Text). Compared to those enrolled at 6-months, baseline enrolees in the control arm had lower rates of reported contraceptive use and HIV testing and higher pregnancy rates, though these differences are difficult to interpret given the small sample size (only 35 participants were additionally enrolled at 6 months in the control arm).

**Table 1:** Baseline characteristics of the intention-to-treat population.

	Control (N = 984)			Intervention (N = 933)			Total (N = 1918)		
Characteristics	Mean	SD	N	Mean	SD	N	Mean	SD	P-value
Age^1^	17.4	1.5	984	17.2	1.2	933	17.3	1.4	0.084
HIV knowledge (Correct out of 7)	5.36	1.12	984	5.29	1.16	933	5.33	1.14	0.395
Total number of sexual partners^2^	0.65	3.08	984	0.78	7.35	933	0.72	5.58	0.617
SHREYA empowerment score (/ 105)	78	17	984	75	18	933	76	18	0.018*
	**n**	**%**	**N**	**n**	**%**	**N**	**n**	**%**	**N**
Employed/earns income	288	29%	982	198	21%	931	486	25%	0.004**
Experienced food insecurity in the previous month	327	34%	976	252	27%	918	579	31%	0.097
Ever had sex	249	26%	963	212	23%	918	461	25%	0.423
Ever received money/support from sexual partner	275	43%	639	249	42%	594	524	42%	0.747
Used contraception within the previous 6 months	205	23%	894	140	17%	814	345	20%	0.061
Ever pregnant	44	4%	979	31	3%	928	75	4%	0.378
Tested for pregnancy	172	17%	983	120	13%	930	292	15%	0.114
Friend ever pregnant	789	84%	942	746	84%	890	1,535	84%	0.973
Friend ever had abortion	385	60%	641	364	61%	599	749	60%	0.850
Ever had STI symptoms	128	13%	984	94	10%	933	222	12%	0.117
Ever tested for HIV	597	61%	978	506	55%	923	1,103	58%	0.084
Tested for HIV within previous 6 months	385	39%	975	312	34%	917	697	37%	0.187
Tested HIV+	17	3%	565	14	3%	481	31	3%	0.928

* p < 0.05, ** p < 0.01, *** p < 0.001. Data not available for all randomized individuals due to non-response. Mean refers to mean for continuous variables and n for categorical variables. N refers to denominators for each variable. P-values were generated from linear regressions of each variable on an indicator variable for treatment assignment, clustering standard errors at the school level.

^1^Inter-Quartile Range: 2 (Total); 2 (Control); 2 (Intervention)

^2^Median/Inter-Quartile Range: 0/1 (Total); 0/1 (Control); 0/1 (Intervention)

At 6 months, 600 (59%) and 281 (37%) intervention and control participants, respectively, had reported that they tested for HIV and 307 (37%) and 200 (30%) used contraception, within the previous 6 months (Table 2) – this represents a 22 percentage point (pp) increase in HIV testing (95% CI: 14, 29) and 6pp increase in contraceptive use (95% CI: -2, 14). At 12 months, 552 (54%) and 297 (35%) intervention and control participants respectively had tested for HIV and 361 (39%) and 214 (26%) used contraception within the previous 6 months – this represents a 15pp increase in HIV testing (95% CI: 7, 23) and 8pp increase in contraceptive use (95% CI: 1, 15). In intervention schools, 899/1134 (79%) participants attended at least one SKILLZ session and 808/1134 (71%) attended eight or more sessions out of 12 (per protocol). IPTW estimates for HIV testing were substantively similar, but estimated relative risks for contraception uptake were higher in magnitude than ITT estimates. Differences in primary outcomes between 6- and 12-month follow-ups and at baseline by school show substantial variation in HIV testing and contraceptive uptake (Figure L in S1 Text).

**Table 2:** Impact of SKILLZ on HIV testing and contraception.

		Intervention	Control	Intention-to-treat			IPTW	
Outcomes	ICC	n_1_/N_1_^#^ (%)	n_2_/N_2_^#^ (%)	Relative Risk (95% CI)^&^	N	Relative Risk (95% CI)^&^		N
Attended at least 1 session of SKILLZ		899/1134 (79%)	0/1019 (0%)					
Graduated from SKILLZ (8 + sessions attended)		808/1134 (71%)	0/1019 (0%)					
Tested for HIV within the previous 6 months								
At Baseline	0.068	312/917 (34%)	385/975 (39%)					
At 6-month follow-up	0.098	600/1018 (59%)	307/827 (37%)	1.60 (1.34, 1.91)	1845	1.65 (1.39, 1.96)		1845
At 12-month follow-up	0.086	552/1021 (54%)	361/918 (39%)	1.41 (1.17, 1.70)	1939	1.49 (1.23, 1.80)		1939
Used contraception within the previous 6 months								
At Baseline	0.032	140/814 (17%)	205/894 (23%)					
At 6-month follow-up	0.076	281/765 (37%)	200/661 (30%)	1.18 (0.92, 1.51)	1426	1.31 (1.03, 1.66)		1426
At 12-month follow-up	0.053	297/859 (35%)	214/816 (26%)	1.33 (1.07, 1.64)	1675	1.43 (1.16, 1.76)		1675

^#^Counts and proportions with the outcome, out of the total participants in each study arm that responded to each outcome variable.

^&^Each outcome was analysed separately comparing differences in responses at each follow-up time point between arms using modified poisson regression. The relative risk and 95% confidence intervals are reported. All models adjust for SHREYA empowerment score and employed/earns income due to chance imbalances at baseline. IPTW estimates are additionally weighted by stabilized inverse probability weights for meeting the per protocol definition of attending eight or more SKILLZ sessions to graduate. Standard errors are clustered at the school level.

Table 3 shows results for adoption and discontinuation of HIV testing and contraception. At 6 months, intervention participants were more likely to adopt (RD: 20%, 95% CI: 16, 25) but no less likely to discontinue (RD: -7%, 95% CI: -14, 0) HIV testing, compared to their control counterparts; at 12 months, intervention participants remained more likely to adopt (RD: 15%, 95% CI: 10%, 20%) and were less likely to discontinue (RD: -10%, 95% CI: -16, -4) HIV testing. Intervention participants were more likely to have tested using blood-based assisted methods at 6 (RD: 13%, 95% CI: 4, 21) and 12 months (RD: 14%, 95% CI: 6, 22), respectively. Intervention participants were more likely to have tested for HIV using oral fluid self-testing at 6-months (RD: 5%, 95% CI: 0, 10) and 12-months (RD: 3%, 95% CI: 0, 8) but neither result was statistically significant (Table 4).

**Table 3:** Impact of SKILLZ on adoption and discontinuation of HIV testing and contraception.

	Intervention	Control	Intention To Treat		IPTW	
Outcomes	n_1_/N_1_^#^ (%)	n_2_/N_2_^#^ (%)	Relative Risk (95% CI)^&^	N	Relative Risk (95% CI)^&^	N
Adopted HIV testing						
At 6-month follow-up	283/917 (31%)	102/975 (10%)	2.94 (2.32, 3.73)	1892	2.79 (2.19, 3.56)	1892
At 12-month follow-up	252/917 (27%)	122/975 (13%)	2.16 (1.67, 2.79)	1892	2.09 (1.61, 2.70)	1892
Discontinued HIV testing						
At 6-month follow-up	65/312 (21%)	107/385 (28%)	0.75 (0.55, 1.02)	697	0.76 (0.56, 1.02)	697
At 12-month follow-up	65/312 (21%)	118/385 (31%)	0.68 (0.53, 0.88)	697	0.64 (0.50, 0.82)	697
Adopted contraception						
At 6-month follow-up	131/814 (16%)	98/894 (11%)	1.54 (1.14, 2.09)	1708	1.61 (1.19, 2.18)	1708
At 12-month follow-up	144/814 (17%)	103/894 (12%)	1.56 (1.17, 2.08)	1708	1.52 (1.15, 2.01)	1708
Discontinued contraception						
At 6-month follow-up	31/140 (22%)	46/205 (22%)	1.01 (0.67, 1.53)	345	0.91 (0.60, 1.39)	345
At 12-month follow-up	42/140 (30%)	79/205 (39%)	0.79 (0.60, 1.05)	345	0.75 (0.56, 1.01)	345

^#^Counts and proportions with the outcome, out of the total participants in each intervention arm that responded to each outcome variable.

^&^Each outcome was analysed separately using modified poisson regression. Adoption refers to taking up HIV testing/contraception at 6 or 12 months but not at baseline. Discontinuation refers to not taking up HIV testing/contraception at 6 or 12 months among those who did at baseline. The Relative Risk on the intervention term and 95% confidence intervals are reported. All models adjust for SHREYA empowerment score and employed/earns income due to chance imbalances at baseline. IPTW estimates are additionally weighted by stabilized inverse probability weights for meeting the per protocol definition of attending eight or more SKILLZ sessions to graduate. Standard errors are clustered at the school level.

**Table 4:** Impact of SKILLZ on specific testing modalities and contraceptive methods.

	Intervention	Control	Intention To Treat		IPTW	
Outcomes	n_1_/N_1_^#^ (%)	n_2_/N_2_^#^ (%)	Relative Risk (95% CI)^&^	N	Relative Risk (95% CI)^&^	N
Tested for HIV using self-testing						
At 6-month follow-up	99/1031 (10%)	46/842 (5%)	1.94 (1.04, 3.60)	1873	1.90 (1.01, 3.58)	1873
At 12-month follow-up	81/1043 (8%)	48/954 (5%)	1.71 (0.93, 3.16)	1997	1.71 (0.92, 3.19)	1997
Tested for HIV using blood-based methods						
At 6-month follow-up	624/1031 (61%)	392/842 (47%)	1.27 (1.09, 1.49)	1873	1.32 (1.12, 1.55)	1873
At 12-month follow-up	655/1043 (63%)	459/954 (48%)	1.28 (1.11, 1.48)	1997	1.34 (1.16, 1.55)	1997
Condoms (male and female)						
At 6-month follow-up	213/1015 (21%)	116/825 (14%)	1.57 (1.17, 2.10)	1840	1.68 (1.26, 2.25)	1840
At 12-month follow-up	238/1024 (23%)	199/930 (21%)	1.18 (0.91, 1.54)	1954	1.29 (1.00, 1.66)	1954
Oral pill daily contraception						
At 6-month follow-up	38/1015 (4%)	30/827 (4%)	1.09 (0.62, 1.91)	1842	1.20 (0.69, 2.11)	1842
At 12-month follow-up	40/1027 (4%)	56/934 (6%)	0.72 (0.42, 1.24)	1961	0.87 (0.49, 1.56)	1961
Emergency contraception						
At 6-month follow-up	112/1009 (11%)	50/825 (6%)	2.16 (1.52, 3.07)	1834	2.44 (1.72, 3.46)	1834
At 12-month follow-up	138/1025 (13%)	93/926 (10%)	1.47 (1.09, 2.00)	1951	1.65 (1.22, 2.25)	1951
Used long-acting reversible contraceptives (LARCs)						
At 6-month follow-up	79/1025 (8%)	41/833 (5%)	1.58 (0.96, 2.59)	1858	1.71 (1.05, 2.78)	1858
At 12-month follow-up	76/1035 (7%)	55/943 (6%)	1.26 (0.75, 2.10)	1978	1.47 (0.90, 2.39)	1978

^#^Counts and proportions with the outcome, out of the total participants in each intervention arm that responded to each outcome variable.

^&^Each outcome was analysed separately using modified poisson regression. The Relative Risk on the intervention term and 95% confidence intervals are reported. All models adjust for SHREYA empowerment score and employed/earns income due to chance imbalances at baseline. IPTW estimates are additionally weighted by stabilized inverse probability weights for meeting the per protocol definition of attending eight or more SKILLZ sessions to graduate. Standard errors are clustered at the school level.

LARCs refer to depo-provera, Sayana Press, IUDs, and Implants.

For contraception, intervention participants were more likely to adopt (RD: 5%, 95% CI: 1, 10) but not significantly less likely to discontinue (RD: 0%, 95% CI: 9, -9) at 6 months compared to control participants. Results were maintained at 12 months. At 6 months, participants were more likely to have used condoms (RD: 8%, 95% CI: 3, 13) and emergency contraception (RD: 6%, 95% CI: 3, 10), but only results on emergency contraception were maintained at 12 months (RD: 5%, 95% CI: 0, 8). Results from additional contraceptive methods (withdrawal, natural, and abstinence, and pooled modern methods) are reported in Table B in S1 Text.

Our primary analysis for HIV testing is robust to using a difference-in-differences specification (Table C in S1 Text). In addition, our results are unchanged when restricting analysis to those initially enrolled at baseline (Table D in S1 Text). Our results are not sensitive to non-response bias (Table E in S1 Text) – note that those who did not respond to the contraceptive uptake question reported less underlying sexual activity (Table F in S1 Text). Our results are unchanged when controlling for timing of enrolment (Table G in S1 Text).

The overall cost of delivering the GRS intervention was $534,255. This was comprised of 51% staff costs, 3% administration, 15% travel, 12% equipment/maintenance, and 19% other costs. The average cost per participant in the intervention group was $472, and the average cost per participant in the control group was $6. Comparing these values against our adjusted 12-month effectiveness results, SKILLZ cost $2,006 per additional girl receiving an HIV test, and $2,362 per additional girl using contraception. Further results from our economic evaluation are described in Box M in S1 Text.

## Discussion

In our cluster randomized trial of a peer-led sports-based program for empowering adolescent girls, we found a substantial effect on participants’ self-reported use of HIV testing and contraception at 6 months, which persisted through 12 months, more than 9 months after “graduation”. SKILLZ also decreased discontinuation of HIV testing and contraception among participants, showing effects even among those already using services. To our knowledge, this is one of the first adolescent-specific social and behavioural interventions with demonstrated impact on both self-reported HIV testing and contraceptive use based on a randomized controlled trial [18]. The magnitude of our effects and their consistency across various sensitivity analyses is particularly significant given that the study occurred during the COVID-19 pandemic when regular school operations and health services were disrupted.

As a holistic intervention that integrates HIV prevention and contraception with SRH education, this result simultaneously contributes to several strands of literature. We show persistent increases in HIV testing and contraception when past social and behavioural HIV prevention interventions have found limited evidence for actual behaviour change [17] with few studies from low and-middle-income countries measuring contraceptive behaviours among adolescents [33]. Our integrated approach combining education and demand generation with improved service provision corroborates findings that integrated approaches are more effective. We build on studies of school-based SRH health education [22] and sports-based HIV prevention that show effects on upstream outcomes—HIV knowledge, stigma reduction, self-efficacy—and self-reported condoms use with evidence of downstream effects on HIV testing and other contraceptive use [21].

Our findings for differential effects across HIV testing modalities and contraceptive methods suggest further ways to meet adolescents’ SRH needs. Despite the emphasis on HIV self-testing in the SKILLZ curriculum, uptake of self-testing remained low (<10%), suggesting that self-testing has greater behaviour change barriers that should be explored. Despite the treatment effects on contraceptive use, overall levels of contraceptive use remained low (~30%) relative to unmet need [5] suggesting the importance of complementary interventions and further tailoring of SKILLZ to increase adolescents’ access to and uptake of contraception.

Further exploration into treatment heterogeneity and potential underlying mechanisms is needed to understand our results. There was substantial heterogeneity by school in both levels of HIV testing and contraception at baseline and changes between baseline and follow-up rounds. While changes were positive on average, results varied across schools. Some schools, primarily in the control arm, found decreases in HIV testing and contraception after baseline, which may have been influenced by secular trends in reduced access to SRH associated with COVID-19 [34]. Further investigation qualitatively with participating students and SKILLZ coaches is ongoing and will shed further light on differences in implementation fidelity across schools.

Consistent with previous work evaluating SKILLZ in sub-Saharan Africa [35], we found a large proportion of programme costs were recurring expenses, mostly comprised of staff costs. Overall, the average cost per participant in the intervention group was $472. Compared with no intervention, this equated to a cost of $2,006 per additional girl receiving an HIV test, and $2,362 per additional girl using contraception. [36]

Our study has several limitations. First, our outcomes are self-reported and affected by non-response bias. The self-report nature of our outcomes raises concerns around social desirability bias potentially leading to over-reporting of HIV testing and contraception use, especially among the intervention arm where participants understood the purpose of the SKILLZ intervention, leading to overestimating the intervention’s impact. Verifying outcomes using administrative data (e.g., clinic and pharmacy records) was infeasible given the size and duration of our study and the range of potential options to access services, especially regarding contraception. Second, there were some data inconsistencies, most notably around sexual behaviour (e.g., more participants reported receiving money and support from their sexual partner than reported ever having sex) which precluded the estimation of HIV testing and contraceptive use conditional on being sexually active. However, eliciting accurate responses about sexual activity is a known challenge, especially among stigmatized groups [36] like unmarried young women. The benefits of conducting a self-administered survey to reduce social desirability bias outweighed potential response inconsistencies for sensitive questions and incomprehension with unfamiliar concepts. Relatedly, we cannot definitively rule out the possibility that some participants may have misunderstood the recall period for our primary outcomes (HIV testing and contraceptive use within the last 6 months). However, the limited overlap of participants that reported HIV testing/contraceptive use at both 6 and 12-month follow-up suggests that the 6 and 12-month follow-up surveys captured differentiable responses consistent with respondents recognizing the different recall time periods asked about (Table I in S1 Text). Third, our study was severely affected by the COVID-19 pandemic. Though we increased recruitment to mitigate against higher attrition, we were unable to estimate the impact of the COVID-19 pandemic itself on the effectiveness of our interventions. The COVID-19-induced disruptions also led to different timing of follow-up surveys by arm (Figure J in S1 Text), which potentially affects comparability across arms. However, our primary results were unchanged in models that controlled for timing of enrolment (Table G in S1 Text). Fourth, there were chance imbalances between participants across arms despite the cluster-randomized design which may affect the interpretation of our results. At baseline (Table 1), participants in the intervention arm had lower SHREYA empowerment scores (p = 0.018, although the magnitude of differences were small) and were less likely to be employed/earn income (p = 0.004) or experience food insecurity (p = 0.097), which potentially suggests higher socioeconomic status and could confound our results (note that we interpret employment as indicative of low socioeconomic status in this context since participants were full-time Grade 11 students). Further, participants in the intervention arm reported lower rates of HIV testing (p = 0.187) and contraceptive use (p = 0.061) in the 6 months prior to baseline (but neither difference is statistically significantly different), which could make intervention participants more receptive to SKILLZ. Fifth, some costs were gathered from different sources (financial records, published estimates, site visit documentation from routine programme operations) and not expressly collected for study purposes, while other costs incurred in a trial setting may differ from true implementation costs. Also note that there is double counting of costs in our ICERs owing to the joint effectiveness of the intervention. One way to account for this is by saying that for every $2,362 spent on the intervention, there was one additional girl using contraception and 1.2 additional girls receiving HIV testing. Sixth, our study’s external validity is restricted to settings where soccer and sports-based programming is appropriate and feasible for adolescent girls. While there was broad interest in participation among general population Grade 11 school girls in our context (71% attended 8 or more sessions out of 12), this may not be the case in other contexts across sub-Saharan Africa. Seventh, given that study participants live in high density areas and may interact with each other outside of school settings, we cannot completely rule out the risk of contamination across study arms. However, given the nature of the intervention – after-school sessions of sexuality and sexual and reproductive health education occurring on site among Grade 11 students at each school respectively – it is highly improbable that study participants from Control schools would have been able to access the intervention, nor did we find evidence of this from the self-reported survey data or GRS administrative data. Given the difficulty of inducing behavior change, we do not believe that limited encounters between study participants outside of school is a major contributor to the observed treatment effects. Finally, our study was not powered to detect changes in the public health outcomes of HIV incidence and unintended pregnancy. However, demonstrating effectiveness on self-reported uptake of HIV testing and contraception remains a contribution to the literature on social and behavior change communications interventions given that existing evidence has focused on outcomes along the awareness-intent spectrum [17]. Relatedly, the intervention is targeted towards girls in school even though girls out of school may be at greater risk of HIV and unintended pregnancy, limiting the intervention’s ultimate public health impact. However, the intervention is intended to prevent one of the pathways toward school drop out by preventing HIV acquisition and unintended pregnancy.

Notwithstanding, our study has several strengths. We were powered to detect effects on HIV testing and contraceptive use from a prospective randomized controlled trial, building on previous studies that either examined intermediate outcomes or used less rigorous designs. We are also able to examine the persistence of treatment effects due to our long 12-month follow-up period and disaggregate effects by HIV testing modality and specific contraceptive method.

In summary, our results show that SKILLZ increased self-reported HIV testing and contraception use among adolescent girls, despite disruptions and complications related to the COVID-19 pandemic, which suggests that effects measured may be muted relative to a context without such disruptions. Our study context also underscores the importance of providing social and behavioural supports in times when education and health systems are stressed. To build on the evidence base before implementing similar interventions in other contexts, future research should consider measuring HIV testing and contraception use more rigorously using administrative data (e.g., contraceptive prescriptions and provider-reported HIV testing, if feasible in the context) to avoid issues around self-reported outcomes and potentially power to detect effects on the public health outcomes HIV incidence and unintended pregnancy. Our findings should be interpreted with caution given the limitations of self-reported measures, particularly around socially sensitive behavior, and the lack of data on long-term health outcomes (HIV incidence and unintended pregnancy) rather than service uptake measures.

## Supporting information

S1 Text Table A. Descriptive statistics comparing those recruited at baseline and those additionally enrolled at 6-month follow-up. Table B. Impact of SKILLZ on other contraceptive methods. Table C. Impact of SKILLZ on HIV testing and contraception using difference-in-difference models. Table D. Impact of SKILLZ on HIV testing and contraception among those recruited at baseline only. Table E. Sensitivity analysis of primary outcomes by non-response bias. Table F. Descriptive statistics comparing responders to non-responders for primary outcomes. Table G. Sensitivity analysis of primary outcomes to timing of enrolment. Table H. HIV testing and contraception uptake at the Graduation Event. Table I. HIV testing and contraception uptake at 6 vs 12 months. Figure J. Timeline of Data Collection and Implementation of SKILLZ at each school. Figure K. Geographical map of participating schools in Lusaka by treatment assignment. Number of schools in the Treatment/Control arms respectively aggregated at the ward level based on 2020 Constituency Boundaries. The base layer of the map is provided by OCHA Regional Office for Southern and Eastern Africa (ROSEA) via DMMU Zambia and hosted by The Humanitarian Data Exchange (https://data.humdata.org/dataset/zambia_adm_boundaries). Figure L. Heterogenous treatment effects by school. Box M: Additional detail on economic evaluation.
(PDF)

S2 Text Study protocol. (PDF)

S1 Checklist CONSERVE Checklist. The CONSERVE implementation tool is licensed under the Creative Commons Attribution-NonCommercial-NoDerivatives 4.0 International license.
(DOCX)

S2 Checklist CONSORT Checklist. Schulz KF, Altman DG, Moher D, for the CONSORT Group. CONSORT 2010 Statement: updated guidelines for reporting parallel group randomised trials. BMC Medicine. 2010;8:18. © 2010 Schulz et al. This is an Open Access article distributed under the terms of the Creative Commons Attribution License (http://creativecommons.org/licenses/by/2.0), which permits unrestricted use, distribution, and reproduction in any medium, provided the original work is properly cited.
(DOCX)
