# Supplementary material for: Evaluation of the SKILLZ intervention to promote HIV testing and contraception uptake in adolescent girls in Lusaka, Zambia: A cluster-randomized trial
Source: PLOS Glob Public Health. 2025 Oct 29;5(10):e0005375. doi: 10.1371/journal.pgph.0005375 (PMC12571267; doi:10.1371/journal.pgph.0005375)
Supplement: S1 Text — Table A. Descriptive statistics comparing those recruited at baseline and those additionally enrolled at 6-month follow-up. Table B. Impact of SKILLZ on other contraceptive methods. Table C. Impact of SKILLZ on HIV testing and contraception using difference-in-difference models. Table D. Impact of SKILLZ on HIV testing and contraception among those recruited at baseline only. Table E. Sensitivity analysis of primary outcomes by non-response bias. Table F. Descriptive statistics comparing responders to non-responders for primary outcomes. Table G. Sensitivity analysis of primary outcomes to timing of enrolment. Table H. HIV testing and contraception uptake at the Graduation Event. Table I. HIV testing and contraception uptake at 6 vs 12 months. Figure J. Timeline of Data Collection and Implementation of SKILLZ at each school. Figure K. Geographical map of participating schools in Lusaka by treatment assignment. Number of schools in the Treatment/Control arms respectively aggregated at the ward level based on 2020 Constituency Boundaries. The base layer of the map is provided by OCHA Regional Office for Southern and Eastern Africa (ROSEA) via DMMU Zambia and hosted by The Humanitarian Data Exchange (https://data.humdata.org/dataset/zambia_adm_boundaries). Figure L. Heterogenous treatment effects by school. Box M: Additional detail on economic evaluation. (PDF) [file pgph.0005375.s001.pdf]

# S1 Text

## Table A in S1 Text

**Descriptive statistics comparing those recruited at baseline and those additionally enrolled at 6-month follow-up**

| Intervention Arm | | | | | | | | | | | |
|---|---|---|---|---|---|---|---|---|---|---|---|
| | Total (N=1031) | | | Baseline Cohort (N=830) | | | Additional Enrolled (N=201) | | | Difference in means | P-value |
| **Characteristics** | **Mean** | **SD** | **N** | **Mean** | **SD** | **N** | **Mean** | **SD** | **N** | | |
| Age | 17.7 | 1.2 | 1,031 | 17.7 | 1.2 | 830 | 17.4 | 1.4 | 201 | 0.3 | 0.058 |
| HIV Knowledge (Correct out of 7) | 5.51 | 1.19 | 1,031 | 5.57 | 1.19 | 830 | 5.25 | 1.13 | 201 | 0.322 | 0.003** |
| Total number of sexual partners | 0.64 | 1.83 | 1,030 | 0.66 | 1.78 | 829 | 0.58 | 2.02 | 201 | 0.080 | 0.684 |
| SHREYA empowerment score (/ 105) | 84 | 18 | 1,031 | 85 | 18 | 830 | 80 | 19 | 201 | 5 | 0.015* |
| | **n** | **%** | **N** | **n** | **%** | **N** | **n** | **%** | **N** | | |
| Employed/earns income | 298 | 29% | 1,026 | 233 | 28% | 826 | 65 | 33% | 200 | -0.043 | 0.413 |
| Experienced food insecurity in the previous month | 285 | 28% | 1,013 | 215 | 26% | 816 | 70 | 36% | 197 | -0.092 | 0.024* |
| Ever had sex | 333 | 33% | 1,009 | 280 | 34% | 814 | 53 | 27% | 195 | 0.072 | 0.131 |
| Received money/support from sexual partner (within the previous 6 months) | 311 | 50% | 626 | 255 | 50% | 505 | 56 | 46% | 121 | 0.042 | 0.594 |
| Used contraception within the previous 6 months | 281 | 37% | 765 | 210 | 34% | 612 | 71 | 46% | 153 | -0.121 | 0.060 |
| Ever pregnant | 20 | 2% | 1,026 | 17 | 2% | 827 | 3 | 2% | 199 | 0.005 | 0.530 |
| Tested for pregnancy | 125 | 12% | 1,022 | 100 | 12% | 825 | 25 | 13% | 197 | -0.006 | 0.814 |
| Friend ever pregnant | 635 | 69% | 917 | 510 | 69% | 741 | 125 | 71% | 176 | -0.022 | 0.615 |
| - Friend ever abortion | 225 | 46% | 490 | 184 | 47% | 389 | 41 | 41% | 101 | 0.067 | 0.126 |
| Ever STI symptoms | 132 | 13% | 1,031 | 112 | 13% | 830 | 20 | 10% | 201 | 0.035 | 0.108 |
| Tested for HIV within previous 6 months | 600 | 59% | 1,018 | 487 | 60% | 817 | 113 | 56% | 201 | 0.034 | 0.436 |
| - Tested HIV+ | 9 | 2% | 506 | 3 | 1% | 404 | 6 | 6% | 102 | -0.051 | 0.018* |
| **Control Arm** | | | | | | | | | | | |
| | Total (N=842) | | | Baseline Cohort (N=807) | | | Additional Enrolled (N=35) | | | Difference in means | P-value |
| **Characteristics** | **Mean** | **SD** | **N** | **Mean** | **SD** | **N** | **Mean** | **SD** | **N** | | |

| | | | | | | | | | | | |
|---|---|---|---|---|---|---|---|---|---|---|---|
| Age | 17.8 | 1.4 | 842 | 17.8 | 1.4 | 807 | 17.8 | 1.1 | 35 | 0.039 | 0.843 |
| HIV Knowledge (Correct out of 7) | 5.47 | 1.19 | 842 | 5.48 | 1.19 | 807 | 5.34 | 1.24 | 35 | 0.133 | 0.385 |
| Total number of sexual partners | 0.57 | 2.09 | 841 | 0.57 | 2.12 | 806 | 0.71 | 1.20 | 35 | -0.149 | 0.602 |
| SHREYA empowerment score (/ 105) | 80 | 18 | 842 | 80 | 18 | 807 | 78 | 15 | 35 | 2 | 0.230 |
| | **n** | **%** | **N** | **n** | **%** | **N** | **n** | **%** | **N** | | |
| Employed/earns income | 244 | 29% | 836 | 234 | 29% | 801 | 10 | 29% | 35 | 0.006 | 0.898 |
| Experienced food insecurity in the previous month | 264 | 32% | 831 | 255 | 32% | 979 | 9 | 26% | 34 | 0.055 | 0.324 |
| Ever had sex | 229 | 28% | 828 | 217 | 27% | 793 | 12 | 34% | 35 | -0.069 | 0.504 |
| Received money/support from sexual partner | 218 | 41% | 526 | 210 | 42% | 506 | 8 | 40% | 20 | 0.015 | 0.894 |
| Used contraception within the previous 6 months | 200 | 30% | 661 | 188 | 30% | 633 | 12 | 43% | 28 | -0.132 | 0.026* |
| Ever pregnant | 16 | 2% | 833 | 16 | 2% | 799 | 0 | 0% | 34 | 0.020 | 0.000*** |
| Tested for pregnancy | 86 | 10% | 836 | 78 | 10% | 801 | 8 | 23% | 35 | -0.131 | 0.075 |
| Friend ever pregnant | 544 | 71% | 762 | 524 | 72% | 731 | 20 | 65% | 31 | 0.072 | 0.115 |
| - Friend ever abortion | 207 | 50% | 416 | 197 | 49% | 398 | 10 | 56% | 18 | -0.061 | 0.402 |
| Ever STI symptoms | 81 | 10% | 842 | 79 | 10% | 807 | 2 | 6% | 35 | 0.041 | 0.533 |
| Tested for HIV within previous 6 months | 307 | 37% | 827 | 287 | 36% | 793 | 20 | 59% | 34 | -0.226 | 0.000*** |
| - Tested HIV+ | 1 | 0% | 294 | 1 | 0% | 274 | 0 | 0% | 20 | 0.004 | 0.333 |

P-values were generated from linear regressions of each variable on an indicator variable for additional enrolment, clustering standard errors at the school level.

Note that there are substantially fewer additionally enrolled participants at 6-month follow-up in the Control Arm because implementation of the study was staggered across schools. The decision to increase enrolment due to concerns around increased loss-to-follow-up from the COVID-19 pandemic occurred prior to the Baseline of 15/23 Control schools but only 9/23 Intervention schools so we managed to recruit more participants at Baseline in more Control schools, reducing the need for additional enrolment at 6-month follow-up.

## Table B in S1 Text

**Impact of SKILLZ on other contraceptive methods**

| | Intervention | Control | Intention To Treat | | IPTW | |
|---|---|---|---|---|---|---|
| **Outcomes** | **$n_1/N_1$# (%)** | **$n_2/N_2$# (%)** | **Relative Risk (95% CI) &** | **N** | **Relative Risk (95% CI) &** | **N** |
| Withdrawal | | | | | | |
| - At 6-month follow-up | 134/953 (14%) | 91/799 (11%) | 1.31 (0.98, 1.75) | 1752 | 1.43 (1.07, 1.91) | 1752 |
| - At 12-month follow-up | 165/980 (17%) | 143/910 (16%) | 1.14 (0.90, 1.44) | 1890 | 1.21 (0.96, 1.54) | 1890 |
| Natural (rhythm or calendar) | | | | | | |
| - At 6-month follow-up | 185/975 (19%) | 136/810 (17%) | 1.18 (0.95, 1.47) | 1785 | 1.25 (1.01, 1.55) | 1785 |
| - At 12-month follow-up | 169/991 (17%) | 149/907 (16%) | 1.09 (0.86, 1.39) | 1898 | 1.19 (0.94, 1.52) | 1898 |
| Abstinence | | | | | | |
| - At 6-month follow-up | 39/960 (4%) | 38/794 (5%) | 0.86 (0.59, 1.25) | 1754 | 0.93 (0.64, 1.35) | 1754 |
| - At 12-month follow-up | 31/979 (3%) | 18/909 (2%) | 1.50 (0.87, 2.61) | 1888 | 1.60 (0.90, 2.83) | 1888 |
| Used modern methods of contraception | | | | | | |
| - At 6-month follow-up | 297/1026 (29%) | 172/836 (21%) | 1.48 (1.17, 1.88) | 1862 | 1.61 (1.28, 2.03) | 1862 |
| - At 12-month follow-up | 329/1038 (32%) | 267/948 (28%) | 1.20 (0.96, 1.50) | 1986 | 1.29 (1.04, 1.60) | 1986 |

#Counts and proportions with the outcome, out of the total participants in each intervention arm that responded to each outcome variable.

&Each outcome was analysed separately using modified poisson regression. The Relative Risk on the intervention term and 95% confidence intervals are reported. All models adjust for SHREYA empowerment score and employed/earns income due to chance imbalances at baseline. IPTW estimates are additionally weighted by stabilized inverse probability weights for meeting the per protocol definition of attending eight or more SKILLZ sessions to graduate. Standard errors are clustered at the school level.

## Table C in S1 Text

**Impact of SKILLZ on HIV testing and contraception using difference-in-difference models**

| | | Intervention | Control | Intention To Treat | | IPTW | |
|---|---|---|---|---|---|---|---|
| **Outcomes** | **ICC** | **$n_1/N_1$[#] (%)** | **$n_2/N_2$[#] (%)** | **Relative Risk (95% CI)** [&] | **N** | **Relative Risk (95% CI)** [&] | **N** |
| Attended at least 1 session of SKILLZ | | 899/1134 (79%) | 0/1019 (0%) | | | | |
| - Graduated from SKILLZ (8+ sessions attended) | | 808/1134 (71%) | 0/1019 (0%) | | | | |
| Tested for HIV within the previous 6 months | | | | | | | |
| - At Baseline | 0.068 | 312/917 (34%) | 385/975 (39%) | | | | |
| - At 6-month follow-up | 0.098 | 600/1018 (59%) | 307/827 (37%) | 1.84 (1.55, 2.19) | 3737 | 1.68 (1.42, 1.99) | 3737 |
| - At 12-month follow-up | 0.086 | 552/1021 (54%) | 361/918 (39%) | 1.63 (1.35, 1.96) | 3831 | 1.51 (1.26, 1.81) | 3831 |
| Used contraception within the previous 6 months | | | | | | | |
| - At Baseline | 0.032 | 140/814 (17%) | 205/894 (23%) | | | | |
| - At 6-month follow-up | 0.076 | 281/765 (37%) | 200/661 (30%) | 1.54 (1.22, 1.93) | 3134 | 1.37 (1.09, 1.71) | 3134 |
| - At 12-month follow-up | 0.053 | 297/859 (35%) | 214/816 (26%) | 1.73 (1.37, 2.17) | 3383 | 1.48 (1.19, 1.85) | 3383 |

Each outcome was analysed separately comparing differences in responses at each follow-up time point between arms using modified poisson regression with a difference-in-differences specification. The Relative Risk from the interaction term between intervention (vs control) and post (vs pre at Baseline) and 95% confidence intervals are reported. IPTW estimates are additionally weighted by stabilized inverse probability weights for meeting the per protocol definition of attending eight or more SKILLZ sessions to graduate. Standard errors are clustered at the school level.

## Table D in S1 Text

**Impact of SKILLZ on HIV testing and contraception among those recruited at baseline only**

| N=1918 | Intervention | Control | Intention To Treat | | IPTW | |
|---|---|---|---|---|---|---|
| **Outcomes** | **$n_1/N_1$# (%)** | **$n_2/N_2$# (%)** | **Relative Risk (95% CI) &** | **N** | **Relative Risk (95% CI) &** | **N** |
| Attended at least 1 session of SKILLZ | 802/941 (85%) | 0/977 (0%) | | | | |
| - Graduated from SKILLZ (8+ sessions attended) | 721/941 (76%) | 0/977 (0%) | | | | |
| Tested for HIV within the previous 6 months | | | | | | |
| - At 6-month follow-up | 495/828 (60%) | 287/791 (36%) | 1.66 (1.38, 2.00) | 1619 | 1.72 (1.43, 2.06) | 1619 |
| - At 12-month follow-up | 471/841 (56%) | 341/882 (39%) | 1.46 (1.22, 1.76) | 1723 | 1.55 (1.28, 1.86) | 1723 |
| Used contraception within the previous 6 months | | | | | | |
| - At 6-month follow-up | 212/619 (34%) | 188/633 (30%) | 1.19 (0.93, 1.53) | 1252 | 1.33 (1.04, 1.70) | 1252 |
| - At 12-month follow-up | 241/721 (33%) | 203/782 (26%) | 1.32 (1.07, 1.64) | 1503 | 1.43 (1.16, 1.76) | 1503 |

#Counts and proportions with the outcome, out of the total participants in each intervention arm that responded to each outcome variable.

&Each outcome was analysed separately comparing differences in responses at each follow-up time point between arms using modified poisson regression. The denominator is restricted to those who were recruited at Baseline only. The Relative Risk and 95% confidence intervals are reported. All models adjust for SHREYA empowerment score and employed/earns income due to chance imbalances at baseline. IPTW estimates are additionally weighted by stabilized inverse probability weights for meeting the per protocol definition of attending eight or more SKILLZ sessions to graduate. Standard errors are clustered at the school level.

## Table E in S1 Text

**Sensitivity analysis of primary outcomes by non-response bias**

| | Intention To Treat | | | | IPTW | | | |
|---|---|---|---|---|---|---|---|---|
| | Impute with zeros | | Impute with ones | | Impute with zeros | | Impute with ones | |
| **Outcomes** | **Relative Risk (95% CI) [&]** | **N** | **Relative Risk (95% CI) [&]** | **N** | **Relative Risk (95% CI) [&]** | **N** | **Relative Risk (95% CI) [&]** | **N** |
| Tested for HIV within the previous 6 months | | | | | | | | |
| - At 6-month follow-up | 1.60 (1.34, 1.91) | 2153 | 1.40 (1.24, 1.59) | 2153 | 1.65 (1.39, 1.96) | 2153 | 1.43 (1.27, 1.61) | 2153 |
| - At 12-month follow-up | 1.41 (1.17, 1.70) | 2153 | 1.31 (1.13, 1.53) | 2153 | 1.49 (1.23, 1.80) | 2153 | 1.37 (1.18, 1.59) | 2153 |
| Used contraception within the previous 6 months | | | | | | | | |
| - At 6-month follow-up | 1.18 (0.92, 1.51) | 2153 | 1.06 (0.97, 1.17) | 2153 | 1.31 (1.03, 1.66) | 2153 | 1.11 (1.01, 1.22) | 2153 |
| - At 12-month follow-up | 1.33 (1.07, 1.64) | 2153 | 1.15 (1.03, 1.29) | 2153 | 1.43 (1.16, 1.76) | 2153 | 1.20 (1.07, 1.34) | 2153 |

[&]Each outcome was analysed separately comparing differences in responses at each follow-up time point between arms using modified poisson regression. The Relative Risk from and 95% confidence intervals are reported. All models adjust for SHREYA empowerment score and employed/earns income due to chance imbalances at baseline and an indicator for whether each outcome was imputed. IPTW estimates are additionally weighted by stabilized inverse probability weights for meeting the per protocol definition of attending eight or more SKILLZ sessions to graduate. Standard errors are clustered at the school level.

## Table F in S1 Text

**Descriptive statistics comparing responders to non-responders for primary outcomes**

| 6-month follow-up | | | | | | | | | | | | | | | | |
|---|---|---|---|---|---|---|---|---|---|---|---|---|---|---|---|---|
| | Responded to HIV testing (N=1,845) | | | Did not respond to HIV testing (N=28) | | | Difference in means | P-value | Responded to contraception (N=1,426) | | | Did not respond to contraception (N=447) | | | Difference in means | P-value |
| **Characteristics** | **Mean** | **SD** | **N** | **Mean** | **SD** | **N** | | | **Mean** | **SD** | **N** | **Mean** | **SD** | **N** | | |
| Age | 17.7 | 1.3 | 1845 | 18.0 | 1.2 | 28 | -0.3 | 0.233 | 17.8 | 1.3 | 1426 | 17.6 | 1.5 | 447 | 0.1 | 0.092 |
| HIV Knowledge (Correct out of 7) | 5.51 | 1.17 | 1845 | 4.50 | 1.77 | 28 | 1.006 | 0.003** | 5.53 | 1.15 | 1426 | 5.37 | 1.29 | 447 | 0.154 | 0.044* |
| Total number of sexual partners | 0.62 | 1.96 | 1843 | 0.18 | 0.48 | 28 | 0.438 | 0.000*** | 0.73 | 2.10 | 1424 | 0.24 | 1.28 | 447 | 0.482 | 0.000*** |
| SHREYA empowerment score (/ 105) | 83 | 17 | 1845 | 49 | 27 | 28 | 34 | 0.000*** | 83 | 17 | 1426 | 78 | 21 | 447 | 5 | 0.000*** |
| | **n** | **%** | **N** | **n** | **%** | **N** | | | **n** | **%** | **N** | **n** | **%** | **N** | | |
| Employed/earns income | 534 | 29% | 1835 | 8 | 30% | 27 | -0.005 | 0.952 | 429 | 30% | 1420 | 113 | 26% | 442 | 0.047 | 0.049* |
| Experienced food insecurity in the previous month | 541 | 30% | 1820 | 8 | 33% | 24 | -0.036 | 0.734 | 457 | 32% | 1413 | 92 | 21% | 431 | 0.110 | 0.000*** |
| Ever had sex | 560 | 31% | 1812 | 2 | 8% | 25 | 0.229 | 0.005** | 503 | 36% | 1414 | 59 | 14% | 423 | 0.216 | 0.000*** |
| Received money/support from sexual partner (within the previous 6 months) | 523 | 46% | 1141 | 6 | 55% | 11 | -0.087 | 0.508 | 477 | 50% | 959 | 52 | 27% | 193 | 0.228 | 0.000*** |
| Ever pregnant | 36 | 2% | 1834 | 0 | 0% | 25 | 0.020 | 0.000*** | 34 | 2% | 1423 | 2 | 0% | 436 | 0.019 | 0.001*** |
| Tested for pregnancy | 208 | 11% | 1834 | 3 | 13% | 24 | -0.012 | 0.841 | 190 | 13% | 1423 | 21 | 5% | 435 | 0.085 | 0.000*** |
| Friend ever pregnant | 1166 | 70% | 1660 | 13 | 68% | 19 | 0.018 | 0.862 | 920 | 71% | 1297 | 259 | 68% | 382 | 0.031 | 0.231 |
| - Friend ever abortion | 427 | 48% | 895 | 5 | 45% | 11 | 0.023 | 0.905 | 346 | 47% | 729 | 86 | 49% | 177 | -0.011 | 0.758 |
| Ever STI symptoms | 211 | 11% | 1845 | 2 | 7% | 28 | 0.043 | 0.371 | 183 | 13% | 1426 | 30 | 7% | 447 | 0.061 | 0.000*** |
| **12-month follow-up** | | | | | | | | | | | | | | | | |
| | Responded to HIV testing (N=1,939) | | | Did not respond to HIV testing (N=58) | | | Difference in means | P-value | Responded to contraception (N=1,675) | | | Did not respond to contraception (N=322) | | | Difference in means | P-value |

| Characteristics | Mean | SD | N | Mean | SD | N | | | Mean | SD | N | Mean | SD | N | | |
|---|---|---|---|---|---|---|---|---|---|---|---|---|---|---|---|---|
| Age | 18.3 | 1.4 | 1939 | 18.4 | 1.1 | 58 | -0.1 | 0.729 | 18.4 | 1.4 | 1675 | 18.2 | 1.2 | 322 | 0.2 | 0.012* |
| HIV Knowledge (Correct out of 7) | 5.68 | 1.15 | 1939 | 4.76 | 1.60 | 58 | 0.919 | 0.001*** | 5.68 | 1.16 | 1675 | 5.52 | 1.29 | 322 | 0.161 | 0.023* |
| Total number of sexual partners | 0.70 | 1.26 | 1937 | 0.61 | 1.31 | 57 | 0.083 | 0.542 | 0.78 | 1.31 | 1673 | 0.26 | 0.83 | 321 | 0.512 | 0.000*** |
| SHREYA empowerment score (/ 105) | 84 | 18 | 1939 | 48 | 33 | 58 | 36 | 0.000*** | 84 | 18 | 1675 | 76 | 25 | 322 | 9 | 0.000*** |
| | **n** | **%** | **N** | **n** | **%** | **N** | | | **n** | **%** | **N** | **n** | **%** | **N** | | |
| Employed/earns income | 522 | 27% | 1934 | 11 | 21% | 52 | 0.058 | 0.363 | 464 | 28% | 1671 | 69 | 22% | 315 | 0.059 | 0.022* |
| Experienced food insecurity in the previous month | 519 | 27% | 1922 | 10 | 20% | 50 | 0.070 | 0.304 | 457 | 27% | 1665 | 72 | 23% | 307 | 0.040 | 0.143 |
| Ever had sex | 704 | 37% | 1912 | 9 | 23% | 39 | 0.137 | 0.050* | 670 | 41% | 1650 | 43 | 14% | 301 | 0.263 | 0.000*** |
| Received money/support from sexual partner (within the previous 6 months) | 641 | 50% | 1270 | 7 | 37% | 19 | 0.136 | 0.352 | 605 | 52% | 1170 | 43 | 36% | 119 | 0.156 | 0.006** |
| Ever pregnant | 65 | 3% | 1932 | 2 | 5% | 42 | -0.014 | 0.677 | 65 | 4% | 1672 | 2 | 1% | 302 | 0.032 | 0.000*** |
| Tested for pregnancy | 243 | 13% | 1925 | 3 | 8% | 38 | 0.047 | 0.275 | 236 | 14% | 1665 | 10 | 3% | 298 | 0.108 | 0.000*** |
| Friend ever pregnant | 1177 | 69% | 1705 | 15 | 48% | 31 | 0.206 | 0.012* | 1030 | 69% | 1486 | 162 | 65% | 250 | 0.045 | 0.187 |
| - Friend ever abortion | 412 | 47% | 869 | 5 | 56% | 9 | -0.081 | 0.640 | 370 | 47% | 785 | 47 | 51% | 93 | -0.034 | 0.504 |
| Ever STI symptoms | 230 | 12% | 1939 | 8 | 14% | 58 | -0.019 | 0.545 | 218 | 13% | 1675 | 20 | 6% | 322 | 0.068 | 0.000*** |

* p<0.05, ** p<0.01, *** p<0.001. P-values were generated from linear regressions of each variable on an indicator variable for whether the outcome variable is missing, controlling for treatment assignment and clustering standard errors at the school level.

## Table G in S1 Text

**Sensitivity analysis of primary outcomes to timing of enrolment**

| | Controlling for indicator variable for additional enrolment | | | | Controlling for month of recruitment fixed effects | | | |
|---|---|---|---|---|---|---|---|---|
| | Intention To Treat | | IPTW | | Intention To Treat | | IPTW | |
| **Outcomes** | **Relative Risk (95% CI) &** | **N** | **Relative Risk (95% CI) &** | **N** | **Relative Risk (95% CI) &** | **N** | **Relative Risk (95% CI) &** | **N** |
| Tested for HIV within the previous 6 months | | | | | | | | |
| - At 6-month follow-up | 1.60 (1.34, 1.91) | 1845 | 1.65 (1.39, 1.96) | 1845 | 1.70 (1.43, 2.02) | 1845 | 1.72 (1.45, 2.03) | 1845 |
| - At 12-month follow-up | 1.41 (1.17, 1.70) | 1939 | 1.49 (1.23, 1.80) | 1939 | 1.48 (1.20, 1.81) | 1939 | 1.55 (1.27, 1.91) | 1939 |
| Used contraception within the previous 6 months | | | | | | | | |
| - At 6-month follow-up | 1.18 (0.93, 1.51) | 1426 | 1.31 (1.03, 1.67) | 1426 | 1.28 (0.99, 1.65) | 1426 | 1.43 (1.11, 1.84) | 1426 |
| - At 12-month follow-up | 1.32 (1.07, 1.64) | 1675 | 1.42 (1.16, 1.75) | 1675 | 1.40 (1.07, 1.83) | 1675 | 1.47 (1.13, 1.92) | 1675 |

[#]Counts and proportions with the outcome, out of the total participants in each intervention arm that responded to each outcome variable.

[&]Each outcome was analysed separately comparing differences in responses at each follow-up time point between arms using modified poisson regression. The Relative Risk from and 95% confidence intervals are reported. All models adjust for SHREYA empowerment score and employed/earns income due to chance imbalances at baseline. IPTW estimates are additionally weighted by stabilized inverse probability weights for meeting the per protocol definition of attending eight or more SKILLZ sessions to graduate. Standard errors are clustered at the school level.

## Table H in S1 Text

**HIV testing and contraception uptake at the Graduation Event**

| | Self-reported outcomes[1] | Services received at Graduation Event[2] |
|---|---|---|
| **Outcomes** | **$n_1/N_1$[#] (%)** | **$n_2/N_2$ (%)** |
| Tested for HIV within the previous 6 months | | |
| - At Baseline | 312/917 (34%) | - |
| - At 6-month follow-up | 600/1018 (59%) | 668/1134 (59%) |
| Used contraception within the previous 6 months | | |
| - At Baseline | 140/814 (17%) | - |
| - At 6-month follow-up | 281/765 (37%) | 114/1134 (10%) |

[#]Counts and proportions with the outcome, out of the total participants in the intervention arm that responded to each outcome variable.

[1] Survey data from participants in the intervention arm, self-reported.

[2] Aggregate administrative data from Grassroot Soccer, divided by the total number of participants in the intervention arm. Note that survey data asks respondents to report HIV testing and contraceptive use from any source, not restricted to services received at the graduation event.

## Table I in S1 Text

**HIV testing and contraception uptake at 6 vs 12 months**

| Tested for HIV within the previous 6 months | At 12 months | | | |
|---|---|---|---|---|
| **At 6 months** | **No** | **Yes** | **No Response** | **Total** |
| - No | 689 (74%) | 192 (20%) | 57 (6%) | 938 |
| - Yes | 239 (26%) | 603 (66%) | 65 (7%) | 907 |
| - No response | 98 (32%) | 118 (38%) | 92 (30%) | 308 |
| Total | 1026 (48%) | 913 (42%) | 214 (9%) | 2153 |

| Used contraception within the previous 6 months | At 12 months | | | |
|---|---|---|---|---|
| **At 6 months** | **No** | **Yes** | **No Response** | **Total** |
| - No | 684 (72%) | 138 (15%) | 123 (13%) | 945 |
| - Yes | 162 (34%) | 243 (51%) | 76 (16%) | 481 |
| - No response | 318 (44%) | 130 (18%) | 279 (38%) | 727 |
| Total | 1164 (54%) | 511 (24%) | 478 (22%) | 2153 |

## Figure J in S1 Text

**Timeline of Data Collection and Implementation of SKILLZ at each school**

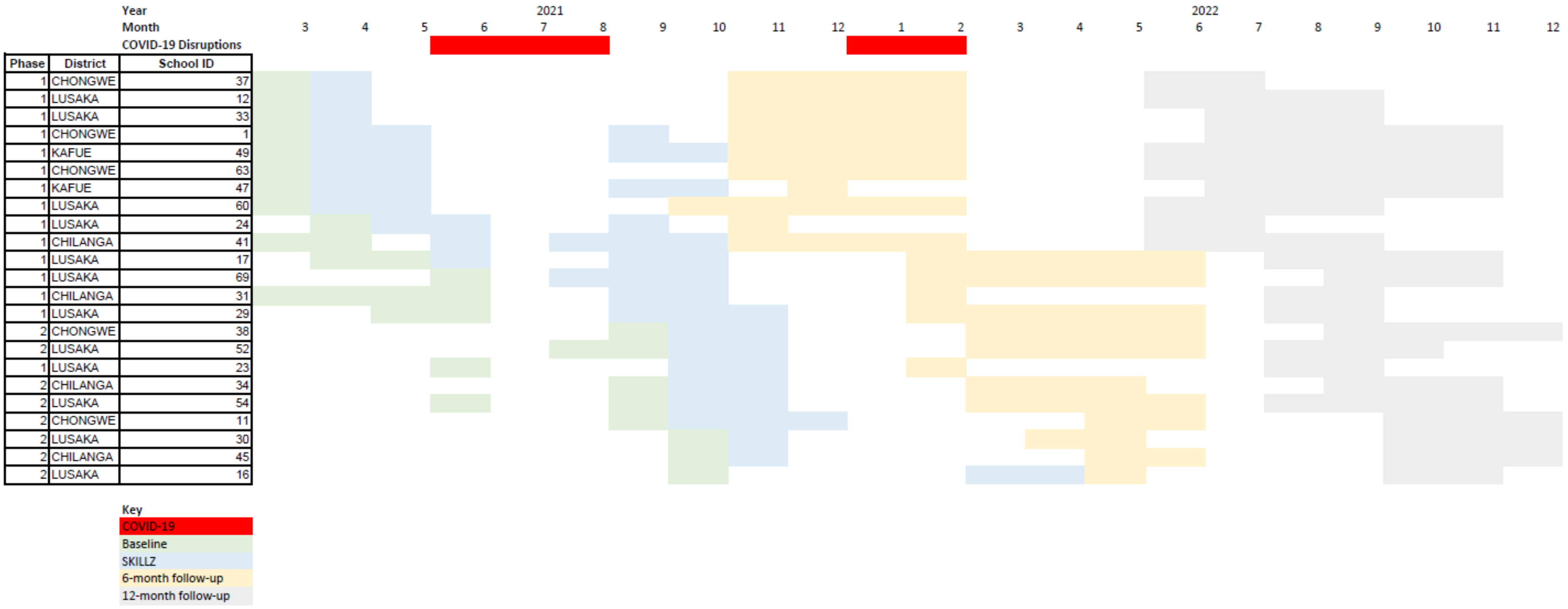


All study procedures occurred on a rolling basis at each school between March 9, 2021, and January 18, 2023. Dates of data collection activities up to the 90th percentile are plotted. Due to the COVID-19 pandemic, study activities were paused between June 16 and August 25, 2021, and again from January 10 to February 4, 2022. The follow-up surveys were designed to be conducted 6- and 12-months after Baseline respectively, and all SKILLZ activities completed before the 6-month survey. In practice, follow-up surveys were delayed due to COVID-19 induced disruptions to different degrees by school – 90% of 6-month (12-month) follow-up surveys occurred within 9-months (15-months) of Baseline– which also resulted in differences in time since SKILLZ completion among respondents in the intervention arm.

Note: implementation at School ID 23 was delayed until Phase 2 because the school instructed Grassroot Soccer not to begin SKILLZ until the third term of the school year instead of the second term as originally planned.

## Figure K in S1 Text

**Geographical map of participating schools in Lusaka by treatment assignment**

**Treatment Arm**

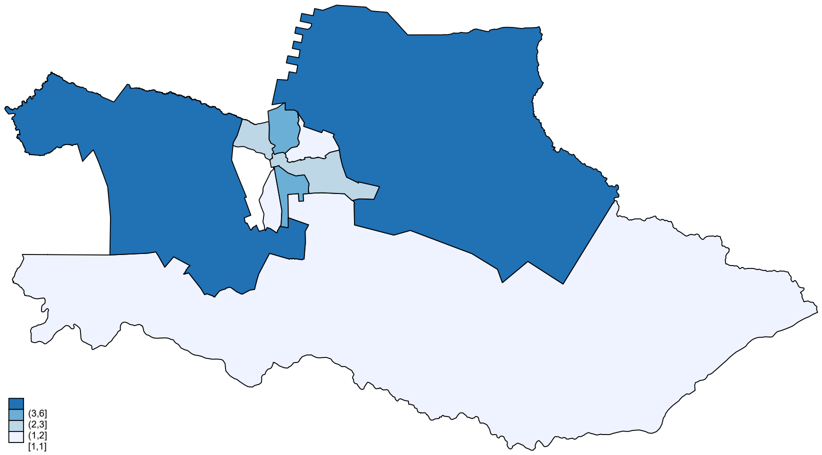


**Control Arm**

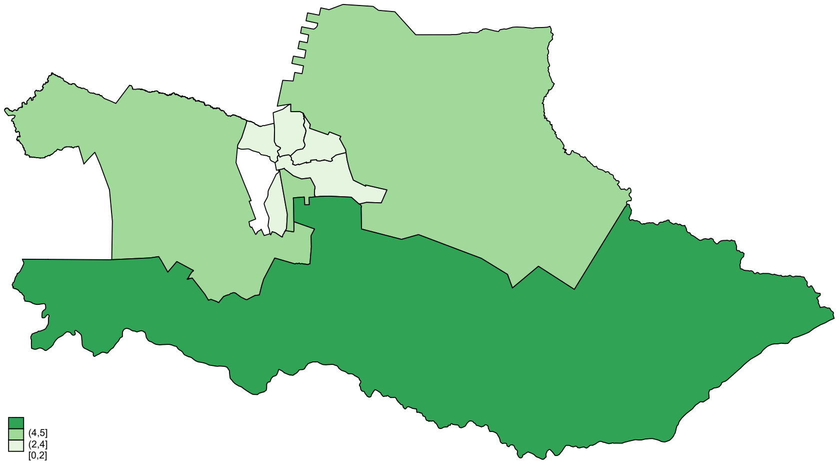


Number of schools in the Treatment/Control arms respectively aggregated at the ward level based on 2020 Constituency Boundaries. The base layer of the map is provided by OCHA Regional Office for Southern and Eastern Africa (ROSEA) via DMMU Zambia and hosted by The Humanitarian Data Exchange (https://data.humdata.org/dataset/zambia_adm_boundaries).

## Figure L in S1 Text

**Heterogenous treatment effects by school**

*1a: Entire Sample*

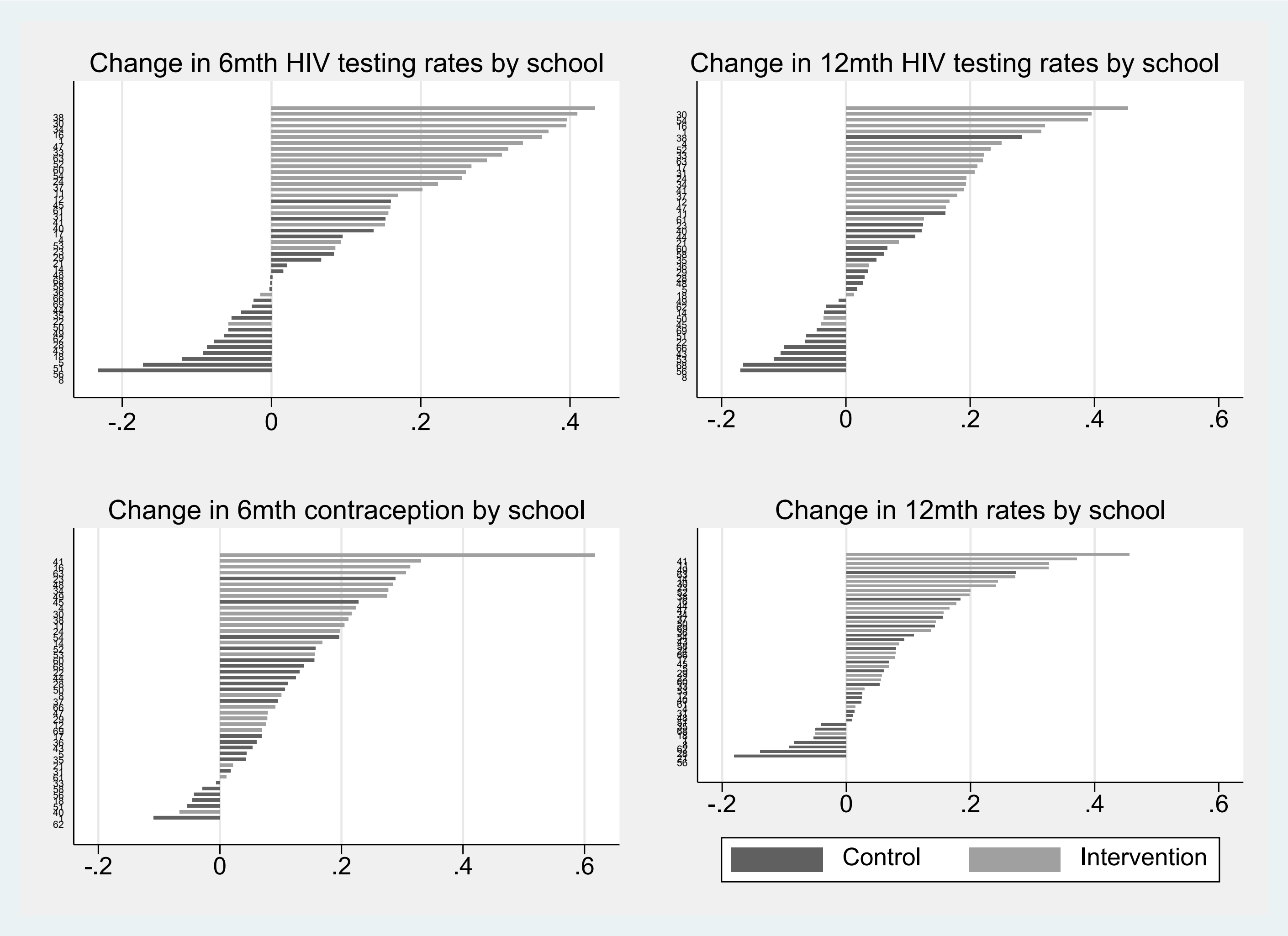


*1b: restricting to balanced sample of those with both baseline and follow-up measure*

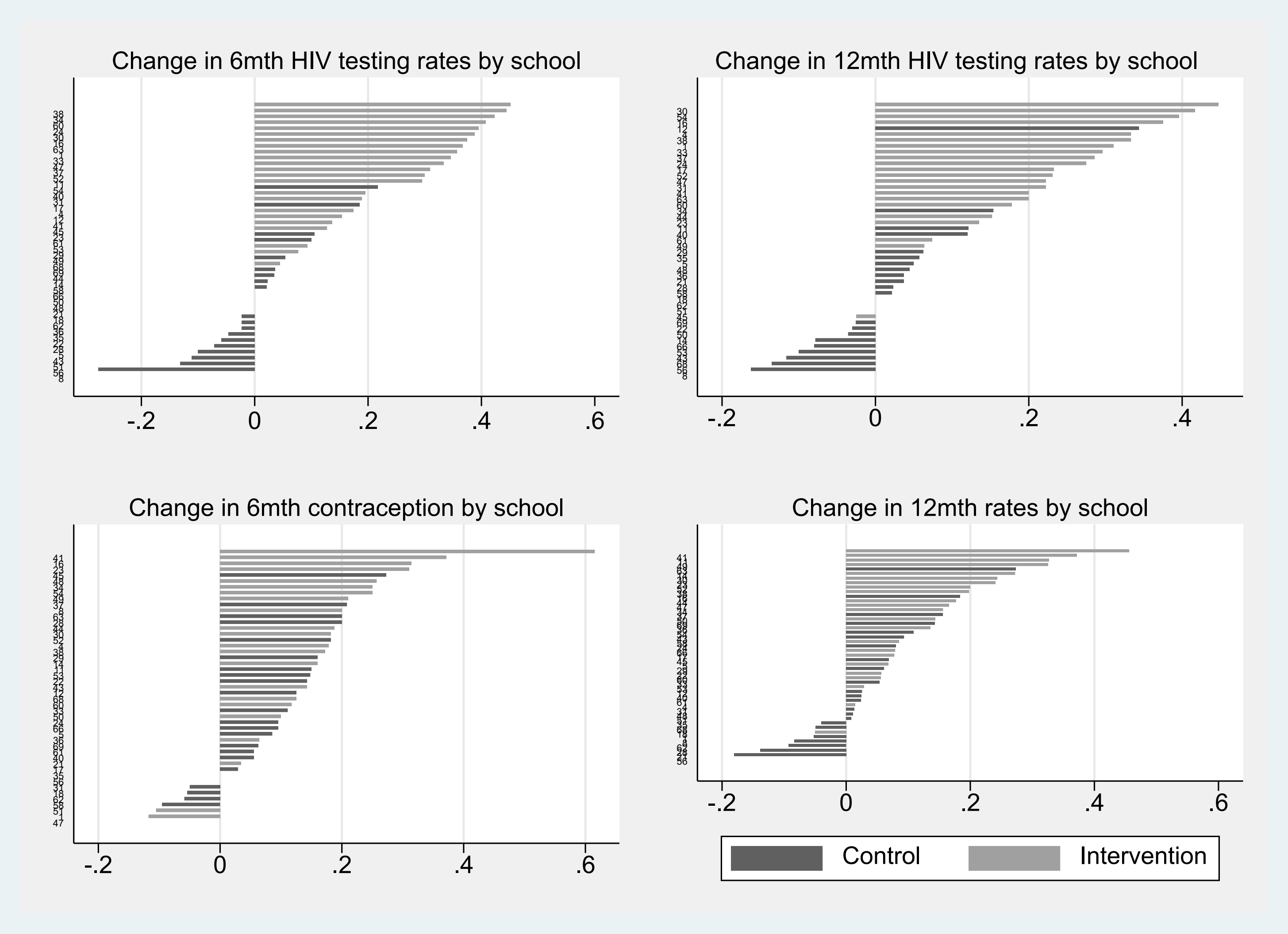


Differences in levels of HIV testing and contraception between 6- and 12-month follow-up and at baseline by school (labelled by School ID, Intervention arm indicated by shading). Differences were calculated by subtracting baseline rates from 6/12-month rates. Positive difference indicates increase in outcome over time. Panel 1a presents differences for the entire study sample; 1b presents differences restricted to a balanced sample of those with both baseline and follow-up measures.

## Box M in S1 Text

### Additional detail on economic evaluation

**Methods**

We completed a full costing and cost-effectiveness analysis of the two study arms from the health-care provider perspective.

Resource use data were collected by use of an ingredients-based approach, in which each resource required for the intervention was identified and valued. A detailed costing tool was developed that aimed to describe resource items grouped into five categories: staff costs (e.g., salary, employee benefits, professional development), administrative costs (e.g., building rent, utilities, office equipment/supplies), travel (e.g., vehicle purchase, local transport, air travel), equipment/maintenance (e.g., soccer goals, balls, jerseys, coach kits, family planning and HIV testing commodities), and others (e.g., coach training, meals, minor unanticipated expenses). Costs were compiled in quarterly increments. This approach also allowed us to categorize costs as start-up costs (e.g., office equipment, vehicle purchase), capital costs (e.g., coach training, soccer equipment), and recurrent costs (e.g., salaries, rents, utilities, travel, equipment maintenance, and meals).

Costs were mainly collated from a combination of GRS and CIDRZ financial and utilisation documents and invoices provided to the trial administration. Family planning and HIV testing commodities were procured centrally and distributed by the trial management group. The average cost of contraception was calculated using the distribution of methods used (e.g., oral contraceptive pill, IUD) in both study arms and their cost for approximately 6 months of contraceptive cover. The average cost of HIV testing was calculated using the overall distribution of self-testing and facility-based testing and the estimated cost for each method. All costs were inflated to 2023 $US.

The average total service cost per individual participant was calculated for each of the two study groups, as well as the difference in material costs. The difference in costs between study groups was compared against the differences in HIV testing uptake and proportion of girls using contraception to generate our incremental cost-effectiveness ratios (ICERs). Therefore, our ICERs were defined as i) the incremental cost per additional girl receiving an HIV test in the previous 6 months (based on 12-month results), and ii) the incremental cost per additional girl using contraception in the previous 6 months (based on 12 month results).

Sensitivity analyses were conducted by varying our effectiveness estimates based on the 95%CI of our main 12-month ITT analyses (i.e., Coefficient = 1.596 (95%CI 1.312-1.879) for HIV testing; Coefficient = 1.758 (95%CI 1.350-2.165) for contraception). Costs were not varied.

Data were compiled using Excel version 16.1 (Microsoft Software, Redmond, Washington, USA) and analysed using TreeAge Pro 2021 Version R2.1 (TreeAge Software, Williamstown, Massachusetts, USA).

**Results**

Differences in resource use were driven by the cost of delivering the GRS intervention and differences in HIV testing and contraception uptake between the two study arms. The overall cost of delivering the GRS intervention was $534,255. This was comprised of 51% staff costs, 3% administration, 15% travel, 12% equipment/maintenance, and 19% other costs. Alternatively, 18% start-up costs, 3% capital costs, and 79% recurrent costs (51% of recurrent costs were staff costs). Eighteen percent of programme costs were accumulated in the preparation phase of the intervention, 22% in the pilot phase, and 60% in the main phase.

The average cost per participant in the intervention group was $472, and the average cost per participant in the control group was $6. Comparing these values against our adjusted 12-month effectiveness results gave estimated ICERs of $2,006 per additional girl receiving an HIV test, and $2,362 per additional girl using contraception. In sensitivity analysis, our HIV testing ICER varied between $1,358-$3,811 per additional girl receiving an HIV test when we varied effectiveness based on our ITT 95%CI. Our family planning ICER varied between $1,542-$5,091 per additional girl using contraception when we varied effectiveness based on our ITT 95%CI.

[35][36]

**a)**

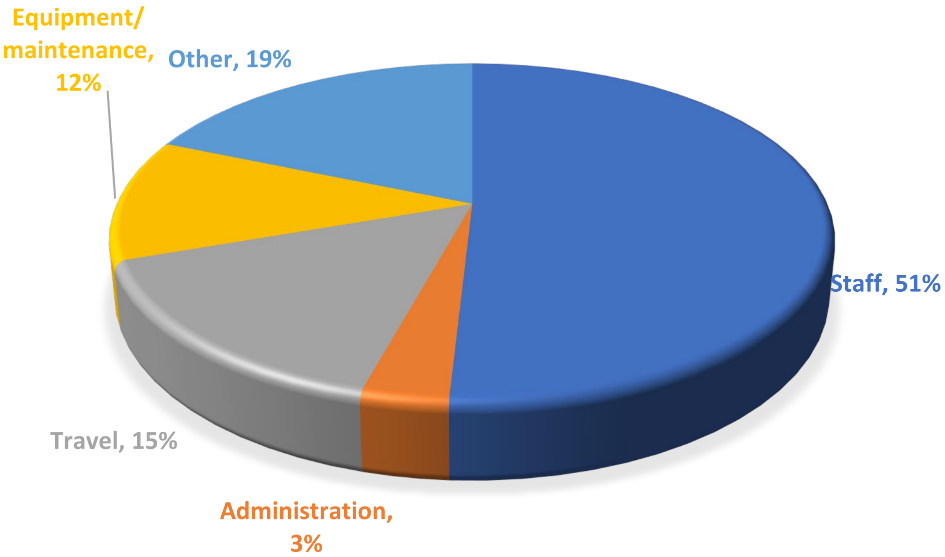
Equipment/
maintenance,
12%
Other, 19%
Staff, 51%
Travel, 15%
Administration,
3%

b)

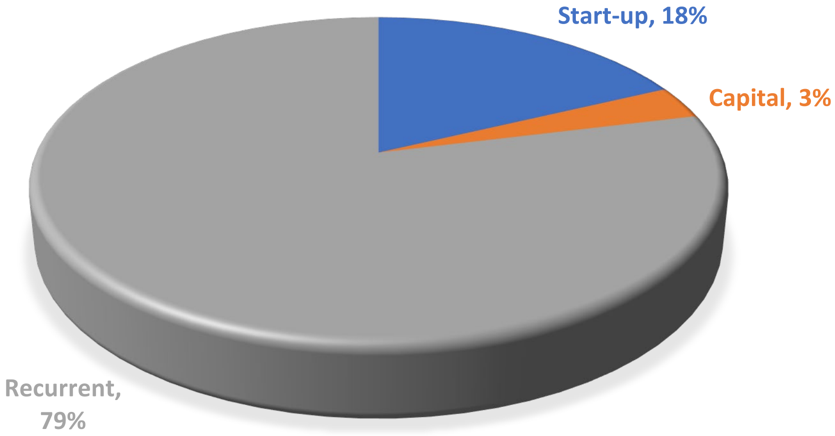
Start-up, 18%
Capital, 3%
Recurrent,
79%
