# Supplementary material for: Evaluation of the SKILLZ intervention to promote HIV testing and contraception uptake in adolescent girls in Lusaka, Zambia: A cluster-randomized trial
Source: PLOS Glob Public Health. 2025 Oct 29;5(10):e0005375. doi: 10.1371/journal.pgph.0005375 (PMC12571267; doi:10.1371/journal.pgph.0005375)
Supplement: S2 Text — (PDF) [file pgph.0005375.s002.pdf]

Study Title: Reaching 90 90 90 in Adolescents in Zambia: Using all our SKILLZ

Version 1.7

**Short title:**	SKILLZ Study
**Protocol version:**	1.7 dated 12th March2021
**Funder:**	U.S. National Institutes of Health
**Principal Investigator**:	Dr Carolyn Bolton Moore
**Co-Principal Investigator:**	Dr Nancy Padian

Table of Contents

1. Introduction 4
a. General information on the research issue/topic 4
b. Overview of research and research gaps (contribution to policy and practice) 4
c. Overall purpose of the research and area/site where the research will be conducted 5
2. Statement of the problem 5
a. Short summary of the background on the problem 5
b. Importance of relevance of the research 6
3. Rationale/Justification 6
a. Contribution of the study to science or body of knowledge 6
b. Changes to be made by the study 7
c. Evidence supporting the justification 7
4. Theoretical/conceptual framework 8
5. Literature review 14
6. Research questions 16
7. Research aim(s)/General Objective and Specific Objectives 16
8. Methodology 17
a. Study design 17
b. Study site and population/Research materials 18
c. Selection of participants, sampling methods and sample size 19
d. Data collection plan and tools 21
e. Data management and storage 31
f. Data analysis plan 32
9. Ethical considerations 34
10. Timelines 36
11. Budget 37
12. Appendices 38
a. Letter of Permission from MOGE for GRS to continue working in schools in 2019 38
b. MOU between GRS and MOGE 39
c. Letter of Permission between MOH and GRS 47
13. References 52

Investigators and Collaborators

	Organization	Role on Project	FWA/SEV#	Funding
Carolyn Bolton Moore, MD, MSc Epi	CIDRZ	Principle Investigator	FWA00005960	See below
Nancy Padian, PHD, MPH	University of California, Berkeley	Co-Principle Investigator	FWA00006252	
Jenny Liu, PHD, MPP	University of California, San Francisco	Co-Investigator, Scientific Lead	FWA00000068	
Jake Pry, MPH, PhD	Washington University	Epidemiologist	FWA00000338	
Jenala Chipungu, MPH	CIDRZ	Qualitative Analyst	FWA00000338	
Helene Smith, MPH	CIDRZ	Implementation Scientist	FWA00000338	
Taniya Tembo, MSc	CIDRZ	Costing Research Associate	FWA00000338	
Boyd Mkandawire, MBA & MPH	GRS	Co-Investigator	FWA002750	
Jeff DeCelles, EdM & DrPH	GRS	Co-Investigator	FWA002750	
Chelsea Coakley, MPhil	GRS	Co-Investigator	FWA002750	

Funding Information

This work is supported by the US National Institute of Health via Grant Number 1R01MH116789-01A1

1. Name of Funding Agency: US National Institute of Health (NIH)
2. Principal Investigators: Dr Carolyn Bolton Moore, MD. MSc & Dr Nancy Padian, PHD, MPH
3. FAIN #: R01MH116789
4. Title of Proposal: Reaching 90 90 90 in Adolescents in Zambia: Using all our SKILLZ (SKILLZ)
5. Approval period: 25-AUGUST-2018 – 31-MAY-2023

Ethical reviews by other institutions

The protocol has been submitted for review to the Institutional Review Boards (IRBs) of the University of Zambia and the University of Alabama—Birmingham.

Funder's Role

The NIH's role is non-engaged and will not include access to identifiable data. The NIH solely will provide funding for the activity.

Acronyms

**AFS**	Adolescent Friendly Services
**ART**	Antiretroviral Therapy
**ARVs**	Antiretrovirals
**CIDRZ**	Centre for Infectious Disease Research in Zambia
**CRF**	Case Reporting Forms
**DHS**	Demographic Health Survey
**DMPA-SC**	Depo Medroxyprogesterone Acetate
**EMR**	Electronic Management Records
**FGD**	Focus Group Discussion
**GRS**	Grassroot Soccer
**HIV**	Human Immunodeficiency Virus
**HIVST**	HIV Self-Testing
**HTC**	HIV Testing & Counselling
**ICER**	Incremental Cost-Effectiveness Ratio
**IDI**	In-Depth Interview
**LIMS**	Laboratory Information Management System
**MOU**	Memorandum of Understanding
**MOGE**	Ministry of General Education
**MoH**	Ministry of Health
**NHC**	Neighborhood Health Committee
**NIH**	National Institute of Health
**QALY**	Quality-Adjusted Life Years
**ROI**	Return on Investment
**SBHP**	Sports-based HIV prevention programs
**SOC**	Standard of Care
**SRH**	Sexual Reproductive Health
**STI**	Sexually Transmitted Infection
**UAB**	University of Alabama
**VLS**	Viral Load Suppression
**YFHS**	Youth Friendly Health Services
**ZAMPHIA**	Zambia population-based HIV impact assessment

1. Introduction

1. General information on the research issue/topic

Zambia is struggling to meet the 95-95-95 targets for adolescent women. Despite available public health services, including free HIV testing, prevention, care and treatment, uptake of most health services amongst adolescents in Zambia remains suboptimal, especially for sexual and reproductive health (SRH).[1,2] HIV testing rates remain low; only 47.5% of girls and 28.5% of boys aged 15-19 years have received an HIV test and learned their results, compared to 86.6% of adult women and 69.1% of men.[3] Preliminary results from the 2015-2016 Zambia population-based HIV impact assessment (ZAMPHIA) study show that adolescent girls and young women aged 15-24 years have higher rates of HIV infection compared to their male counterparts and that HIV prevalence among 20-24 year-olds is four times higher among females (8.6%) than males (2.1%).[4] In addition, whereas over 70% of HIV-infected adults have achieved viral load suppression (VLS), only 34% of adolescent girls have. To further support 95-95-95 targets, contraception is considered the most cost-effective strategy to reduce the burden of mother-to-child HIV transmission among women living with HIV who wish to prevent unintended pregnancy.[5] The most recent Demographic Health Survey (DHS) showed that while 73.3% of unmarried, sexually active adolescent girls reported not wanting a child in the next two years, only 20.6% were using any contraceptive method to prevent pregnancy.[3] Further to this, approximately 7.2% of sexually active adolescents (aged 15-19) reported having had a sexual partner who was 10 or more years older[3], placing them at an increased risk of HIV infection and early child-bearing[6] and the Ministry Of General Education (MOGE) has recorded a five-fold increase in teenage pregnancies among girls in grades 1-12 between 2002 and 2015[7]. The Government of Zambia has recognized that this data clearly points to the need to increase adolescents' awareness and utilization of available health services and ensure innovation in the way these messages are delivered.

2. Overview of research and research gaps (contribution to policy and practice)

In a review of adolescent-specific service delivery programs over 2001-2014,[8] only 11 studies were identified that addressed the HIV treatment cascade among adolescents, none of which met criteria for a rigorous, adequately powered, longitudinal program. Although the evidence base is thus weak, programs that are easily accessible, and provide integrated services and peer counselling were highlighted as promising strategies. Recent research from the Southern African region has shown that effective programs for suppressing viral load in adolescents focus on quality of clinical services, including adequate drug inventory, dedicated staff time for adolescents, staff who are kind and non-judgmental, funded mechanisms for adolescent clinical accompaniment.[9] Little research has been done as to what effect these programs could have on uptake of contraceptive services and whether a more holistic approach with a more integrated program (encompassing contraception, distribution of commodities, and the availability of HIV self-testing kits) could provide improved HIV-testing uptake as well as uptake of other contraceptive services.

We therefore seek to evaluate an enhanced school-based intervention for girls aged 16 and over that combines the innovative demand-generating sports-based program for empowering adolescent girls, supported by Grassroot Soccer (GRS) combined with adolescent-friendly clinical services (SKILLZ-girl and enhanced SKILLZ-girl) delivered through a combination of community and facility-based approaches. The SKILLZ Package

removes barriers to services across multiple levels of influence by creating a fun, interactive environment for meaningful discussions and reflection about SRH, future orientation, and goal setting (see **Figure 1**) using a school-based curriculum, peer mentorship, community soccer events and enhanced events, and referrals for and direct service provision. The program also provides opportunity for ongoing engagement with all enrolled girls (regardless of HIV status) through the form of school-based clubs that provide on-going discussion around positive messages of empowerment, strategies to prevent STI infection (including HIV), and direct linkages to access for contraceptives and repeat HIV testing. A rigorous, mixed methods evaluation will assess the effectiveness of this intervention following a cohort of enrolled girls over two years.

The findings from our research will be shared with both the Ministry of Health (MoH) and MOGE and will guide Zambia's national strategic plan for improved uptake and retention of SRH services by adolescents. We are working closely with these partners and Lusaka District Education Board Secretary to pave the way for complete integration of the SKILLZ Package into the public education system should this intervention prove effective. Our team will work closely with the MOH and MOGE throughout study implementation to ensure that services are integrated, streamlined and sustainable as well providing guidance on sustainable forms of distribution at the community level.

3. Overall purpose of the research and area/site where the research will be conducted

As part of routine activities, GRS is already implementing an approved, school-based curriculum at the majority of secondary schools in Lusaka however, it is unclear as to whether this curriculum, in itself, will improve health seeking behaviours and ultimately decrease HIV infection and unintended pregnancies amongst this high-risk group.

The overall purpose of the research is to assess the impact of the enhanced SKILLZ GIRL Package, including different components of the program aimed at both HIV-infected and uninfected adolescent schoolgirls over two years on the outcomes of (1) HIV testing and subsequent retention in care, and (2) contraceptive uptake and continuation for pregnancy prevention. We will also assess the impact of school based SKILLZ clubs, on 1) HIV testing and subsequent retention in care, and (2) contraceptive uptake and continuation for pregnancy prevention. The study will take place across several high-density population areas of Lusaka where CIDRZ supports government MoH clinics with ARV services, electronic data management, and youth-friendly trained clinical personnel and where GRS has been implementing their basic SKILLZ curriculum and events in secondary schools.

4. Statement of the problem

1. Short summary of the background on the problem

As mentioned above, the ZAMPHIA study showed that adolescent girls and young women have very high, disproportionate rates of HIV infection with a prevalence among 20-24 year-olds four times higher than that seen in males.[4] According to DHS, girls in Zambia have a sexual debut at 13 years of age, highlighting the need for early educational and behavioural interventions. Empowering girls and giving them access to SRH and broader health related information remains a key strategy to reducing risk of HIV acquisition and encourages more positive health seeking behaviour as part of a comprehensive health care package. Whilst

many adolescent programs are based on the premise that understanding risk and providing adolescent-friendly services (AFS) translates into participation along all steps in the SRH care cascade, this is not always the case. AFS are necessary, but insufficient; furthermore, the link between risk and uptake of AFS is tenuous. Compared to individuals at other life stages, adolescents are more likely to engage in novelty-seeking and risky behaviours and less likely to consider the consequences as they transition to adulthood and assume more independence and responsibility.[10-15]

2. Importance of relevance of the research

Strong evidence for what works for improving SRH outcomes among adolescent in resource-poor, but high disease burden settings remains poor despite the plethora of studies and investments into SRH programming. For example, guidelines in a 2016 WHO review of recommendations for care of HIV-positive youth were based on "low" and "very low" quality evidence, strongly suggesting the need for greater research.[16] Similarly, in a recent report, the Institute of Medicine concluded that there is a lack of evidence for effective models of adolescent health care and a need for further research, particularly on how health services are delivered and by whom.[17] Although a systematic review of sports-based HIV prevention programs (SBHP) has found evidence for effects on a range of social and behavioural outcomes (i.e., reduced stigma, increased self-efficacy, HIV-related knowledge, uptake of HIV testing a counselling (HTC), and condom use)[18], rigorous evaluations that can attribute causality and verify pathways for change are lacking.[18,19] As mentioned previously, GRS has already been rolling out a validated SKILLZ girl curriculum. In collaboration with their team, we have developed an enhanced curriculum which includes a comprehensive module on HIV self-testing, contraceptive choices and pre-exposure prophylaxis (PrEP) combined with increased access to these services through an enhanced soccer event.

In this study, we are proposing a pragmatic trial (based on real-world implementation) that will provide a robust evaluation of effectiveness and cost-effectiveness (CE) between a standard SKILLZ- girl curriculum, that has been delivered in many Lusaka secondary schools already, and a more robust, integrated, enhanced approach that includes additional SRH service offerings, including HIVST, PrEP and an expanded contraceptive method mix.

3. Rationale/Justification

1. Contribution of the study to science or body of knowledge

Few prospective studies have been conducted in sub-Saharan Africa (SSA) among adolescent girls to gauge uptake of SRH services, including contraception uptake and continuation and HIV testing, adherence and retention in care. Lall et al. reviewed 26 studies on adherence to and retention in care among HIV-positive youth and adolescents, but none were conducted in SSA.[20] Another review examined data from nine studies in SSA, but all were cross sectional and only one used viral load to assess HIV treatment adherence (as opposed to self-report).[21] Specifically, in Botswana, Lowethal et al. reported that 77% of 692 youth were virally suppressed, but again this was cross-sectional.[22] Finally, a third review examined data from 53 countries and reported higher ART adherence rates among African adolescents 84% (95% CI 79–89; I2:93%) compared to other regions of the world, but this too used cross-sectional data.[23] Furthermore, no studies have examined contraceptive continuation among adolescents, despite the burden of early pregnancy among young girls during their critical years in the transition to adulthood.

2. Changes to be made by the study

In Zambia, SRH services for adolescents remains a challenge with services primarily being provided through main-stream, clinic-based services. Uptake of both prevention and curative services remain low. For example, adolescents disproportionately contribute to the number of new HIV infections each year[3]. Standard of Care (SOC) for SRH and HIV services designed specifically for adolescents is scant and includes provision of basic SRH/HIV education, including HTC in schools by trained health workers and referral for other prevention services to general (mainly adult) ART/ SRH clinics. Adolescent girls found to be HIV-infected are also referred to these clinics for treatment and other services, however adherence and retention in care is low with only 34% reported to be virologically suppressed[4]. Furthermore, access to contraceptive services including long-acting methods is largely limited to clinical settings where adolescents face stigma (perceived and actual) from healthcare workers and risk lapses in confidentiality[24,25].

Our central hypothesis is that an enhanced SRH curriculum (including a comprehensive module on HIVST, contraceptives and PrEP) and the additional offering of HIVST and contraceptive services at the event along with ongoing engagement the SKILLZ-Club program (Enhanced Arm) , will increase HIV testing and contraceptive uptake compared to the standard SKILLZ curriculum & standard event (SOC Arm). Furthermore, supporting subsequent ongoing education as well as linkage to services through SKILLZ school clubs in the enhanced arm will improve ongoing uptake of services and retention and will allow us the opportunity to measure the effect of the intervention longitudinally. We further hypothesize that our intervention will positively and directly affect a number of mediating factors: attendance to soccer events where community based SRH services are offered, SRH knowledge, empowerment, self-confidence, and perceptions of gender balance, and (reduced) stigma. For girls found to be HIV-positive, we further expect the follow-on SKILLZ intervention (SKILLZ-Plus) aimed at linkage to HIV care and treatment, will reduce HIV-related stigma, increase disclosure to family and partners, increase feelings of social support, empowerment, self-efficacy, and ultimately adherence to ARVs, viral load suppression (VLS) and retention in HIV care and treatment.

The SKILLZ program trains young adult mentors ("Coaches") as community change agents that deliver age-appropriate, gender-transformative curricula through a pedagogical approach that combines soccer metaphors and activities with accurate health information. The aim is to build the health and social assets of adolescents, facilitate access to health and social services, and support adherence to medical treatment and positive protective behaviours.

3. Evidence supporting the justification

Adherence to protective behaviours, treatment and psychosocial support for adolescents remains inconsistent. Care services are largely inefficient, ineffective and undifferentiated, lack psychosocial support elements, and rely on ineffective referral mechanisms between the schools and health facilities.[26] Our soccer-based SKILLZ Package has already demonstrated proof-of-concept, increasing knowledge about key risk factors (i.e., age disparate relationships and multiple partners) and changing harmful attitudes related to stigma and gender issues.[27] In 2010-2012, GRS also analysed the SKILLZ program compared to sub-regional DHS data, showing that among 11,000 youth (males and females) participants in Zambia, 62% tested for HIV, 100% learned their HIV test results, and 95% of those testing positive for HIV were linked to care.[28]

Accompanying qualitative research also revealed high program acceptance by parents, health providers and adolescents themselves, although the sentiment to have a girl-only program were salient. At that time, the program was linked to only one clinic, no formal evaluation was employed with a meaningful clinical outcome (i.e., viral load), and little focus on pregnancy prevention, contraceptive use, and more general SRH risks were measured.

4. Theoretical/conceptual framework for Grassroot Soccer SKILLZ Package

The SKILLZ Package is based on a multi-level socio-ecological conceptual framework of HIV-related behaviours and outcomes among adolescent girls (see **Figure 1a & b**).[29,30] Barriers to accessing services and adhering to risk reduction strategies through the continuum of care exist across all levels of influence: individuals may hold fears and misconceptions of HIV and suffer from stigma surrounding adolescent sexuality; fears and anxiety can lead to a lack of social support from partners and peer groups that would otherwise be protective; families and communities are often the source of perceived stigma, and further reinforce inequitable gender and social norms that amplify girls' vulnerability; health systems are often inaccessible and unfriendly to young people.[31-33] These myriad factors collide to prevent adolescent girls from reducing risk, accessing HIV and SRH services, and remain in care over the longer-term.

Figure 1a*. To improve HIV testing, linkage to treatment, and retention in care, SKILLZ targets multiple barriers to service utilization based on a socio-ecological approach to adolescent health.*

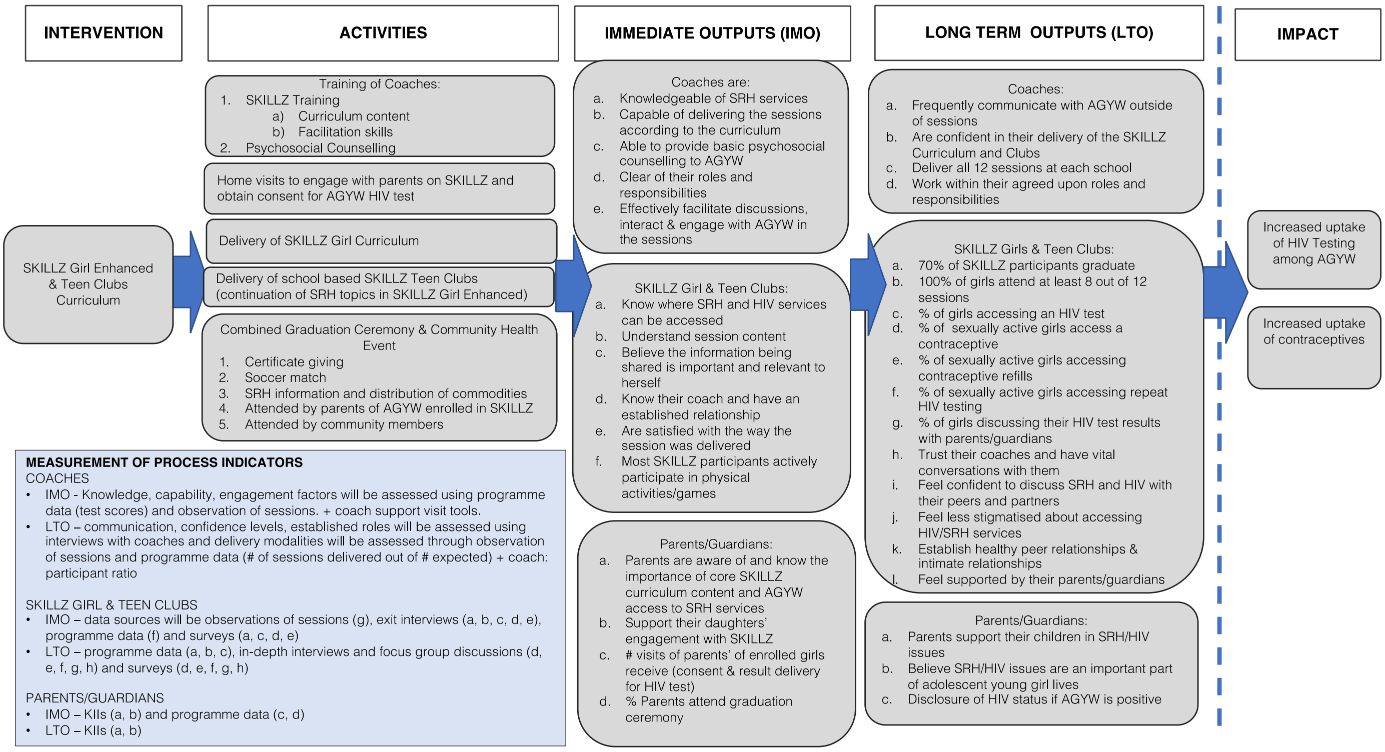

Figure 1b*.SKILLZ Community distribution of SRH commodities to improve uptake of SHR services (short term contraceptives and HIV testing) targets multiple barriers to service utilization based on a socio-ecological approach to adolescent health*

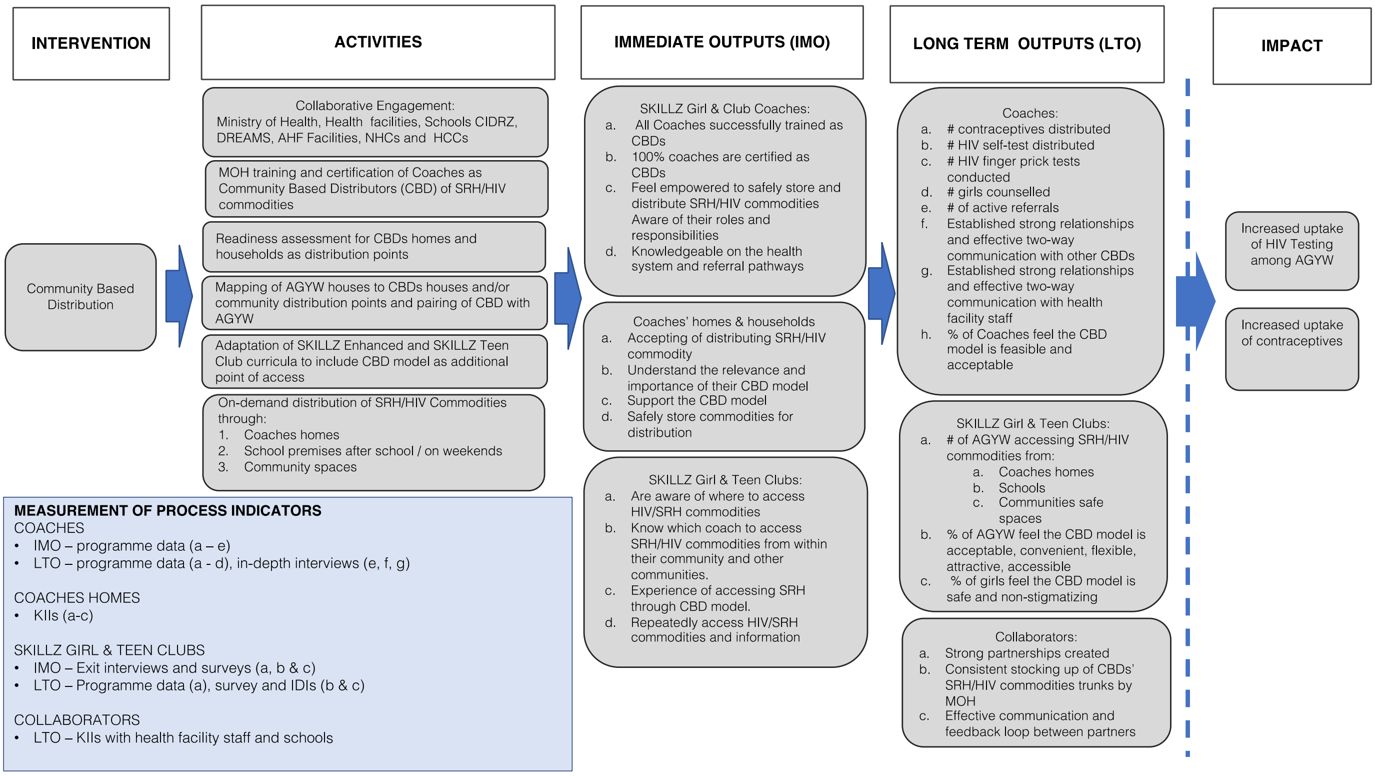

<u>The SKILLZ adolescent SRH program</u> ("SKILLZ Package") aims to improve uptake of HIV prevention and treatment services (i.e., testing, linkage to care, and treatment adherence), mental health and SRH services among adolescent girls. The model focuses on reducing barriers to access by creating a fun, safe, and interactive environment for meaningful discussions and reflection about health and well-being, including direct access to youth-friendly services and SRH commodities such as Condoms and Family Planning- pills and short term injectable up to 3 months (see **Figure 1** Theories of Change). The SKILLZ Package focuses on building adolescent girls' health and social assets, individual self-efficacy and self-concept, empowerment, and future orientation. It also aims to address detrimental social norms and attitudes towards girls and influence traditional service delivery models. Designed to be integrated into both secondary school education and community-based clinical services, the SKILLZ Package is unique in that it offers holistic and continuous support to adolescent girls through by:

1) Focusing on agency and empowerment by providing mentorship as well as accurate sexual health information with a trained near peer "Coach". Coaches are the foundation of SKILLZ and are young adult mentors that act as community change agents that deliver age-appropriate, gender-transformative curricula through a pedagogical approach that combines soccer metaphors and activities with accurate health information. Coaches' are trained in the "Big 5", crucial building blocks for adolescent-friendly facilitation: sharing accurate information, providing positive praise, sparking vital conversations, building a safe space, and creating person connections. SKILLZ Coaches aim to build the health and social assets of adolescents, facilitate access to health and social services, and

support adherence to medical treatment and promote positive protective behaviours.

2) The SKILLZ Package provides multiple points of access and direct linkages to facility-based, youth-friendly care, leveraging existing community- and facility-based resources. Coaches will be trained and certified by the Ministry of Health as Community-based Distributors (CBDs) of select family planning commodities and HIV self-test kits, integrating the direct provision of SRH services with their educational mission. The coaches will also be trained to provide basic metal health support to girls participating in the intervention.
3) Ensuring the SKILLZ Package ensures that comprehensive SRH and services are continuously available through CBDs-Coaches and referrals to health facilities and partner organizations, including a focus on pregnancy and HIV prevention, the two largest risks that adolescent girls face in their pursuit of security and independence in adulthood.

The SKILLZ Package entails four integrated programs that work together to build a continuous support system, that not only encourages uptake of contraceptive and HIV services, but also adherence and continued engagement with health services over the long-term (for pregnancy testing and referral to ANC, contraceptive methods, HIV repeat testing, preventive services such as PREP and/or HIV treatment and care).

1. **SKILLZ Girl** is a school-based program that provides adolescent girls (grade 10 or 11, regardless of HIV status) with an initial 12-session soccer-based comprehensive sexuality and SRH education curriculum; gender and power dynamics in relationships are also explored and addressed. This program is run during extra-curricular hours as part of school activities. The curriculum validates experiences of puberty and sexuality while building life skills and other critical assets that are broadly applicable to many spheres of girls' lives. It provides a link to a range of behavioural outcomes including SRH stigma prevention, SRH and well-being, and modern methods of contraception, all designed to create an integrated health package for girls. The curriculum culminates in a graduation ceremony at the SKILLZ Girl Health Event, providing girls with the opportunity to access community based SRH services and celebrate the successful completion of 12 curriculum sessions with parents/caregivers and community members. The SKILLZ Girl program was originally piloted in Zambia in 2015. Permission to continue to run the program was granted in 2019 by the MOGE. Prior to obtaining permission, modifications were made to the curriculum for this study to:
    - Introduce a focused module on HIVST, PrEP and contraception.
    - Provide basic information on mental health and well-being.
    - Include nurses as co-facilitators with Coaches in 4 selected SKILLZ Girl Curriculum sessions (Me, My Body and Mind, Winning Combination, Avoiding Risks and Let's get tested); and
    - Add an additional point of access to SRH commodity access through Coaches who will be trained and certified as CBDs (see below).

2. Convenient, high-quality, and adolescent-friendly health services

i) Community-based Distribution of Commodities**.** SKILLZ Coaches will undergo a 10-day Ministry of Health training and be certified as CBDs. MOH-certified CBDs are able to provide information, instructions, and distribute condoms, oral contraceptive pills, emergency contraceptives, self-injectable contraception (Sayana Press / DMPA-SC), pregnancy tests and HIV self-test kits. CBDs are

also certified to conduct traditional HIV testing and inject short-term contraception (up to 3 months (Depo-Provera/DMPA-SC). In addition to commodity distribution, CBDs will make active referrals to other SRH services, including long-acting reversible contraceptives and PrEP, through their relationship with health facility staff and other clinical service providers providing AGYW-friendly mental health and SRH services within the community (more detail in 2ii and 2iii). Coaches trained as CBDs will be able to distribute these commodities from the start of the SKILLZ Girl curriculum and distribute commodities through 3 modalities (Table 1):

Table 1: Summary of commodity, mapping and assessment

MODALITY, MAPPING & ASSESSMENT	AVAILABILITY & FREQUENCY
<u>Coaches' homes</u><br>Coaches' homes and households will undergo a readiness assessment focused on its acceptance of and support for commodity distribution to AGYW, and safety as a storage and distribution point.<br><br>To ensure that girls have easy access to commodities through the Coaches, the communities that participants reside in will be mapped to Coaches' homes. Using this information, Coaches will be allocated schools in communities where they come from or schools that are in close proximity to where they live. As the case is for most schools in densely populated areas, we anticipate that most girls will also come from communities that are in close proximity to participating schools.<br><br>Girls living farther away from their school, will be offered the option to access services and commodities from a different Coach than the one delivering the SKILLZ curriculum in school, but who is closer to her.	On-demand by appointment, anytime
<u>School premises</u><br>Coaches will inform girls of weekend distribution times and will pitch tents in intervention school grounds, where girls will be able to access select commodities and services.	On-demand, 2 weekends/month
<u>Community spaces</u><br>Girls wanting to access services during the weekdays can make an appointment with the Coach after school hours; this may be preferred by girls coming from more sparsely populated areas. Community distribution points (such as community halls, churches and open spaces) will be identified and assessed for readiness,	On-demand by appointment, weekdays, out of school hours

safety, and convenience. Coaches will use these distribution points to provide services as an additional alternative for participants not able to visit Coaches' homes or weekend distribution at schools.	

ii. PHQ-9 screening and active referral to Strong Minds Zambia (SMZ) for Interpersonal Group Psychotherapy (IPT-G). SMZ is a social enterprise that provides treatment for women who suffer from pervasive and debilitating mental illness. Coaches will be trained in the screening for depression using the PHQ-9 screening tool. The screening process will start from Session 3 in the curriculum, after rapport between Coach and SKILLZ participants has a chance to be established. All girls will be screened at least three times (at Session 3 of SKILLZ Girl, during SKILLZ Teen Club at 6 months, and at 12 months), hence referrals to SMZ will be continuous during the study period. All participants presenting severe depression (Score >9) will be referred to SMZ for enrolment into Interpersonal Group Psychotherapy (IPT-G), a 10-session, WHO-approved first line treatment for depression.

iii. Clinic-based SRH services. Girls will be referred to the youth friendly local clinic (supported by CIDRZ) Adolescent Friendly Corners/Spaces in Health Facilities for services that they cannot access from the coaches (i.e., long-term contraceptives and PrEP). CIDRZ will work closely with the MOH to ensure all clinic-based services needed by adolescents are readily accessible and available. To accomplish this, CIDRZ support a hybrid model of community- and clinic-based adolescent-friendly corners/spaces, including other clinical partners' access points, such as DREAMS Houses and adolescent-friendly health facilities Adolescent Friendly Corners/Spaces in Health Facilities to meet the specific demands of adolescents for SRH services and stigma-reducing initiatives.

3. **SKILLZ Teen Club**. Following the SKILLZ Girl Heath event, all girls (whether they test positive for HIV or not) will continue into SKILLZ Teen Club, facilitated by Coaches, who will continue in their role as CBDs to provide continuous, on-demand access to commodities and services. After completion of SKILLZ Girl, Coaches will continue with the same group of girls and facilitate the SKILLZ Teen Club until the end of the Grade 12 school year, with the support of the school-based Careers and Guidance Teachers (or another appointed teacher). These school-based clubs are run in the afternoons during extra-curricular hours and ensures continuous engagement in deeper discussions about SRH and services, goal setting, healthy relationships, equitable gender attitudes and norms, combination HIV prevention. SKILLZ Teen Club also focuses on contraceptive initiation and continuation as a tool for future planning, self-care, and empowerment. The club is an ongoing activity that students can participate in until they leave or graduate from school. Club structures are very widespread and have been operating in schools in Zambia since 2017 with the MOGE's consent as part of the Memorandum of Understanding (MOU) signed in 2010 (*see Appendix B*).

Figure 2 presents intervention map for girls enrolled in the SKILLZ intervention.

Figure 2: Intervention Map

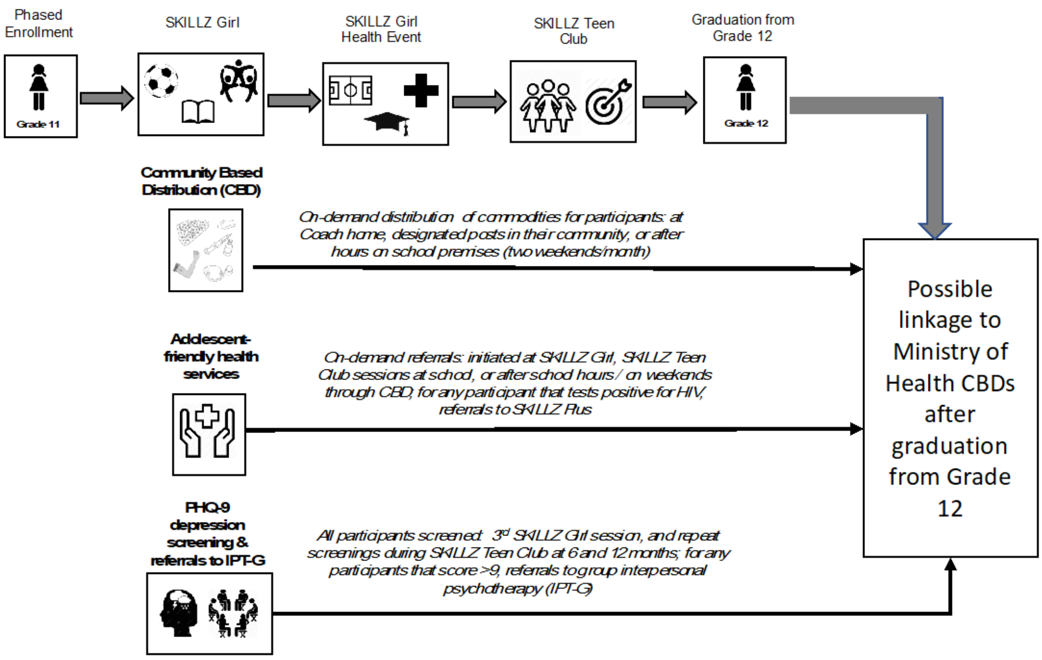

Standard of Care (SOC package): The primary objective of this study is to evaluate the impact of SKILLZ intervention package offered to selected schools on uptake of HIV testing and SRH services among girls aged 16 years and above when compared to the current standard of care in Zambian schools. In the SOC package, the girls will receive the standard comprehensive sexuality education as implemented by the school and in line with the current Ministry of General Education (MoGE) guidelines for delivery of comprehensive sexuality education in Zambia. Grassroot Soccer will work with school authorities to ensure to the degree possible that the girls also participate in the girls also participate in the SOC school-led clubs of their choice until they leave or graduate from school.

Our mixed-methods evaluation will assess the effects of SKILLZ offering (SKILLZ-Girl & SKILLZ Club programs) compared to the SOC offering School-led comprehensive sexuality education and SOC clubs only. Although the SKILLZ Program will be offered to all girls enrolled where the SKILLZ-Girl curriculum is offered by GRS at selected study secondary schools allocated to the intervention arm, only girls aged 16 and over at the commencement of SKILLZ-Girl will be eligible for enrolment into the research study.

We will examine outcomes for all eligible girls at the start of the intervention in up to approximately 23 schools where the SOC is offered, compared to outcomes in up to approximately 23 schools where the SKILLZ-Package is offered, including HIVST, the availability of expanded contraceptive services and the SKILLZ-Club following completion of SKILLZ-Girl. Therefore, all schools will be preassigned prior to the intervention into the following 2 categories (see **Figure 3** in *Section 8 - Methodology* below).

1. **SOC arm:** Comprehensive sexuality education as implemented by the school → Club SOC
2. **Intervention arm :** SKILLZ-Girl → SKILLZ-Club

Our central hypothesis is that the additional offering of HIVST and contraceptive services in SKILLZ-Girl combined with the SKILLZ-Club offering will increase HIV testing and contraceptive uptake and continuation.

In addition to SKILLZ-Girl and SKILLZ-Club, girls testing HIV-positive at any time during the study intervention period will be additionally offered to participate in the SKILLZ-Plus facility-based curriculum for HIV-infected adolescents. The effect of the SKILLZ-Plus curriculum will be compared using a SmartCare-derived matched (i.e., on clinic, age, ART start date and gender) population.

The impact of the SKILLZ Package on our primary outcomes—HIV testing and its care sequelae and contraceptive use—is likely to be moderated by girl's sociodemographic characteristics, such as age, SES status, and her home environment (i.e., siblings, parental HIV status).

3. Literature review

While historically overlooked, the consequences of neglecting to provide adolescents with strong SRH services and potentially causing a painful or damaging transition into adulthood are now well understood. For adolescent girls, pregnancies are much more likely to be high risk. Early pregnancies can also compromise their ability to complete their education, creating a knock-on effect to their future economic earning potential[34]. They also face an increased risk of exposure to HIV and sexually transmitted infections (STIs) as well as being involved in sexual coercion, and acts of gender-based violence[35]. Obstacles to receiving access to AFSRH services include inadequate social support, stigma, and detrimental gender and social norms associated with adolescent sexuality including high rates of intergenerational sex and intimate partner violence.[36-41] Health care workers often judge, reprimand and may also deny SRH services to adolescents because they believe they are too young to engage in sexual activity.[42] These issues are magnified for HIV-infected adolescents, where, unlike other chronic diseases among young people, adolescent HIV infection is set apart as a highly stigmatizing disease during a developmentally sensitive time when transitions in self, body and health care are at play.[43]

The most recent Demographic Health Survey (DHS) showed that while 73.3% of unmarried, sexually active adolescent girls reported not wanting a child in the next two years, only 20.6% were using any contraceptive method to prevent pregnancy and only 40.4% of sexually active adolescent girls aged 15-17 had had an HIV-test in the last 12 months[3]. The report also indicated that by age 19, more than a third of Zambian adolescent girls report having experienced sexual violence from a male partner and by age 18, a third have been pregnant.[3] Furthermore, programmatic data from CIDRZ suggest almost 50% of HIV-infected adolescents that know their status drop out of care within two years of initiating ART.

Cultural factors, such as the widespread belief in Zambia that a girl or woman should not say no to sex or propose the use of a condom, have obvious consequences to a poor understanding and access to SRH services.[44] These barriers to accessing services and adhering to risk reduction strategies exist across all levels of influence: individuals may hold fears and misconceptions of HIV and suffer from stigma surrounding adolescent sexuality; fears and anxiety can lead to a lack of social support from partners and peer groups that would otherwise be protective; families and communities are often the source of perceived stigma, and further reinforce inequitable gender and social norms that amplify girls' vulnerability; health systems are

often inaccessible and unfriendly to young people.[31-33] These myriad factors collide to prevent adolescent girls from reducing risk, accessing HIV and SRH services, and remain in care over the longer-term.

There is however increasing access to cost-effective and easy to use long-acting methods of contraception such as the 3-month intramuscular administrated depo medroxyprogesterone acetate (DMPA-SC). This has been specifically designed to overcome some of the major barriers to contraceptive use, including difficulty in accessing facility based SRH services. Research to date has consistently demonstrated its safety, effectiveness and acceptability to both users and health care providers[45] [46,47]. Furthermore, use of a contraceptive method has been associated with an increased likelihood to be tested for HIV and a decrease in high-risk sexual behaviors[48]. Unfortunately contraceptive failure and discontinuation is more common amongst adolescent girls than among older women[49] and only 20.6% of sexually active adolescent girls who reported not wanting to become pregnant reported that they were using any contraceptive method to prevent pregnancy.[3] While DMPA-SC is currently being scaled up in over 22 countries around the world, including Zambia, the national strategy for Zambia does not provide specific plans for addressing the SRH needs among adolescent girls. Thus, there is an urgent need to fill this gap and identify effective strategies for ensuring health equity whereby adolescent girls have access to the same expanded contraceptive method mix in Zambia.

HIVST is another recent technological innovation that has emerged as a promising strategy to provide individuals with an opportunity to test while bypassing some of the stigmatizing concerns of the clinical environment that are especially salient among adolescents. A pilot study amongst adolescents in South Africa using HIVST found fidelity to be very high (96.4%), with participants reporting high rates of acceptability and usability[50].

A review of adolescent-specific service delivery programs over 2001-2014,[8] only 11 studies were identified that addressed the HIV treatment cascade among adolescents, none of which met criteria for a rigorous, adequately powered, longitudinal program. Although the evidence base is thus weak, programs that are easily accessible, and provide integrated services and peer counselling were highlighted as promising strategies.

Programs integrating contraceptive services and HIV prevention and testing have showed promising results for reaching adolescent girls. A recent Teenage Pregnancy Prevention Program in South Africa showed improved attitudes amongst its participants, including plans to communicate with partners about teenage pregnancy and increased reports of condom use in those that were exposed to the intervention[51]. A Youth Friendly Health Service (YFHS) model in Malawi offering contraception alongside HIV-testing supplies, and using a curriculum based on gender empowerment and self-efficacy, led to higher health services utilization, including uptake of HIV testing services, condoms and hormonal contraception[52].

GRS has shown that soccer-based interventions combined with strong school and community partnerships and referral networks can change behaviours and make a significant impact on the health of young people.[53] In 2009 in South Africa, GRS piloted the SKILLZ-Girl program which addresses the unique needs of young women and adolescent girls. This program was then scaled in South Africa and expanded further into 13 countries in SSA. A recent evaluation of the SKILLZ-Girl program showed that participants significantly improved their knowledge and attitudes surrounding HIV risk for young women, negotiation skills, and sense of self-efficacy. In fact, 68.5% of girls that participated in the SKILLZ-Girl program underwent testing for HIV at the conclusion of the school-based curriculum.[54] Although SKILLZ-Girl has now served over 26,000 girls in 14 countries, it has never been rigorously evaluated for cost effectiveness and effectiveness.

4. Research questions

Our mixed-methods evaluation aims to assess the effect of the SKILLZ Girl on HIV testing and SRH related prevention services uptake (Aim 1), describe linkage to care and treatment and VLS and retention at 6, 12 and 18 months for girls who are identified to be HIV-infected during the study, using qualitative, programmatic, and administrative data (Aim 2) and identify mediating pathways and moderating factors leading to observed effects (Aim 3). Aim 3 will also assess the cost-effectiveness of the SKILLZ Package.

Our research questions are thus:

1. What is the impact of SKILLZ-Girl curriculum on HIV testing uptake among adolescent girls (aged 16 and over) over a period of 18-months?
2. What is the impact of enhanced SKILLZ-Club on HIV testing uptake among adolescent girls (aged 16 and over) over a 24-month period?
3. Among HIV-positive adolescent girls, what is the effect of the SKILLZ-Plus intervention on:
    i. retention in care and treatment at 6, 12, and 18 months
    ii. viral load suppression at 12 months?
4. What specific barriers to access to services do the SKILLZ interventions (SKILLZ Girl, SKILLZ-Club & Plus) overcome?
5. What are the mediators of the observed effects?
6. What are the moderators of the observed effects?
7. To what degree was the intervention implemented per protocol (fidelity)?
8. What is the short- and long-term cost-effectiveness of the SKILLZ Package intervention?

9. Research aim(s)/General Objective and Specific Objectives

Aim 1: (a) Assess the impact of SKILLZ over two years on **HIV testing** and (b) SRH-related prevention services uptake among adolescent girls aged 16-19 in up to 23 intervention schools (with enhanced HIVST, PrEP, and contraceptive method offerings) compared to up to 23 control schools using a quasi-experimental difference-in-difference approach; and

Aim 1: (b) Describe **linkage to care and treatment, and viral load suppression and retention** at **6, 12, and 18 months** for girls who are identified to be HIV+ during the study, using **qualitative, programmatic, and administrative data.**

This will be achieved by i) using a quasi-experimental difference-in-difference approach that compares girls aged 16 and over in approximately 23 SKILLZ control schools compared to approximately 23 schools where SKILLZ will be delivered with the enhanced HIVST and a variety of contraceptive method offerings; and ii) through a cohort model using pre-existing SmartCare data on HIV+ individuals in care to match to adolescent girls aged 16 and over from the same district (Lusaka) who are identified to be HIV+ at any time during SKILLZ-Girl or SKILLZ-Club participation.

Aim 2: Examine how the intervention works including lessons learned for future implementation by

1. conducting a process evaluation to identify mediators, predictors, and barriers to uptake of the SKILLZ-Girl, Club, and Plus curricula both quantitatively through mediation and moderation analyses

and qualitatively through a sequential explanatory approach using focus groups discussions, interviews and observation with coaches and girls; and by

2. monitoring fidelity

Aim 3: Estimate the short- and long-term cost-effectiveness and return on investment of the SKILLZ Girl, Club, and Plus curricula for improving health outcomes for adolescent girls.

3. Methodology

1. Study design

We will conduct a mixed-methods evaluation of the SKILLZ intervention using the following approaches:

A **quasi-experimental cohort** of school-aged 16-year-old and over females to be followed for a period of 18 month will be evaluated with a difference-in-difference (DID) approach to estimate HIV testing uptake and sexual/reproductive health services use. Primary and secondary outcomes will be measured at several timepoints longitudinally across up to 23 schools offering the SKILLZ-Girl and SKILLZ-Clubs program and up to 23 schools offering the "standard of care" (SOC) and SOC Clubs (see Figure 3). Recruitment will take 6 months on a rolling basis. The order in which schools will be selected will be random. Participants will be clustered at the school-level; we will enrol a random sample of girls, from each participating school, aged 16-years at the commencement of SKILLZ-Girl and who are in a grade where GRS is offering the SKILLZ-Girl curriculum

All enrolled girls will be followed over the 18 months' time period to assess SRH service uptake, including HIV testing. It is estimated that the majority (~98%) of girls testing for HIV will likely be HIV-negative, based on estimates from programmatic GRS data. While HIV-positive girls will continue to participate in the school-based SKILLZ-Girl and Clubs activities and followed for SRH use, they will also be offered contemporaneous enrolment into SKILLZ-Plus upon infection; retention in care and Viral Load Suppression (VLS) at 6, 12, and 18 months will be measured for this subset. See Figure 3 for details of enrolment flow.

Process evaluation: We will conduct a process evaluation to better understand the casual pathways and effect mediators and moderators using mixed methods approaches, including supplemental qualitative data collection. To investigate reasons why the program components (SKILLZ-Girl, Club & Plus) might fail or succeed in different schools or communities and assess intervention effect mediators and moderators for HIV testing and contraceptive use uptake we will use a mixed methods approach involving: (1) quantitative analyses of baseline survey data for testing how variables can moderate the observed impact of the intervention, and (2) qualitative data collected from a subset of SKILLZ participants in the evaluation cohort and their coaches to further understand potential mediating pathways outlined in Figure 1.

Fidelity monitoring: We will also include measures to monitor fidelity of the intervention implementation, including receptivity to and understanding of the intervention amongst participants, the perceived appropriateness and relevancy of the intervention for adolescent girls in school, and the extent to which the intervention was delivered per protocol in different communities and schools.

Economic evaluation: We will estimate the short- and long-term cost-effectiveness and return on investment of SKILLZ for improving health outcomes for adolescent girls.

Figure 3*. Figure 2 presents intervention and evaluation flow for schools randomised to the intervention arm.*

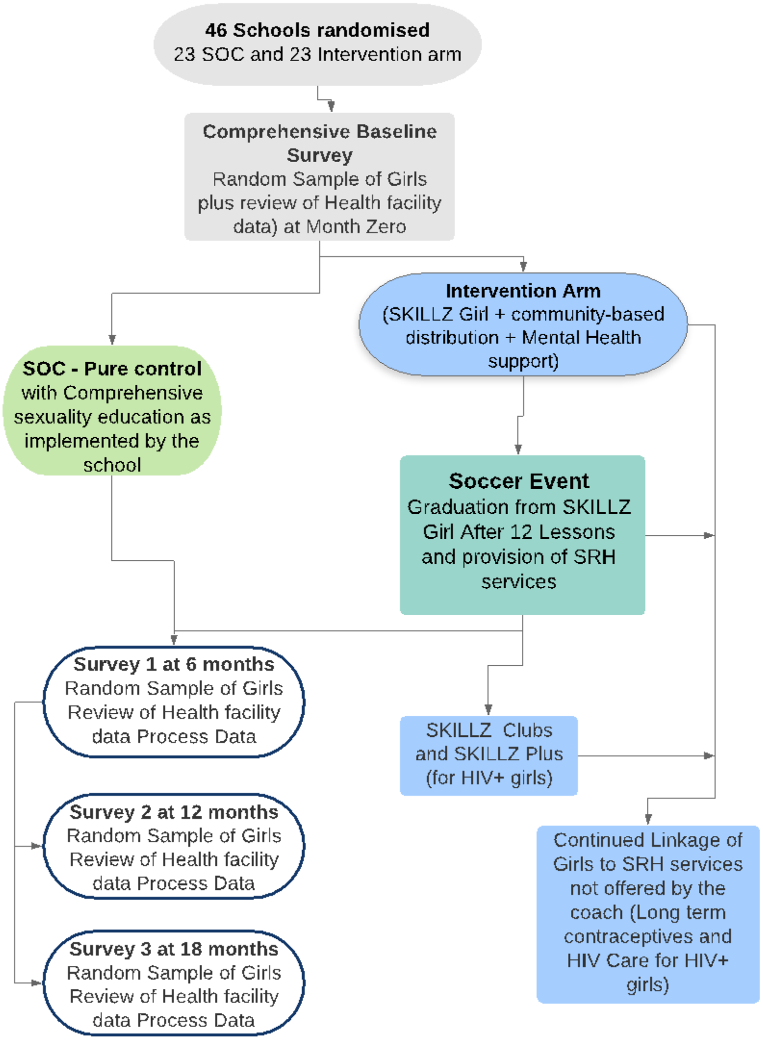

2. Study site and population/Research materials

The study will take place across all the highly densely populated areas of Lusaka, Chongwe, Chilanga and Kafue districts of Zambia where CIDRZ supports government MoH clinics with ARV services, electronic data management, and youth-friendly trained clinical personnel, and where GRS has been implementing their basic curriculum and events. All schools selected will be secular government schools. Approximately 80% of school-age girls are reported to be in school in Lusaka. To facilitate the evaluation of SKILLZ, all girls enrolled in up to 46 government secondary schools in Lusaka, Chilanga. Chongwe and Kafue districts that GRS currently serves will be eligible to participate in the SKILLZ program and will be randomly allocated to either the SKILLZ or the SOC implementation. For measuring impact, only girls aged 16 and over and enrolled in a Grade where GRS is offering the SKILLZ-Girl curriculum at the start of the intervention period will be surveyed and followed because youth aged 16 and over can consent for SRH/HIV services without additional parental

permission[55]. A similar cohort of girls will be followed in the control arm (See *Section 9: Ethical Considerations* for details on waiver of parental consent).

3. Selection of participants, sampling methods and sample size

Study Population: In each school, in both the intervention and control arm, we will recruit a random sample of girls enrolled in a grade 11 where who are at least 16 years old at the start of the intervention period (i.e., the commencement of SKLLZ-Girl in the intervention arm). We have designed our study to capture a minimum detectable effect size of a 20-percentage point increase in HIV testing, assuming a baseline testing rate of 60%, β=0.8, α=0.05, ICC=0.25 and an attrition rate of 20%. This requires 30 surveys per school at each of our 46 schools (n=1,380). We remain powered at an ICC=0.2 as long as baseline testing rates do not fall below 40%. However, this sample size calculation limits our ability to assess downstream effects on linkage to care and treatment, retention and viral suppression among HIV+ individuals as the estimated number of HIV+ girls will be too few to facilitate a sufficiently powered analysis. Despite this limitation, we will still follow any girls who are found to be HIV positive and collect programmatic data (through SmartCare) on retention and viral suppression and they will be included in our descriptive and qualitative analysis to understand their experience with the program and how this relates with their decision to engage and continue in care.

Cluster randomized controlled trial: We will select up to approximately 46 schools from three districts Lusaka (urban), Chilanga (peri-urban) and Kafue (peri-urban) to receive the SKILLZ-Girl curriculum. Of the total (up to approximately 46), half will be randomly allocated to host the SKILLZ-Girl curriculum, and the remaining half (e.g., up to 23) will be randomized to either the SOC arm. Those schools randomized to SOC will only have access to SOC SRH programs and comprehensive sexuality education as implemented by the school and SOC clubs. Those schools selected for SKILLS Girl curriculum will offer SKILLZ Club. **See Figure 4** for study allocation flow.

Figure 4. *School Selection Flow Chart*

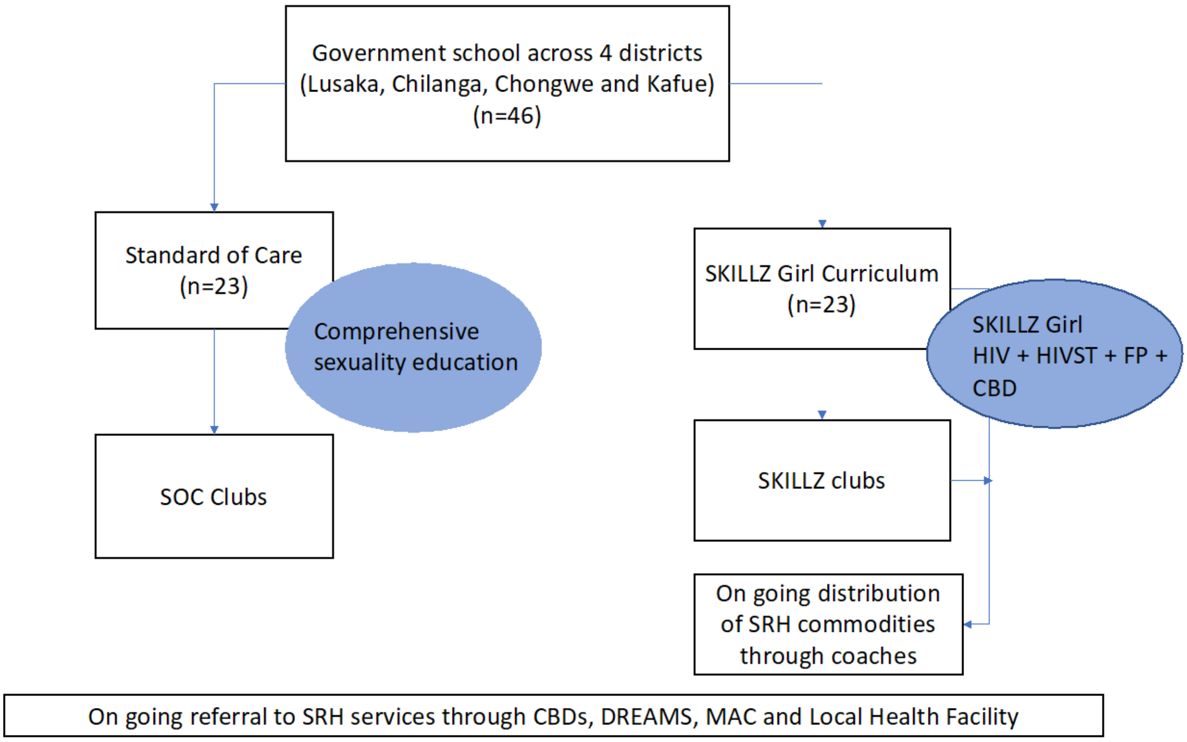

We anticipate that most of our evaluation cohort will not be HIV-infected. Thus, the retention to SRH analysis drives the sample size calculation of 351 girls using contraception at the end of 18 months years, with, and risk ratio difference of 0.3 (30%) between the SOC and SKILLZ-Club schools. To realize the 351 needed to meet requirements for estimate sensitivity, we augment the initial sample size receiving the SKILLZ-Girl curriculum accounting conservatively for 25% uptake of SRH methods and multiplying by two to assess the difference within each SKILLZ-Girl model (phase 1) and accounting for attrition across the two years of follow-up (12.5%) resulting in a starting sample size of 3159 (((351/0.25)x2)x1.025). **SKILLZ-CLUBS** will act as an extension to the SKILLZ-Girl curriculum enabling participants to seamlessly transition from the SKILLZ-Girl curriculum into the SKILLZ-Club. We anticipate minimal attrition (~15-20%). Participants will spend the majority of the study window in SKILLZ-Club and it is through the SKILLZ-Club affiliation that we will follow-up and ascertain outcomes for those enrolled longitudinally.

For the small minority of girls found to be HIV-infected across all schools where the study will be conducted, we will evaluate linkage to care and on treatment, VLS, and retention in care for a period of 18 months. These girls will be encouraged to continue on to the HIV positive-specific SKILLZ-Plus facility-based curriculum (if in the intervention arm) and SOC care for HIV+ adolescents as provided by the MoH for girls in the SOC arm . We will quantitatively and qualitatively describe linkage to care and treatment, retention and viral load at 6, 12, and 18 months after diagnosis (or since participant is identified by GRS as HIV+) using SmartCare data and compare to programmatic and administrative data that will be collected during the course of the study.

Process evaluation: From 6 purposively selected intervention schools, a subset of evaluation cohort study participants will be invited to participate in the qualitative research based on their HIV and SRH outcomes. In this study, we will use a sequential explanatory mixed method design[56] to explain the outcomes after each evaluation time point (post-test, 3 mons, 12 mons and 18 mons). A sampling list will be generated from the survey data that will include information on their HIV testing outcomes, sexual reproductive health behaviour including contraceptive uptake and sexual behaviours. Purposive sampling will be employed to deliberately select girls with differential outcomes to understand the mediating factors at play and how exposure to the intervention affects decision making around these issues. At each intervention school, coaches will be invited to participate in FGDs to discuss the factors affecting the implementation of the curriculum sessions as well as the distribution of commodities. To supplement this data, we will hold observations of specific sessions to learn on how sessions are being delivered by coaches as well as exit interviews with girls to understand adoption of the intervention. Permission for observations will be sought from the GRS programme while girls for exit interviews will be identified during the observations, specifically seeking out those with varying levels of participation who will be asked to provide consent to participate in the exit interviews.-Parents or guardians of girls enrolled in SKILLZ will be followed at their homes to ask for voluntary consent to participate in in-depth interviews. They will be selected based on their daughter's HIV and SRH outcomes. Lastly, girls becoming pregnant will be approached and asked to participate in narrative interviews.

Economic evaluation. No human subjects will be enrolled for this component.

4. Data collection plan and tools

SmartCare electronic data*:* All antiretroviral therapy services offered as part of the study will be captured in SmartCare, which permits measuring of utilization and clinical outcomes (i.e., Linkage to Care, VLS) at soccer events, mobile clinics, and public clinics in Lusaka. VLS is defined as below detection, i.e., less than 50 copies/ml. Retention in care will also be measured using SmartCare data on pharmacy visits and defined as accessing pharmacy within a 90-day window of visits. Consent will be requested from all participants as part of the consent process to access their SmartCare records.

Youth-Friendly services electronic data*:* Depending on the location of the school, the girl's residential address and/or her own personal preferences, participants will be referred to either the PEPFAR Funded DREAMS houses or the M.A.C. funded youth-friendly spaces based in local MOH clinics in order to access ongoing SRH services not provided by the coaches in their CBD role (this will include accessing PrEP, STI testing and long-term contraceptive methods). All interactions with the DREAMS houses or M.A.C centres (including meeting with counsellors, referral and uptake of services) are captured in their respective electronic databases. Consent will be requested from all participants as part of the consent process to allow researchers to access either DREAMS or M.A.C data should they chose to access either of these services.

Survey at baseline*:* A baseline survey will be administered to approximately 30 girls per school randomly selected to participate in the evaluation to collect information on school characteristics, sexual behaviour, age, HIV knowledge, maternal education, baseline HIV status, and sexual/reproductive health uptake and retention. These variables will be used as covariates and moderators for service uptake and retention. The

target sample of approximately 1380 girls will respond to a survey self-administered using ODk software upon informed consent. The survey will be available in English, Nyanja and Bemba. Coaches and study staff will on site be help girls navigate the survey. If during baseline, it appears that there has been sufficient time to measure change, or the full SKILLZ-Girl curriculum was not administered, the baseline survey may be re-administered to assist with validation of responses (as opposed to implementing the follow-up survey provided below).

Survey post-SKILLZ-Girl event, 6-, 12-, and 18 months: A total of approximately 30 girls per school (regardless of HIV status) will respond to a ODK survey to collect information on sexual behaviour, HIV knowledge, maternal education, sexual/reproductive health uptake and retention. Post-SKILLZ-Girl survey data will be used to assess the effect of SKILLS Girl and SKILLZ-Club on the outcomes of interest. In schools selected to offer SKILLZ-Club, surveys will be conducted during club attendance approximately 6 months after the SKILLZ-Girl community soccer event. Surveys will be completed individually by the participant on a secure tablet physically or via phone. Study staff will be available to assist participants to navigate the survey if they request assistance. If a participant has opted to not attend the clubs or has since left or changed schools, the coaches and study staff will attempt to trace participants in the community in order to complete the follow-up survey.

Where a participant is contacted for follow-up and it is discovered that she has left Lusaka, Chilanga, Chongwe and Kafue districts and it is not possible for study staff to arrange a visit to meet with her, a personalized survey link will be sent to her via email or WhatsApp (based on the participant's preference). She will then be invited to complete and submit the survey online. Alternatively, if the participant prefers study staff will complete the survey with the participant over the phone and enter their responses directly into ODK.

In the schools selected not to offer SKILLZ-Club, GRS will arrange for a 2-hour meeting session with all participants still enrolled at that school, where all participants will complete the surveys. If a participant opts to not attend the meeting or has since left or changed schools, the coaches and study staff will attempt to trace participants in the community in order to complete the follow-up survey.

The survey will record any changes to their home environment from baseline, again ask about self-reported sexual and risk behaviours and HIV knowledge (i.e., post-test for HIV education curriculum), and further quantitatively measure potential mediating pathways. Based on validated scales, the survey will include modules to assess stigma;[57] empowerment;[58] and self-efficacy[59] in general and specific to SRH/HIV;[60] disclosure, extent of social support from different sources (e.g., family, peers, teachers), and trust in health providers.[61] Individual lost to follow-up (LTFU) will be traced following standard procedures. Active follow up and tracing of late or lost patients enrolled into HIV care or receiving ART is part of the National treatment program. We will partner with pre-existing CIDRZ community volunteers responsible for follow up to ensure that efforts are synchronized, and that data are shared and entered into SmartCare. CIDRZ already has an international reputation leading effort to track those who are lost to ensure participants return for visits and viral load testing as per national guidelines.[98]

Process Evaluation:

Exploration of impact mechanisms. Because a number of hypothesized intervening pathway concepts may be difficult to quantitatively measure with sufficient precision in the survey (e.g., trust in health providers and facilities,[59] social support, empowerment), particularly for adolescents, we will collect a variety of qualitative data to further investigate factors that affect SRH uptake and HIV treatment outcomes (see **Table 2**). The GRS curriculums (SKILLZ Girl, SKILLZ Clubs, as well as SKILLZ-PLUS for HIV-positive girls) not only provide information on sexual reproductive health but promote powerful psychosocial concepts that are aimed at empowering girls to make the right choices with respects to sexual reproductive health for both HIV-negative and positive girls. Through a phenomenological approach, we aim to understand how exposure to the GRS curriculums influence decision-making for uptake of SRH and HIV services. We will further explore girls' experiences, personal beliefs, perceptions of the GRS program content and interactions with peers, coaches, parents, health care providers and SRH products and how these affect their decision-making process. These will be explored in the context of SRH and HIV outcomes, taking into consideration those with low and successful uptake of HIV and SRH services.

Using a narrative approach, we will document individual stories regarding how girls who become pregnant and/or HIV infected experience the mediating factors (such as stigma, disclosure, social support and trust in the health facility) and how these factors affect their decisions regarding sexual reproductive health and pregnancy among those who become pregnant. These narratives are valuable in revealing the process of problem identification to resolution.[60]

The second objective of the process evaluation collection is to complement the collection of quantitative process measures regarding the implementation of the three SKILLZ curriculums, namely, Girl, Clubs, and Plus as well as SRH commodity distribution. This will allow us to gain a better understanding of factors related to implementation fidelity, how girls perceive and react to the different SKILLZ program components (dose received), and the reasons for (differential) uptake of different program components. Given that multiple GRS coaches will be involved in delivering the intervention, adaptation of content or delivery will be assessed through unannounced observations of the sessions followed by exit interviews (after a session) with the participants (dose received). A key component of the SKILLZ curriculum is to promote parental support for SRH by encouraging girls to speak with their parents/guardians about these issues including disclosure of HIV test results. To understand how this happens and its effect on girl's decision making, we will hold in-depth interviews with parents/guardians. The process evaluation will also include **extraction of programmatic data:** Quantitative measurements coming from programmatic data (e.g., frequency and attendance at SKILLZ meetings, frequency of home visits, observations of SKILLZ sessions) will be collated from reports, supplemented with interviews of implementing staff (e.g., program managers and coordinators, coaches) to track key process indicators. This will also be triangulated with programmatic-level information from the qualitative interviews.

Study Populations

The following will be included in the process evaluation:

Adolescent girls aged 16year and above enrolled in SKILLZ Girl, Club and SKILLZ Plus
Coaches
Parents and guardians of adolescent girls enrolled in SKILLZ
Collaborating partners (MoH, MoGE)

5. **Sample Size**

We will purposively select five schools (N=5), two from Lusaka district and one each from Chilanga, Kafue and Chongwe districts to account for contextual differences. In each school, we will conduct 18 IDIs with adolescent girls at three data collection time point (in-line with the survey data points); IDIs will address either contraceptive outcomes or PREP and HIV testing outcomes in order to reduce interview length. We will also hold one FGD in each school with adolescent girls during the SKILLZ Girl and SKILLZ Club components (see Table 3). Adolescent girls falling pregnant in any of the intervention schools will be targeted for narrative interviews. We will hold three unannounced passive observations and subsequent exit interviews with three participants of the curriculum sessions observed. Additionally, we will hold one FGD with coaches in the selected schools. Two IDIs with parents/guardians will be targeted per school. Four FGDs (one in each district) will be held with household members of coaches involved in the home distribution of commodities. In each district we will hold one KII will be held with a member of staff from a health facility and a school (N=10). In situations of community unrest or COVID, the interviews will be conducted via phone. Selected data collection methods will take place directly after the four following time points, with survey responses driving sample selection as needed (See Table 3):

Time point 1: After completion of the first follow-up (or baseline) survey (conducted 1-2 weeks) after being offered voluntary counselling and HIV testing at the SKILLZ Soccer Event, regardless of decision to test or not

Time point 2: After completion of the second follow-up survey (conducted after the completion of the formal component of SKILLZ-Club or approximately 3 months after the SKILLZ Soccer event

SKILLZ-Plus: For the HIV infected cohort, we will hold 6 interviews with girls who have completed the SKILLZ Plus curriculum in the health facilities.

Table 2: *Research Questions by Data Collection Method*

**SKILLZ Girl, SKILLZ Club**	**SKILLZ Plus (HIV Positive only)**	**SKILLZ CBD**	**Method**
**Impact Mechanism:** How do psychological, social and structural factors affect HIV testing, contraceptive use and PREP uptake?<br><br>**Process (dose received):** What factors in SKILLZ Girl, Enhanced Girl and Club curriculums influence girls' uptake and use of contraception, PREP and repeat HIV testing?	**Impact Mechanism:** How do psychological, social and structural factors affect HIV drug adherence and retention in HIV program?<br><br>**Process (Dose Received):** What factors in SKILLZ Plus curriculum influence girls' adherence to HIV drugs and retention in HIV program?	Impact Mechanism: How well have coaches adopted the SKILLZ CBD model?	IDIs
**Impact Mechanism:** How do school social (values, norms) and structural (type of school) factors affect the perception and uptake of SRH services?		**Impact Mechanism**: How well is the community CBD model accepted by families of coaches doing the distribution?	FGDs
**Process (dose received):** How do girls perceive the relevancy and appropriateness of the different intervention components (SKILLZ Girl, Enhanced Girl and Club Curriculums)?			Exit interviews (for process only)
What mediating factors (emotions, experiences, stigma/disclosure/social support/trust) affect the uptake of SRH services and girls' response to pregnancy?<br><br>How does engagement with SKILLZ			Narrative interviews

Girl/PLUS/CLUB affect decision making and 29behaviour?			
**Process (Fidelity/ adaptation):** How closely do coaches follow the curriculum and how do they adapt or modify their delivery based on barriers to implementation?			Passive Observations
Impact Mechanism: What contextual factors affect the uptake of the HIV and SRH services in the schools.	Impact Mechanism: What contextual factors affect the uptake of the HIV and SRH services in the health facilities	What contextual factors affect the uptake of the HIV and SRH services in the community distribution models?	KIIs

Table 3: Sample Size and Data Collection Time Points by Study Arm (Per School)

Data collection method	Time point 1:	Time point 2:	Time point 3:
IDIs with girls	6	6	6
FGDs with girls	1	1	1
Exit interviews with girls	9		
Narrative interviews with girls	N/A depending on girls' experiences		
Passive observations	3		
FGDs with coaches	1		
KIIs with focal point persons	2		
IDIs with parents and guardians	2		

*One per district

Extraction of programmatic data: Quantitative measurements coming from programmatic data (e.g., frequency and attendance at SKILLZ-Plus meetings, frequency of home visits) will be collated from reports, supplemented with interviews of implementing staff (e.g., program managers and coordinators, coaches) to track key process indicators. This may also be triangulated with programmatic-level information from the qualitative interviews.

6. Data management and storage

Quantitative: We will collect individual-level data on clinical, laboratory, and demographic characteristics, including retention in care and VLS at 6, 12 and 18 months. Using case reporting forms (CRFs), we will collect study-specific data, including information obtained directly from participants, routine programmatic data from GRS and abstract routine clinical data from paper-based medical records, the SmartCare Electronic Management Records (EMR), CIDRZ Laboratory Information Management System (LIMS) and DREAMS electronic management system. Routinely collected individual-level clinical data are first written onto forms in the paper medical record and then entered into the SmartCare EMR. SmartCare serves as a repository of clinical data for HIV-infected individuals and includes such data fields as: ART initiation date, ART regimen, visit dates, and laboratory data.

GRS will collect programmatic data through a combination of paper and electronic data entry via tablets, with all data stored securely in ODK. Where paper files are used for programmatic management, data will be manually entered by study staff directly into the ODK database. De-identified data will made available to GRS co-investigators to allow them to view key fidelity and quality indicators in order to monitor implementation in real time.

All paper study files will be stored in secured; locked cabinets located in locked rooms available to study staff only. The keys to the locked cabinet will be kept by study staff and not made accessible to non-study personnel.

A secure server will be used to store encrypted study data, including the study database. The research database will be developed using ODK and hosted on the secure CIDRZ server. Scheduled backups will be done routinely. A password will be required to gain access to the study dataset, and access will be restricted to key study personnel only. All personal identifiers will be removed prior to generating the analytical dataset.

Qualitative: All data will be obtained using digital audio recorders. Recorded data will be transferred on to a lockable computer kept by the co-principal investigator leading the qualitative research. Recordings will be shared with transcribers who will transcribe the voice recorded data into verbatim. Transcripts will go through a QA/QC process where researchers will cross check randomly selected transcribed verbatim with the recordings. Where errors are found, changes will be made to reflect the recordings accurately. Once this process is done and the quality of transcripts has been assured, recordings will be destroyed. Transcripts will be kept by study staff and stored electronically in a password protected computers accessible only to the 3 researchers. Transcripts will also be printed for analysis purposes and kept in a designated room in locked

cabinets accessible only to the Co-PI. All qualitative data will be destroyed after 3 years in line with MOH recommendations.

7. Data analysis plan

Difference-in-difference model. The primary outcome is the probability of testing for HIV at any time during the period when SKILLZ is delivered through to 18 months after the commencement of SKILLZ Girl. The primary analysis for Aim 1 will follow a difference-in-difference (DID) effect between study arms. The outcome of interest will be regressed on an indicator of study arm assignment interacted with a dichotomous variable POST which indicates the outcome after the intervention is implemented; this differentiates baseline and end-line for each study arm, controls for any time dependent variables that might confound the effect, as well as any time-invariant observed or unobserved confounders that might differ between study arm schools. Because of this, the DID is robust to time-invariant unobservable confounders (e.g., school quality) and time trends (e.g., secular increase in testing). Our estimator relies on the assumption of common time trends across intervention and control in absence of the intervention, and of static population. Additionally, we control for baseline individual and school-level moderators that may be unbalanced at baseline after randomization. School-level controls include size, promotion rates, sex composition and repetition and dropout rates (available in the district education office) and individual girls' controls include age, grade, maternal education, HIV knowledge. Clustering by school will be accounted for by a generalized estimating equation approach. We will estimate intention-to-treat (ITT) by including all girls in the evaluation cohort assigned to each arm rather than all girls who actually participated in SKILLZ.

Individual-level matched cohort analysis for HIV-infected: The analysis will follow an individual ITT estimator among individuals found to be HIV-infected, who will then be matched (based on clinic, age, ART state date, and gender) to HIV-infected individuals in the SmartCare database. The outcomes are the proportion of enrolled HIV+ girls who linked to care, who are virally suppressed and retained in care at 6, 12, and 18 months after diagnosis of HIV+ (using national electronic HIV medical record data). All data will be analysed using STATA.

Process evaluation: This will involve quantitative and qualitative analyses:

<u>Quantitative analysis</u>: Surveys conducted at baseline will be used to identify differential effects among girls who do and do not take up services according to baseline moderating variables as specified in **Figure 1** and as assessed by previous sexual behaviour, HIV testing/status, knowledge, maternal education by interacting the main exposure by these indicators. Statistical mediation will be assessed using the causal inference-based approach of Valeri et al., which yields optimal estimates of indirect effects in the presence of binary outcomes and moderator-mediator interactions,[64] and robust standard errors to address clustering of individuals within facilities, and account for multiple causally depending mediators.[65] Significant indirect and total effects of integration markers on outcomes accompanied by non-significant direct effects will signify mediation. The interplay between exposure, effect modifiers, moderators and outcomes in the causal chain is represented in **Figure 1**. For analyses, we will use mixed effect structural equation models in STATA and MPLUS with bootstrapped standard errors to test the contribution of indirect effects.

Qualitative analysis: All interviews will be voice-recorded, transcribed and coded using a thematic analysis process[66] (e.g. in NVIVO or Atlas.ti). Analysis will begin with familiarization of the data that will involve reading and re-reading of the transcripts to understand the data. Notes will be taken down at the point of familiarization to guide the development of the codebook. Codes will be extracted both inductively (bottom-up) and deductively (top-down). Inductively, open coding will be conducted to identify interesting and meaningful text in each line of the transcripts. Each piece of text will be grouped under a specific code that describes the meaning of the text. Once all the text has been coded, the researchers will then identify themes that tell something significant about the research questions and group a set of codes under each related theme. The themes will then be reviewed by reading each code and its associated text and re-arranged or removed in order to refine the themes and their meaning. Secondary coding will be applied to uncover the latent meaning of each of the themes. Themes will then be re-coded to uncover the latent meaning of the themes and refine their definitions.

Deductively, attributes from the RE-AIM process evaluation framework will be used to create the themes.[67] These attributes are "reach, effectiveness, adoption, implementation and maintenance." Transcripts will be coded under each of these attributes and subsequently be refined using the same process described above.

Fidelity monitoring: *Fidelity* will be measured by recording number of visits for coaches in the intervention and attendance, number of soccer events and attendance, number of home visits by coaches (e.g., for missed visits), as well as with key-informant interviews with coaches and with the study coordinator to understand how the intervention was implemented and will report in any discrepancy from protocol. Reception of the intervention. During the FGDs we will assess how the participants understood and used the intervention. Appropriateness and relevancy: During the FGDs to coaches and girls we will inquire about appropriateness and relevancy of soccer as a platform for adolescent programs. Reach of program: this will be captured by number and proportion of referrals (using SmartCare) to the health facilities that were successfully linked, as well as the presence of adolescent friendly corners at facility, number and type of adolescent clinical services delivered.

Economic evaluation: We will assess cost-effectiveness in three steps. First, we will assess the net cost of the intervention and its consequences, using micro-costing and modelling techniques. Costs for the intervention itself will include mainly personnel to manage the intervention and the financial incentives. Costing of the intervention and control models will be captured in costing sheets that will be prepared before the start of the intervention aimed at capturing costs as they are spent; we will limit the costing from the provider perspective and will only limit costing to programmatic and not research costs. We will also cost the change in health care utilization precipitated by the intervention: both increased preventive care and decreases in curative care. This will be assessed combining utilization with standard values for unit cost (per MOH official records). Together, these generate net costs. Second, we will quantify the health benefits of the intervention, including health events (e.g., number and type of opportunistic infections, STIs) and Quality-Adjusted Life Years (QALYs) which translate morbidity in the short and long term (projected based on published longitudinal clinical studies) into a standard metric of health status. Health state utility data (for QALYs) will derive from studies in the literature. The analysis of expected costs and QALYs in the intermediate and long-term will be structured using a spreadsheet-based decision analysis created for this purpose in consultation with clinical experts. Third, we will calculate the economic outcome measures

including the Incremental Cost-Effectiveness Ratio (ICER) and Return-on-Investment (ROI). The ICER is the ratio of net cost to health gain, comparing the SKILLZ-Plus to control (e.g., ICER for HIV testing = (C_intervention – C_control)/(Testing rate_intervention – Testing rate_control)), and is indicated if the intervention has a net cost (i.e., does not save money) while improving health outcomes (QALYs gained). All analyses will be repeated for 1, 2, 5, 10, & 20 years, and subjected to extensive sensitivity analyses to test the impact of uncertainty on results.

8. Ethical considerations

This study proposes to evaluate the impact of the SKILLZ Package (SKILLZ-Girl, SKILLZ-Club and SKILLZ-Plus) on the uptake of HIV and SRH services including uptake and adherence to contraceptive services, HIV testing, linkage to care and adherence, VLS and retention in care. This will be done by the review of GRS programmatic data, conducting surveys and implementing IDIs and FGDs with adolescent girls aged 16 and over as well as program staff involved in the implementation of the SKILLZ package. It will also require access to adolescents' medical records in order to evaluate impact on the HIV outcomes mentioned above.

Parental Consent. Prior to the commencement of the SKILLZ-Girl program, consent forms will be sent to parents/guardians of all girls who will be aged 16 and 17 years at the commencement of SKILLZ-Girl randomised to participate in the evaluation. Parents will also be invited to attend a series of sensitisation sessions at the schools. Parents whose children will be randomly sample to participate in the cohort study who attend the sensitisations will be asked to provide written consent. Study staff will follow-up all parents that do not attend these sessions by phone and will request to visit the parent at their home to obtain written consent. If the situation in Lusaka is not conducive to home visits due to COVID, political or community unrest and parents are not comfortable or able to attend the school, study staff will review the consent over the phone and complete a verbal consent with the parent/guardian. This consent will be used to allow the study staff to gain assent (verbal or written) from the potential participant. If or when the situation is conducive to home/community visits and the parent consents to a visit study staff will obtain a written consent. Study staff will only obtain assent from participants randomly selected for the evaluation and whose parents or guardians have previously given consent (verbal or written). If possible, participant information sheets will be given to parents/guardians in a language of their choice, either Nyanja, Bemba or English, all common in Lusaka. If they are unable to read (i.e., illiterate), a witness aged 18 years and over (not directly involved in the study) will be invited to be part of the consent process to verify that the information being given by the research assistant is accurate. An impartial witness will oversee all phone conversations for verbal consent. The information sheet will contain information including the purpose of the study, the procedures and risks and benefits. The right to withdraw at any time will also be emphasizes to all participants.

Informed Assent. Girls aged 16 and 17 years randomly selected to participate in the SKILLZ study, and whose parents have given prior consent, will be approached by the study team for their assent to participate. The same participant information sheets will be given to them in a language of their choice between Nyanja, Bemba and English, all common in Lusaka. If they are unable to read (i.e., illiterate) , a witness aged 18 years and over (not directly involved in the study) or a teacher (only if the adolescent is comfortable) will be invited to be part of the consent process to verify that the information being given by the research assistant is accurate. The right to withdraw at any time will be emphasizes to all participants including their parents/guardians. While sensitisations may take place in groups, individuals will be consented privately to

maintain confidentiality. Similar to the consenting process, verbal assent will be obtained over the phone if the situation in Lusaka is not conducive to meet the girls in person due to COVID, political or community unrest. The study staff will explain all information related to study participation and complete a verbal assent with the participant via phone. Once the situation is conducive for in-person meetings and schools are re-opened, the study staff will obtain a written assent.

Informed Consent:
Girls aged 18 years and above who will be selected to participate in the study will be approached by the study team without prior consent from their parents and will be invited to complete a consent form in a language of their choice (Nyanja, Bemba or English). If they are unable to read (i.e., illiterate), a witness aged 18 years and over (not directly involved in the study) or a teacher (only of the adolescent is comfortable) will be invited to be part of the consent process to verify that the information being given by the research assistant is accurate. The right to withdraw at any time will be emphasizes to all participants. While sensitisations may take place in groups, individuals will be consented privately, in a separate room or via phone (if the situation is not conducive to meet in person), to maintain confidentiality.

Involvement of Vulnerable Populations. We are targeting adolescent girls aged 16 years and over. We are aware that the population we would like to include in our study are considered a vulnerable population in line with the World Health Organization guidelines as well as the Zambian guidelines. The evaluation is targeted at this group to see whether the benefits of HIV prevention and treatment services empower girls to seek HIV and other family planning services without shame or fear and contribute knowledge that may be applicable to countries with similar settings.

Potential Risks and Protection against Risk.
Psychosocial Distress
The potential risks to participants of this study are minimal. We are aware that the adolescents targeted for this study share unique characteristics from the adult population that need to be considered throughout the roll out of the study. Adolescents participating in this study may experience psychological distress such as sadness, anxiety, distraction or stress when being surveyed or interviewed. To address this all field staff will be trained to offer basic psychosocial and metal health screening, referral resources, social service, and other emergency needs.

Stigma
Stigma may also arise as a result of follow-up visits by research assistants. Due to various HIV related studies and programs, follow-up visits in the community by an identifiable clinic staff is usually perceived as an adherence visit for one who is HIV positive. Therefore, follow-up visits conducted during the course of this study, may be perceived as such and lead to HIV related stigma for any members of a household. To avoid this, research assistants will be dressed as regular members of the community with no specific identifying information such as T-shirts, ID’s or badges. ID’s will only be shown once inside the home or when requested by local authorities.

Physical violence
Increased community unrest in Lusaka may result in parents feeling it is unsafe to travel to the school for a sensitisation or study staff feeling it is unsafe to travel in the communities to visit parents. To mitigate these

risks, we will allow parents to consent via phone to ensure that safety is not compromised in obtaining informed consents.

Loss of Confidential Data:

It is highly unlikely that confidential data will be lost during this study. However, we know that it is possible that other unauthorized personnel may view the data, or the data may be stolen or lost. In such cases, measures will be implemented to ensure no harm is posed to pupils. Except for the consent form, no identifying information will be collected or documented during assessments, analysis and reporting. Teachers and other school staff will not be allowed to see any confidential information unless it is the wish of the parent. Only coaches and selected clinical study staff will be allowed to have contact information of pupils in order to facilitate HIV testing and adherence counselling services. It will be the responsibility of these staff to use their contact information for the purpose of follow-ups only. They will be trained in research ethics where confidentiality will be emphasized. All identifying information will be stored in a separate password protected database from other project data. Consent forms will be scanned by study staff and stored in the password protected database. The original paper copies will be kept in a locked cabinet in a secure room at the CIDRZ offices.

Potential Benefits.

Study participants will receive no direct benefits for participating in the study. They will not be given any money for participating in the study. Those pupils invited to attend a focus group discussion will be given refreshments during the discussion. In cases where focus group discussions are held on weekends, students will be given a transport reimbursement in addition to refreshments. A K100 will be provided for transport reimbursements and lunch allowances where necessary.

9. Timelines

Timeline	2018		2019				2020				2021				2022				2023			
	Q3	Q4	Q1	Q2	Q3	Q4	Q1	Q2	Q3	Q4	Q1	Q2	Q3	Q4	Q1	Q2	Q3	Q4	Q1	Q2	Q3	Q4
Protocl development and planning																						
IRB/ REC submission																						
IRB/REC Approval																						
Study preparation																						
Recruitment, Baseline survey, preliminary analysis (Pilot Schools)																						
COVID Delay																						
Recruitment (Main study)																						
Participant follow up (18 months)																						
Data cleaning and analysis																						
Study close out activities																						
Dissemination of results																						

10. Budget

	Year 1	Year 2	Year 3	Year 4	Year 5
Salaries and Wages	ZMK 235,082	ZMK 235,082	ZMK 235,082	ZMK 262,469	ZMK 262,469
Fringe Benefits	ZMK 73,838	ZMK 73,838	ZMK 73,838	ZMK 83,536	ZMK 83,536
Personnel Costs (Subtotal)	***ZMK 308,920***	***ZMK 308,920***	***ZMK 308,920***	***ZMK 346,006***	***ZMK 346,006***
Travel				ZMK 37,624	
Other	ZMK 82,163	ZMK 82,163	ZMK 82,163	ZMK 82,163	ZMK 82,163
Subaward/Consortium/Contractual Costs	ZMK 5,380,354	ZMK 4,901,077	ZMK 4,880,127	ZMK 4,835,098	ZMK 4,629,423
TOTAL Direct Costs	***ZMK 5,767,853***	***ZMK 5,292,159***	***ZMK 5,271,210***	***ZMK 5,300,891***	***ZMK 5,057,591***
Facilities and Administrative Costs	ZMK 423,522	ZMK 101,679	ZMK 101,679	ZMK 121,100	ZMK 111,318
**TOTAL COST**	**ZMK 6,191,375**	**ZMK 5,393,839**	**ZMK 5,372,889**	**ZMK 5,421,991**	**ZMK 5,168,909**

11. Appendices

1. Letter of Permission from MOGE for GRS to continue working in schools in 2019

The picture can't be displayed.

2. MOU between GRS and MOGE

3.

The picture can't be displayed.

The picture can't be displayed.

The picture can't be displayed.

Status of Zambian GRS Personnel

5.4 Zambian GRS staff shall be employed in line with the provisions of the Zambian law.

5.5 All Zambian staff employed by GRS shall be given equal opportunities for responsibility, growth and development as their non-Zambian staff colleagues and shall be treated with mutual respect.

5.6 Zambian GRS personnel shall be required to pay all the taxes in accordance with the relevant provisions of the Laws of Zambia.

ARTICLE 6

6.0 **Financial Obligations**

6.1 This MoU imposes no financial obligations on either Party.

6.2 The Parties to this MoU understand that any financial arrangement entered into will have to be negotiated and will depend on the availability of funds.

ARTICLE 7

7.0 **Liabilities and Indemnities**

7.1 Subject to the provisions of this agreement, GRS personnel shall at all times be subject to such laws and regulations that exist in Zambia

7.2 MoE shall not be liable to indemnify any third party in respect of any claim, debt, damage or demand arising from the action taken by GRS or its representatives acting not under authority of control of MoE.

ARTICLE 8

8.0 **Entry into Force, Duration, Termination and Addresses**

8.1 This MoU shall come into force upon both Parties appending their signature thereto, pursuant to Article 8, and shall remain in force for a period of three (3) years and may be renewed for a further term of three (3) years by mutual written agreement of both Parties.

8.2 This MoU may be terminated at any time upon either MoE or GRS providing three (3) months notice in writing of intention to do so.

8.3 All notices or other communications under or pursuant to the Agreement shall be delivered in writing to the offices or addresses indicated hereunder.

ARTICLE 9

9.0 **Dispute Resolution**

9.1 Any dispute arising out of the interpretation, application or implementation of the provisions of this MoU shall be settled amicably through consultation, negotiations and mutual consent.

ARTICLE 10

10.0 **Jurisdiction**

10.1 The Parties submit to the jurisdiction of the Republic of Zambia

ARTICLE 11

11.0 **Notices**

11.1 The Parties choose their *domicilia citandi et exectuandi* for all purpose in connection with this Agreement at the following addresses;

(i) Andrew Phiri
The Permanent Secretary
Ministry of Education
Corner Chimanga and Mogadishu Road
P.O. Box 50093
LUSAKA,
ZAMBIA.

(ii) Naomi Walston
Secretary
Grassroot Soccer Education Limited
252B Twin Palm Road, Ibex Hill
LUSAKA,
ZAMBIA.

ARTICLE 12

12.0 **Amendment**

12.1 This Agreement may be amended or replaced by mutual consent in writing and with the signatures of the Parties.

ARTICLE 13

13.0. **General**

13.1 MoE and GRS respectfully confirm that they have taken all actions and secured all approvals necessary to authorize the execution and performance of this MoU.

13.2 Headings in this MoU are for convenience and shall not affect the interpretation of any provision of this MoU.

IN WITNESS WHEREOF the undersigned being duly authorized by their respective representatives, have caused their hands and seals to be to be hereby affixed the day and the year first before written.

Done at LUSAKA this.......19th.............. day of.......July...........2010

For the Government of
The Republic of Zambia:

Name: **Andrew Phiri**

Post: **Permanent Secretary HRA**

Date:..

Signature:..

WITNESS

Name:.....Dr F.N. Phiri.....

Post:.....DPI.....

Date:..

Signature:..

For Grassroot Soccer Education Limited:

Name: **Naomi Walston**

Post: **Secretary**

Date:.....19th July 2010.....

Signature:.....Naomi Walston.....

WITNESS

Name.....Thomas Lobben.....

Post:.....Dir. Ops - GRS.....

Date:.....19/7/10.....

Signature:..

8

1. Letter of Permission between MOH and GRS

All Correspondence should be addressed to the
Permanent Secretary
Telephone: +260 211 253040/5
Fax: +260 211 253344

In reply please quote

No.................

REPUBLIC OF ZAMBIA

MINISTRY OF HEALTH

NDEKE HOUSE
P. O. BOX 30205
LUSAKA

MH/101/15/17

20 June 2013
The Lusaka District Medical Officer

RE: Authorization to carry out HIV positive adolescent evaluation in Lusaka health centers

The Ministry of Health (MoH) in collaboration with Grassroot Soccer, IDinsight and the Center for Infectious Disease Research of Zambia (CIDRZ) is working to improve policy and guidelines for the treatment, care, and support of adolescents living with HIV/AIDS.

One of the interventions being supported to increase this understanding is the Grassroot Soccer's SKILLZ Plus curriculum, an interactive HIV and life skills education program for adolescents living with HIV/AIDS led by HIV-positive peer counselors through support groups in Lusaka district health centers.

The MoH would like to assess the impact of the SKILLZ Plus curriculum in order to generate evidence for future planning.

A research team including the MoH, Grassroot Soccer, IDinsight, and CIDRZ has been organized to carry out this important intervention and evaluation in health centers in Lusaka district.

Your usual support and cooperation will be appreciated.

[signature]
Dr. Peter Mwaba
Permanent Secretary
Ministry of Health

[illegible]
MINISTRY OF HEALTH
LUSAKA DISTRICT MEDICAL OFFICE
20 NOV 2013
DISTRICT MEDICAL OFFICER
P.O. BOX 50827
LUSAKA, ZAMBIA

No objection
Kindly facilitate
the team from
Grassroot soccer.
[signature]

2. Letter of Support from MOH regarding PREP uptake amongst adolescent girls

All Correspondence should be addressed to the Permanent Secretary
Telephone: +260 211 253040/5
Fax: +260 211 253344

In reply please quote:

No............................

MH/101/23/9

REPUBLIC OF ZAMBIA

MINISTRY OF HEALTH

NDEKE HOUSE
P. O. BOX 30205
LUSAKA

13th February, 2019

Carolyn Bolton Moore, MD, MSc
Centre for infectious Disease Research in Zambia (CIDRZ)
University of Alabama Birmingham, USA
LUSAKA, ZAMBIA

Dear Dr. Bolton Moore,

RE: LETTER OF SUPPORT FOR NIH-FUNDED APPLICATION: "IMPROVING PrEP UPTAKE AND RETENTION AMONGST ADOLESCENT GIRLS IN LUSAKA, ZAMBIA."

Iam delighted to write this letter of support regarding the above-mentioned project. The Ministry of Health very strongly supports this exciting and important work.

As you are aware, preventing new HIV infection, especially amongst adolescents, remains a priority to the Ministry of Health. Your proposal directly seeks to identify the causes of poor uptake and retention to PrEP amongst adolescents and ascertain different modalities for improving uptake and retention.

If successful, this proposal will compliment and accelerate the vision of the Ministry to end the HIV epidemic through targeted, age-appropriate access to prevention interventions.

Thank you for your commitment in supporting our health sector.

Yours sincerely,

Ms. Kakulubelwa C. Mulalelo
Permanent Secretary (A)
Ministry of Health

3. Additional Letter of Support from MOH regarding PREP uptake amongst adolescent girls

All Correspondence should be addressed to the Permanent Secretary
Telephone: +260 211 253040/5
Fax: +260 211 253344

In reply please quote:
MH/101/24/6

REPUBLIC OF ZAMBIA
MINISTRY OF HEALTH

NDEKE HOUSE
P. O. BOX 30205
LUSAKA

8th February, 2019

Carolyn Bolton Moore, MD, MSc
Centre for Infectious Disease Research in Zambia (CIDRZ)/
University of Alabama Birmingham, USA
Lusaka

REF: LETTER OF SUPPORT FOR HIH-FUNDED APPLICATION: "IMPROVING PrEP UPTAKE AND RETENTION AMONGST ADOLESCENT GIRLS IN LUSAKA, ZAMBIA

Dear Dr. Bolton Moore,

I am delighted to write this letter of support of the above-mentioned project. The Ministry of Health very strongly supports this exciting and important work.

As you are aware, preventing new HIV infection, especially amongst adolescents, remains a priority to the Ministry of Health. Your proposal directly seeks to identify the causes of poor uptake and retention to PrEP amongst adolescents and ascertain different modalities for improving uptake and retention.

If successful, this proposal will complement and accelerate the vision of the Ministry to end the HIV epidemic through targeted, age-appropriate access to prevention interventions.

You have the full support of the Ministry.

Dr. Kennedy Malama
Permanent Secretary – TS
MINISTRY OF HEALTH

CIDRZ CENTRE FOR INFECTIOUS DISEASE RESEARCH IN ZAMBIA
RECEIVED
13 FEB 2019
15:55

4. **Most Recent Ethics Approval Letter for the SKILLZ study**

UNIVERSITY OF ZAMBIA
BIOMEDICAL RESEARCH ETHICS COMMITTEE

Telephone: 260-1-256067
Telegrams: UNZA, LUSAKA
Telex: UNZALU ZA 44370
Fax: + 260-1-250753
Federal Assurance No. FWA00000338

Ridgeway Campus
P.O. Box 50110
Lusaka, Zambia
E-mail: unzarec@unza.zm
IRB00001131 of IORG0000774

28th April 2020.

CIDRZ CENTRE FOR INFECTIOUS DISEASE RESEARCH IN ZAMBIA
29 APR 2020
RECEIVED

Your Ref: 004-01-19.

Dr. Carolyn Bolton Moore,
CIDRZ,
P.O Box 34681,
Lusaka.

Dear Dr. Bolton Moore,

RE: REQUEST FOR REVIEW OF PROTOCOL AMENDMENT FOR THE STUDY ENTITLED: "REACHING 90 90 90 IN ADOLESCENTS IN ZAMBIA: USING ALL OUR SKILLZ"; SHORT TITLE: "SKILLZ", VERSION 1.4, 13 APRIL 2020 (REF. NO. 004-01-19)

We acknowledge receipt of additional amendments made to the follow up survey and the translated version of the survey in Bemba and Nyanja.

The amendments were reviewed and approved as follows:

1. Protocol changes:
   a. Clarified language on the description of the various interventions and eligibility, (Pages 10, 11, 15, 18).
   b. Allowed for the baseline survey to be re-issued after the pilot of the SKILLZ-Girl intervention to validate responses, (Page 21, 24).
   c. Clarified mechanisms by which follow-u procedures can be completed (including follow-up by phone and/or secure web link, (Page 21).
   d. Allowed for verbal consent to be received by parents following email confirmation of the same by UNZA BREC on the 25th February 2020, (Page 34-35).
   e. Included the additional risk of physical violence to participants and staff following the community unrest that took place in February, 2020 (Page 36).
2. Addition of a paragraph to clarify that the baseline survey may be repeated during the pilot phase of the study. New versions in English, Nyanja & Bemba have been provided.

5. References

1. Celum CL, Delany-Moretlwe S, McConnell M, et al. Rethinking HIV prevention to prepare for oral PrEP implementation for young African women. *J Int AIDS Soc.* 2015;18(4).
2. Venter F, Cowan F, Black V, Rebe K, Bekker L-G. Pre exposure prophylaxis in Southern Africa: feasible or not. *J Int AIDS Soc.* 2015;18(4 Suppl 3):19979.
3. Central Statistical Office (CSO) [Zambia] MoHMZ, and ICF International Zambia Demographic and Health Survey 2013-14. *Rockville, Maryland, USA: Central Statistical Office, Ministry of Health, and ICF International*
2014.
4. Assessment ZPBHI. Summary Sheet: Preliminary Findings - Zambia Population Based HIV Impact Assessment. December 2016. http://phia.icap.columbia.edu/wp-content/uploads/2016/09/ZAMBIA-Factsheet.FIN_.pdf.
5. Wilcher R, Petruney T, Reynolds HW, Cates W. From effectiveness to impact: contraception as an HIV prevention intervention. *Sexually transmitted infections.* 2008;84 Suppl 2:ii54-60.
6. UNICEF. *A Progress Report: ALL IN to end the adolescent AIDS epidemic.* Dec 2016 2016.
7. Zambia MoH. *Adolescent Health Strategy 2017 to 2021.* Lusaka 2017.
8. MacPherson P, Munthali C, Ferguson J, et al. Service delivery interventions to improve adolescents' linkage, retention and adherence to antiretroviral therapy and HIV care. *Tropical medicine & international health.* 2015;20(8):1015-1032.
9. Cluver L, Pantelic M, Orkin M, Toska E, Medley S, Sherr L. Sustainable Survival for adolescents living with HIV: do SDG-aligned provisions reduce potential mortality risk? *Journal of the International AIDS Society.* 2018;21:e25056.
10. Albert D, Chein J, Steinberg L. The teenage brain: Peer influences on adolescent decision making. *Current directions in psychological science.* 2013;22(2):114-120.
11. Christakou A, Brammer M, Rubia K. Maturation of limbic corticostriatal activation and connectivity associated with developmental changes in temporal discounting. *Neuroimage.* 2011;54(2):1344-1354.
12. Figner B, Mackinlay RJ, Wilkening F, Weber EU. Affective and deliberative processes in risky choice: age differences in risk taking in the Columbia Card Task. *Journal of Experimental Psychology: Learning, Memory, and Cognition.* 2009;35(3):709.
13. Olson EA, Hooper CJ, Collins P, Luciana M. Adolescents' performance on delay and probability discounting tasks: contributions of age, intelligence, executive functioning, and self-reported externalizing behavior. *Personality and individual differences.* 2007;43(7):1886-1897.
14. Pfeifer JH, Blakemore S-J. Adolescent social cognitive and affective neuroscience: past, present, and future. In: Oxford University Press; 2012.
15. Toska E, Pantelic M, Meinck F, Keck K, Haghighat R, Cluver L. Sex in the shadow of HIV: A systematic review of prevalence, risk factors, and interventions to reduce sexual risk-taking among HIV-positive adolescents and youth in sub-Saharan Africa. *PloS one.* 2017;12(6):e0178106.
16. World Health Organization. Consolidating guidelines on the use of antiretroviral drugs for treating and preventing HIV infection. In: Second ed.2016. Accessed March 20, 2017.
17. National Research Council and Institute of Medicine. *Adolescent Health Services: Missing Opportunities.* Washington, D.C: The National Academies Press; 2009.

18. Kaufman Z, Spencer T, Ross D. Effectiveness of sport-based HIV prevention interventions: a systematic review of the evidence. *AIDS and Behavior.* 2013;17(3):987-1001.
19. Malekaa EN. Monitoring and evaluation of sport-based HIV/AIDS awareness programmes: Strengthening outcome indicators. *SAHARA: Journal of Social Aspects of HIV/AIDS Research Alliance.* 2017;14(1):1-21.
20. Lall P, How Lim S, Khairuddin N, Kamarulzaman A. Review: an urgent need for research on factors impacting adherence to and retention in care among HIV-positive youth and adolescents from key populations. *J Int AIDS Soc.* 2015;18:41Á53.
21. Hudelson C, Cluver L. Factors associated with adherence to antiretroviral therapy among adolescents living with HIV/AIDS in low-and middle-income countries: a systematic review. *AIDS care.* 2015;27(7):805-816.
22. Lowenthal E, Lawler K, Harari N, et al. Rapid psychosocial function screening test identified treatment failure in HIV+ African youth. *AIDS care.* 2012;24(6):722-727.
23. Kim S-H, Gerver SM, Fidler S, Ward H. Adherence to antiretroviral therapy in adolescents living with HIV: systematic review and meta-analysis. *Aids.* 2014;28(13):1945-1956.
24. Bateganya M, Abdulwadud OA, Kiene SM. Home-based HIV voluntary counselling and testing (VCT) for improving uptake of HIV testing. *The Cochrane database of systematic reviews.* 2010(7):Cd006493.
25. Dickson KE, Ashton J, Smith JM. Does setting adolescent-friendly standards improve the quality of care in clinics? Evidence from South Africa. *International journal for quality in health care : journal of the International Society for Quality in Health Care.* 2007;19(2):80-89.
26. R-NASF. *Revised Zambia National HIV and AIDS Startegic Framework* 2014-2017.
27. Lee K LT, Zales M, Mayer S.

Scaling up a sport-based intervention model to increase HIV prevention, testing and treatment among Zambian youth. 3rd National HIV Prevention Convention – Abstract Submission; n.d.

28. Zales M, Kaufman ZA. A sport-based intervention to increase linkage to care. *AIDS conference, Washington DC* 2012.
29. Bärnighausen T, Tanser F. Rethinking the role of the local community in HIV epidemic spread in sub-Saharan Africa: a proximate-determinants approach. *HIV therapy.* 2009;3(5):435-445.
30. Bronfenbrenner U. Toward an experimental ecology of human development. *American psychologist.* 1977;32(7):513.
31. Mavedzenge SMN, Doyle AM, Ross DA. HIV prevention in young people in sub-Saharan Africa: a systematic review. *Journal of Adolescent Health.* 2011;49(6):568-586.
32. McCoy SI, Kangwende RA, Padian NS. Behavior change interventions to prevent HIV infection among women living in low and middle income countries: a systematic review. *AIDS and Behavior.* 2010;14(3):469-482.
33. Michielsen K, Chersich MF, Luchters S, De Koker P, Van Rossem R, Temmerman M. Effectiveness of HIV prevention for youth in sub-Saharan Africa: systematic review and meta-analysis of randomized and nonrandomized trials. *Aids.* 2010;24(8):1193-1202.
34. Morris JL, Rushwan H. Adolescent sexual and reproductive health: The global challenges. *International journal of gynaecology and obstetrics: the official organ of the International Federation of Gynaecology and Obstetrics.* 2015;131 Suppl 1:S40-42.

35. Bearinger LH, Sieving RE, Ferguson J, Sharma V. Global perspectives on the sexual and reproductive health of adolescents: patterns, prevention, and potential. *Lancet (London, England).* 2007;369(9568):1220-1231.
36. Bekker L-G, Johnson L, Wallace M, Hosek S. Building our youth for the future. *J Int aids Soc.* 2015;18:1Á7.
37. Dellar RC, Dlamini S, Karim QA. Adolescent girls and young women: key populations for HIV epidemic control. *J Int AIDS Soc.* 2015;18:64-70.
38. Fleischman J, Peck K. Addressing HIV Risk in Adolescent Girls and Young Women. *CSIS Global Health Policy Center.* 2015.
39. Hardee K, Gay J, Croce-Galis M, Afari-Dwamena NA. What HIV programs work for adolescent girls? *JAIDS Journal of Acquired Immune Deficiency Syndromes.* 2014;66:S176-S185.
40. Harrison A, Colvin CJ, Kuo C, Swartz A, Lurie M. Sustained high HIV incidence in young women in Southern Africa: social, behavioral, and structural factors and emerging intervention approaches. *Current HIV/AIDS Reports.* 2015;12(2):207-215.
41. Ramjee G, Daniels B. Women and HIV in sub-Saharan Africa. *AIDS research and therapy.* 2013;10(1):30.
42. Mburu G, Ram M, Oxenham D, Haamujompa C, Iorpenda K, Ferguson L. Responding to adolescents living with HIV in Zambia: a social–ecological approach. *Children and Youth Services Review.* 2014;45:9-17.
43. Lee S, Hazra R. Achieving 90-90-90 in pediatric HIV: adolescence as the touchstone for transition success. *J Int AIDS Soc.* 2015;18(Suppl 6):20257.
44. Moyo N, Müller JC. The influence of cultural practices on the HIV and AIDS pandemic in Zambia. *HTS Theological Studies.* 2011;67(3):412-417.
45. Burke HM, Mueller MP, Perry B, et al. Observational study of the acceptability of Sayana(R) Press among intramuscular DMPA users in Uganda and Senegal. *Contraception.* 2014;89(5):361-367.
46. Burke HM, Mueller MP, Packer C, et al. Provider acceptability of Sayana(R) Press: results from community health workers and clinic-based providers in Uganda and Senegal. *Contraception.* 2014;89(5):368-373.
47. Cover J, Ba M, Lim J, Drake JK, Daff BM. Evaluating the feasibility and acceptability of self-injection of subcutaneous depot medroxyprogesterone acetate (DMPA) in Senegal: a prospective cohort study. *Contraception.* 2017;96(3):203-210.
48. Center KE, Gunn JK, Asaolu IO, Gibson SJ, Ehiri JE. Contraceptive Use and Uptake of HIV-Testing among Sub-Saharan African Women. *PloS one.* 2016;11(4):e0154213.
49. Blanc AK, Tsui AO, Croft TN, Trevitt JL. Patterns and trends in adolescents' contraceptive use and discontinuation in developing countries and comparisons with adult women. *International perspectives on sexual and reproductive health.* 2009;35(2):63-71.
50. Smith P, Wallace M, Bekker LG. Adolescents' experience of a rapid HIV self-testing device in youth-friendly clinic settings in Cape Town South Africa: a cross-sectional community based usability study. *Journal of the International AIDS Society.* 2016;19(1):21111.
51. Taylor M, Jinabhai C, Dlamini S, Sathiparsad R, Eggers MS, De Vries H. Effects of a teenage pregnancy prevention program in KwaZulu-Natal, South Africa. *Health care for women international.* 2014;35(7-9):845-858.

52. Rosenberg NE, Bhushan NL, Vansia D, et al. Comparing Youth-Friendly Health Services to the Standard of Care Through "Girl Power-Malawi": A Quasi-Experimental Cohort Study. *Journal of acquired immune deficiency syndromes (1999).* 2018;79(4):458-466.
53. Kirby DB, Laris B, Rolleri LA. Sex and HIV education programs: their impact on sexual behaviors of young people throughout the world. *Journal of Adolescent Health.* 2007;40(3):206-217.
54. Hershow RB, Gannett K, Merrill J, et al. Using soccer to build confidence and increase HCT uptake among adolescent girls: a mixed-methods study of an HIV prevention programme in South Africa. *Sport in society.* 2015;18(8):1009-1022.
55. Zambia MoH. Zambia Consolidated Guidelines for Prevention and Treatment of HIV Infection In. Lusaka, Zambia: Republic of Zambia 2018.
56. Ivankova NV, Creswell JW, Stick SL. Using Mixed-Methods Sequential Explanatory Design: From Theory to Practice. *Field Methods.* 2006;18(1):3-20.
57. Nachega JB, Morroni C, Zuniga JM, et al. HIV-related stigma, isolation, discrimination, and serostatus disclosure: a global survey of 2035 HIV-infected adults. *Journal of the International Association of Physicians in AIDS Care.* 2012;11(3):172-178.
58. Cyril S, Smith BJ, Renzaho AM. Systematic review of empowerment measures in health promotion. *Health promotion international.* 2015;31(4):809-826.
59. Schwarzer R, Jerusalem M. The general self-efficacy scale (GSE). *Anxiety, Stress, and Coping.* 2010;12:329-345.
60. Upadhyay UD, Dworkin SL, Weitz TA, Foster DG. Development and validation of a reproductive autonomy scale. *Studies in family planning.* 2014;45(1):19-41.
61. Gopichandran V, Wouters E, Chetlapalli SK. Development and validation of a socioculturally competent trust in physician scale for a developing country setting. *BMJ open.* 2015;5(4):e007305.
62. Østergaard LR. Trust matters: A narrative literature review of the role of trust in health care systems in sub-Saharan Africa. *Global public health.* 2015;10(9):1046-1059.
63. Sandelowski M. Telling stories: Narrative approaches in qualitative research. *Journal of nursing scholarship.* 1991;23(3):161-166.
64. Valeri L, VanderWeele TJ. Mediation analysis allowing for exposure–mediator interactions and causal interpretation: Theoretical assumptions and implementation with SAS and SPSS macros. *Psychological methods.* 2013;18(2):137.
65. Tingley D YT, Hirose K, Keele L, Imai K. Mediation: R package for causal mediation analysis. 2014.
66. Braun V, Clarke V. Using thematic analysis in psychology. *Qualitative research in psychology.* 2006;3(2):77-101.
67. Glasgow RE, Vogt TM, Boles SM. Evaluating the public health impact of health promotion interventions: the RE-AIM framework. *American journal of public health.* 1999;89(9):1322-1327.
