# Supplementary material for: Evaluation of the SKILLZ intervention to promote HIV testing and contraception uptake in adolescent girls in Lusaka, Zambia: A cluster-randomized trial
Source: PLOS Glob Public Health. 2025 Oct 29;5(10):e0005375. doi: 10.1371/journal.pgph.0005375 (PMC12571267; doi:10.1371/journal.pgph.0005375)
Supplement: S1 Checklist — The CONSERVE implementation tool is licensed under the Creative Commons Attribution-NonCommercial-NoDerivatives 4.0 International license. (DOCX) [file pgph.0005375.s003.docx]

# CONSERVE Checklists

Use CONSERVE-CONSORT for completed trial reports and CONSERVE-SPIRIT for trial protocols.

| CONSERVE-CONSORT Extension: [DATE] | | | | | |
| --- | --- | --- | --- | --- | --- |
| Item | Item Title | Description | | | Page No. |
| I. | Extenuating Circumstances | Describe the circumstances and how they constitute extenuating circumstances. | | | Lines 128-133 |
| II. | Important Modifications | 1. Describe how the modifications are important modifications. | | | Lines 156-160 |
|  |  | 1. Describe the impacts and mitigating strategies, including their rationale and implications for the trial. | | | Lines 207-213 |
|  |  | 1. Provide a modification timeline. | | | Line 642-651 |
| III. | Responsible Parties | State who planned, reviewed and approved the modifications. | | | Line 134-136 |
| IV. | Interim data | If modifications were informed by trial data, describe how the interim data were used, including whether they were examined by study group, and whether the individuals reviewing the data were blinded to the treatment allocation. | | | N/A |
| CONSORT Number and Item | | For each row, if important modifications occurred check “direct impact” and/or “mitigating strategy” and describe the changes in the trial manuscript or supplement. Check “no change” for items that are unaffected in the extenuating circumstance. | | | Page No. |
|  |  | No Change | Impact* | Mitigating Strategy** |  |
| 1 | Title and abstract | X |  |  |  |
| 2 | Introduction | X |  |  |  |
| 3 | Methods: Trial Design |  | X |  | Lines 128-133 |
| 4 | Methods: Participants |  | X |  | Lines 156-160 |
| 5 | Methods: Interventions |  | X |  | Lines 207-213 |
| 6 | Methods: Outcomes | X |  |  |  |
| 7 | Methods: Sample Size |  | X | X | Lines 156-160; Lines 227-234 |
| 8-10 | Methods: Randomisation | X |  |  |  |
| 11 | Methods: Blinding | X |  |  |  |
| 12 | Methods: Statistical methods | X |  |  |  |
| 13 | Results: Participant flow | X |  |  |  |
| 14 | Results: Recruitment | X |  |  |  |
| 15 | Results: Baseline data | X |  |  |  |
| 16 | Results: Numbers analysed |  | X | X | Lines 277-287 |
| 17 | Results: Outcomes and estimation | X |  |  |  |
| 18 | Results: Ancillary analyses | X |  |  |  |
| 19 | Results: Harms | X |  |  |  |
| 20 | Discussion: Limitations |  | X |  | Lines 431-435 |
| 21 | Discussion: Generalisability |  | X |  | Lines 431-435, 457-463 |
| 23 | Other information: Registration | X |  |  |  |
| 24 | Other information: Protocol | X |  |  |  |
| 25 | Other information: Funding | X |  |  |  |
| *Aspects of the trial that are directly affected or changed by the extenuating circumstance and are not under the control of investigators, sponsor or funder.  **Aspects of the trial that are modified by the study investigators, sponsor or funder to respond to the extenuating circumstance or manage the direct impacts on the trial. | | | | | |
